# Highly Luminescent 4*H*-1,2,4-Triazole Derivatives: Synthesis, Molecular Structure and Photophysical Properties

**DOI:** 10.3390/ma13245627

**Published:** 2020-12-10

**Authors:** Monika Olesiejuk, Agnieszka Kudelko, Marcin Świątkowski

**Affiliations:** 1 Department of Chemical Organic Technology and Petrochemistry, The Silesian University of Technology, Krzywoustego 4, PL-44100 Gliwice, Poland; agnieszka.kudelko@polsl.pl; 2 Department of X-ray Crystallography and Crystal Chemistry, Institute of General and Ecological Chemistry, Lodz University of Technology, Żeromskiego 116, PL-90924 Łódź, Poland; marcin.swiatkowski@p.lodz.pl

**Keywords:** 1,2,4-triazole derivatives, Suzuki coupling, ionic liquids, microwaves, luminescent properties

## Abstract

An alternative approach to the Suzuki cross-coupling reaction is used to synthesize a series of new luminophores based on 4-alkyl-4*H*-1,2,4-triazole cores conjugated via 1,4-phenylene linker to fused-bicyclic and tricyclic aromatic, or heteroaromatic arrangements. The described methodology allows one to conduct the coupling reaction with the use of commercially available boronic acids in the presence of conventional solvents or ionic liquids and produced excellent yields. It was found that the use of ultrasounds or microwaves significantly accelerates the reaction. The obtained compounds exhibited high luminescent properties and a large quantum yield of emitted photons. The X-ray molecular structures of three highly conjugated 4*H*-1,2,4-triazole representatives are also presented.

## 1. Introduction

In recent decades, organic compounds containing both donor and acceptor moieties connected through π-conjugated linkages have been widely investigated due to the possibility of their application in optoelectronic devices [1,2,3], such as organic light-emitting diodes (OLEDs), photovoltaic cells and organic field-effect transistors (OFETs). However, a potential luminophore should contain not only an extended π-conjugated system, but also other features including the proper electron–hole transporting properties, a high external quantum efficiency, as well as thermal and chemical stabilities [4,5]. Heterocyclic arrangements with highly electronegative nitrogen atoms perfectly fit the requirements for such systems. The presence of nitrogen in the aromatic ring considerably affects the electron distribution within the molecule, and also improves the intramolecular electron transport.

1,2,4-Triazoles belong to the vast family of the five-membered heterocyclic arrangements and are especially interesting due to their high nitrogen content. This scaffold occurs in two tautomeric forms with the 1*H*-1,2,4-triazole system predominating over the 4*H*-1,2,4-triazole form [6,7]. The derivatives of 4*H*-1,2,4-triazole exhibit interesting properties and, thus, have applications in diverse fields such as optoelectronics (emission properties) [8,9,10,11,12,13,14], materials science (corrosion inhibition) [15,16,17,18,19,20], medicine [21,22,23,24,25,26,27,28,29,30,31,32] and agriculture [33,34,35,36,37,38] (biological properties). Due to the numerous applications, the 1,2,4-triazole core has been the subject of several recent literature reviews commenting on the synthesis of these moieties [7,39,40,41,42,43]. The most popular methods for the construction of trisubstituted 4*H*-1,2,4-triazole derivatives are the cyclocondensation of *N*,*N’*-dichloromethylidenehydrazine using an appropriate amine, and the cyclodehydration of *N*-acylamidrazone derivatives from a large variety of precursors depending on the nature of their substituents [7,30,40]. An interesting alternative seems to be the use of the one-pot method in the synthesis of 4*H*-1,2,4-triazole derivatives [44,45,46].

One of the most effective reactions to form a C–C bond are cross-coupling reactions. Among them, the methodology using a variety of commercially available organoboron compounds is particularly interesting. The Suzuki cross-coupling reaction is typically completed with various combinations of catalysts, bases or solvents, and their mutual integration affects the efficiency and selectivity of the reaction [47,48,49]. Suzuki cross-coupling reaction is well utilized by chemists due to the possibility of using alternative solvents such as ionic liquids (IL), or ultrasound and microwave irradiation as accelerating factors. A combination of these two aspects, such as microwave or ultrasound-assisted reactions using IL, are necessary to improve the environmental impact of chemistry. In the case of alternative solvents, the important topic worth mentioning is the low vapor pressure of the ionic liquid, and the possibility of its regeneration and reuse, which eliminates classic volatile organic solvents. It is possible to reduce energy expenditure by heating chemical reactions using microwaves or by reducing the number of reagents applied when the ionic liquid acts as a catalyst [50,51,52,53,54].

In our previous work, we analyzed the derivatives of five-membered heterocyclic systems such as oxadiazole, thiadiazole, and triazole, with potential applications in optoelectronics [55,56,57,58]. We showed that 1,2,4-triazole derivatives have very good emission properties. Here, we present a synthesis proceeding in an environmentally friendly manner of more extended π-conjugated systems with triazole core hoping for their equally high quantum yields of fluorescence.

## 2. Materials and Methods

### 2.1. General Information

The melting points of all compounds were measured on a Stuart SMP3 melting point apparatus (Stuart, Staffordshire, UK). The ^1^H-NMR and ^13^C-NMR spectra were performed in CDCl_3_ solution using tetramethylsilane (TMS) as the internal standard on an Agilent 400-NMR spectrometer (Agilent Technologies, Waldbronn, Germany). X-ray diffraction data were collected on the Synergy Dualflex Pilatus diffractometer (Rigaku, Tokyo, Japan) (for the detailed description of the crystal structure determination, see Supplementary Materials, Section 3). All FT-IR spectra were registered between 4000 and 650 cm^−1^ using an FT-IR Nicolet 6700 apparatus with a Smart iTR accessory (Thermo Fischer Scientific, Wesel, Germany). UV–Vis spectra were recorded on a Jasco V-660 spectrophotometer (Jasco Corporation, Tokyo, Japan) at room temperature in CH_2_Cl_2_ (c = 5 × 10^−6^ mol/dm^3^). Fluorescence spectra were recorded at room temperature in CH_2_Cl_2_ (c = 5 × 10^−6^ mol/dm^3^) using a Jasco F-6300 fluorescence spectrophotometer (Jasco Corporation, Tokyo, Japan). High-resolution mass spectra measurements were completed on a Waters ACQUITY UPLC/Xevo G2QT instrument (Waters Corporation, Milford, MA, USA). Thin-layer chromatography was performed on silica gel 60 F_254_ Thin-Layer Chromatography (TLC) plates (Merck KGaA, Darmstadt, Germany) using CHCl_3_/EtOAc (5:1 *v*/*v*) as the mobile phase. All reagents from commercial sources were used without additional purification. An aqueous solution of IL: choline hydroxide solution 46 wt.% in H_2_O (Choline–OH) was used throughout. Ultrasound, for sonication, was generated using an EMAG Technologies Emmi 40HC operating at a frequency 38 kHz, 230 V/50 Hz with bath dimensions of 300 mm × 155 mm × 100 mm (Emag Polska, Juszczyn, Poland). The experiments using microwave radiation were performed in a CEM Discover microwave-enhanced synthesis system (CEM Corporation, Matthews, NC, USA) equipped with a personal computer.

### 2.2. Synthesis of Compounds

#### 2.2.1. The Synthesis of Suzuki Cross-Coupling Precursors

**Scheme 1:**
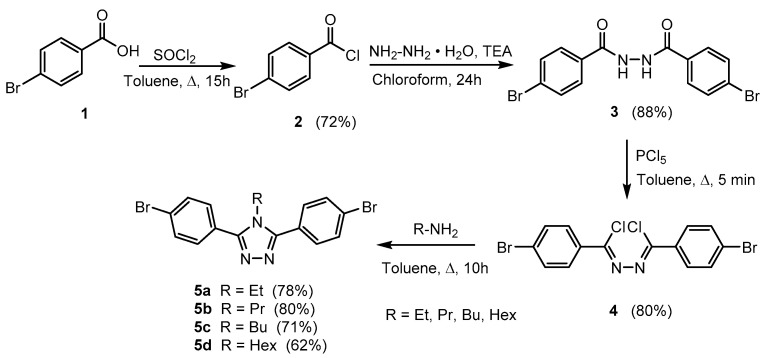
Synthesis of the 4-alkyl-4*H*-1,2,4-triazole precursors.

4-alkyl-3,5-bis(4-bromophenyl)-4*H*-1,2,4-triazoles (**5a–d**) was completed according to the literature procedure [58] using a four-step reaction methodology starting with commercially available 4-bromobenzoic acid (**1**) (Scheme 1).

#### 2.2.2. A General Procedure for Conventional Suzuki Cross-Coupling Reactions

Synthesis of the 4-alkyl-3,5-bis[4′-(*N*,*N*-diphenylamino)biphenyl-4-yl]-4*H*-1,2,4-triazoles (**7–10a**).

4-Alkyl-3,5-bis(4-bromophenyl)-4*H*-1,2,4-triazole (**5a–d**, 1.00 mmol), 4-(*N*,*N*-diphenylamino) phenylboronic acid (**6a**, 2.50 mmol), palladium catalyst Pd(PPh_3_)_4_ (0.058 g, 0.05 mmol), a phase transfer catalyst NBu_4_Br (0.032 g, 0.10 mmol), and base K_2_CO_3_ (1.382 g, 10.00 mmol) were added to a combination of toluene (9 mL), H_2_O (6 mL) and EtOH (3 mL). The mixture was heated under reflux in an oil bath (130 °C) for 7 h (TLC). After cooling, the mixture was transferred to a separating funnel and chloroform (50 mL) was added. After additional extraction with chloroform (2 × 10 mL), the combined organic layers were filtered through a silica gel plug (10 mL). The silica layer was then rinsed with CHCl_3_/EtOAc (5:1 *v*/*v*). The filtrate was dried with MgSO_4_ and concentrated on the rotary evaporator. The product was precipitated using EtOAc (5 mL), filtered, washed with fresh EtOAc, and air-dried to give pure 4-alkyl-3,5-bis[4′-(*N*,*N*-diphenylamino)biphenyl-4-yl]-4*H*-1,2,4-triazoles (**7–10a**).

#### 2.2.3. A General Procedure for IL Alternative Approach for Suzuki Cross-Coupling Reactions

Synthesis of the 4-alkyl-3,5-bis(4-arylphenyl)-4*H*-1,2,4-triazoles **7a–k**, **8a**, **9a–k**, **10a**.

4-Alkyl-3,5-bis(4-bromophenyl)-4*H*-1,2,4-triazole (**5a–d**, 1.00 mmol), an appropriate boronic acid (**6a–k**, 2.50 mmol), and Pd(PPh_3_)_4_ (0.058 g, 0.05 mmol) were added to a mixture of aqueous choline hydroxide solution 46 wt.% (Choline–OH, 10 mL) and toluene (1 mL). The reaction monitored by TLC was carried out using conventional heating—an oil bath (130 °C) or ultrasonication—an ultrasonic bath (80 °C), or microwave irradiation (150 W) in 3–5 cycles of 90 s with 2 min intervals at a temperature 50–100 °C. After cooling, mixture was transferred to a separating funnel and chloroform (50 mL) was added. After the additional extraction with chloroform (2 × 10 mL), the combined chloroform layers were filtered through a silica gel plug (10 mL). The silica layer was then rinsed with CHCl_3_/EtOAc (5:1 *v*/*v*). The filtrate was dried with MgSO_4_ and concentrated on the rotary evaporator. The product was precipitated using EtOAc (5 mL), filtered, washed with fresh EtOAc, and air-dried to give corresponding 4-alkyl-3,5-bis(4-arylphenyl)-4*H*-1,2,4-triazoles **7a–k**, **8a**, **9a–k**, **10a**.

### 2.3. Characterization of Compounds

4-Ethyl-3,5-bis[4′-(*N,N*-diphenylamino)biphenyl-4-yl]-4*H*-1,2,4-triazole (**7a**). Yellow solid in 97% yield, 0.357 g, m.p. 268–270 °C; UV (CH_2_Cl_2_) λ_max_ 351.0 nm (ε⋅10^−3^ 59.5 cm^−1^M^−1^); IR (ATR) ν: 3033, 1731, 1588, 1509, 1483, 1327, 1272, 1219, 1181, 1075, 1028, 821, 772, 749, 694, 666, 621 cm^−1^; ^1^H-NMR (400 MHz, CDCl_3_): δ 1.16 (t, *J* = 7.2 Hz, 3H, CH_3_), 4.23 (q, *J* = 7.2 Hz, 2H, CH_2_), 7.06 (t, *J* = 7.2 Hz, 4H, ArH), 7.13–7.18 (m, 12H, ArH), 7.28 (t, *J* = 7.2 Hz, 8H, ArH), 7.54 (d, *J* = 8.4 Hz, 4H, ArH), 7.71–7.75 (m, 8H, ArH); ^13^C-NMR (100 MHz, CDCl_3_): δ 15.8, 40.0, 123.2, 123.6, 124.6, 126.0, 126.9, 127.8, 129.3, 129.4, 133.5, 142.2, 147.5, 147.9, 155.1; HRMS *m/z* calcd for (C_52_H_41_N_5_ + H^+^): 736.3440; found: 736.3441.

4-Ethyl-3,5-bis[4-(naphthalen-1-yl)phenyl]-4*H*-1,2,4-triazole (**7b**). Beige solid in 88% yield, 0.221 g, m.p. 308–310 °C; UV (CH_2_Cl_2_) λ_max_ 297.0 nm (ε⋅10^−3^ 36.4 cm^−1^M^−1^); IR (ATR) ν: 3055, 2980, 1591, 1506, 1478, 1425, 1395, 1340, 1250, 1108, 1018, 967, 951, 829, 802, 793, 780, 754, 729 cm^−1^; ^1^H-NMR (400 MHz, CDCl_3_): δ 1.29 (t, *J* = 7.2 Hz, 3H, CH_3_), 4.36 (q, *J* = 7.2 Hz, 2H, CH_2_), 7.47–7.59 (m, 8H, ArH), 7.69 (d, *J* = 8.4 Hz, 4H, ArH), 7.86 (d, *J* = 8.4 Hz, 4H, ArH), 7.91–7.96 (m, 6H, ArH); ^13^C-NMR (100 MHz, CDCl_3_): δ 16.1, 40.1, 125.4, 125.7, 126.0, 126.4, 126.7, 127.0, 128.2, 128.4, 128.9, 130.7, 131.4, 133.8, 139.2, 142.7, 155.3; HRMS *m/z* calcd for (C_36_H_27_N_3_ + H^+^): 502.2283; found: 502.2281.

4-Ethyl-3,5-bis[4-(naphthalen-2-yl)phenyl]-4*H*-1,2,4-triazole (**7c**). White solid in 99% yield, 0.248 g, m.p. 292–295 °C; UV (CH_2_Cl_2_) λ_max_ 268.0 nm (ε⋅10^−3^ 57.3 cm^−1^M^−1^) and 305.0 (45.0); IR (ATR) ν: 3051, 1473, 1423, 1401, 1360, 1211, 1080, 1017, 969, 956, 898, 857, 815, 752, 719 cm^−1^; ^1^H-NMR (400 MHz, CDCl_3_): δ 1.22 (t, *J* = 7.2 Hz, 3H, CH_3_), 4.29 (q, *J* = 7.2 Hz, 2H, CH_2_), 7.50–7.57 (m, 4H, ArH), 7.81 (dd, *J* = 8.4 and 1.6 Hz, 2H, ArH), 7.84 (d, *J* = 8.8 Hz, 4H, ArH), 7.88–7.95 (m, 8H, ArH), 7.97 (d, *J* = 8.4 Hz, 2H, ArH), 8.13 (d, *J* = 1.6 Hz, 2H, ArH); ^13^C-NMR (100 MHz, CDCl_3_): δ 16.0, 40.1, 125.2, 126.1, 126.3, 126.5, 126.7, 127.7, 127.9, 128.3, 128.7, 129.4, 132.9, 133.6, 137.4, 142.8, 155.2; HRMS *m/z* calcd for (C_36_H_27_N_3_ + H^+^): 502.2283; found: 502.2287.

4-Ethyl-3,5-bis[4-(quinolin-3-yl)phenyl]-4*H*-1,2,4-triazole (**7d**). White solid in 79% yield, 0.198 g, m.p. 296–298 °C; UV (CH_2_Cl_2_) λ_max_ 269.0 nm (ε⋅10^−3^ 52.8 cm^−1^M^−1^) and 305.0 (42.6); IR (ATR) ν: 3069, 3002, 2964, 1494, 1477, 1468, 1453, 1379, 1358, 1543, 1202, 1081, 1019, 963, 952, 931, 912, 868, 856, 841, 795, 781, 767, 761, 744, 738, 726 cm^−1^; ^1^H-NMR (400 MHz, CDCl_3_): δ 1.23 (t, *J* = 7.2 Hz, 3H, CH_3_), 4.30 (q, *J* = 7.2 Hz, 2H, CH_2_), 7.62 (t, *J* = 8.0 Hz, 2H, ArH), 7.77 (t, *J* = 8.0 Hz, 2H, ArH), 7.88–7.94 (m, 10H, ArH), 8.18 (d, *J* = 8.0 Hz, 2H, ArH), 8.40 (d, *J* = 2.4 Hz, 2H, ArH), 9.25 (d, *J* = 2.4 Hz, 2H, ArH); ^13^C-NMR (100 MHz, CDCl_3_): δ 16.0, 40.1, 127.3, 127.4, 127.8, 127.9, 128.1, 129.3, 129.7, 129.8, 132.7, 133.6, 139.7, 147.7, 149.5, 155.0; HRMS *m/z* calcd for (C_34_H_25_N_5_ + H^+^): 504.2188; found: 504.2188.

4-Ethyl-3,5-bis[4-(quinolin-6-yl)phenyl]-4*H*-1,2,4-triazole (**7e**). Pearl solid in 94% yield, 0.236 g, m.p. 302–305 °C; UV (CH_2_Cl_2_) λ_max_ 269.0 nm (ε⋅10^−3^ 56.4 cm^−1^M^−1^) and 306.0 (42.0); IR (ATR) ν: 3052, 1593, 1570, 1499, 1474, 1424, 1347, 1326, 1121, 1014, 971, 892, 868, 856, 831, 797, 781, 754 cm^−1^; ^1^H-NMR (400 MHz, CDCl_3_): δ 1.22 (t, *J* = 7.2 Hz, 3H, CH_3_), 4.30 (q, *J* = 7.2 Hz, 2H, CH_2_), 7.47 (dd, *J* = 8.4 and 4.4 Hz, 2H, ArH), 7.85 (d, *J* = 8.4 Hz, 4H, ArH), 7.91 (d, *J* = 8.4 Hz, 4H, ArH), 8.05 (dd, *J* = 8.4 and 2.0 Hz, 2H, ArH), 8.09 (d, *J* = 2.0 Hz, 2H, ArH), 8.22–8.26 (m, 4H, ArH), 8.96 (dd, *J* = 4.4 and 2.0 Hz, 2H, ArH); ^13^C-NMR (100 MHz, CDCl_3_): δ 16.0, 40.1, 121.7, 125.8, 127.0, 128.0, 128.5, 128.9, 129.5, 130.2, 136.3, 138.1, 142.0, 147.9, 150.8, 155.1; HRMS *m/z* calcd for (C_34_H_25_N_5_ + H^+^): 504.2188; found: 504.2189.

3,5-Bis[4-(dibenzothiophen-4-yl)phenyl]-4-ethyl-4*H*-1,2,4-triazole (**7f**). Creamy solid in 96% yield, 0.296 g, m.p. 281–283 °C; UV (CH_2_Cl_2_) λ_max_ 240.0 nm (ε⋅10^−3^ 85.0 cm^−1^M^−1^) and 290.0 (48.5); IR (ATR) ν: 3062, 1470, 1440, 1425, 1391, 1376, 1360, 1246, 1112, 1016, 863, 836, 800, 753, 743, 730, 718, 704 cm^−1^; ^1^H-NMR (400 MHz, CDCl_3_): δ 1.28 (t, *J* = 7.2 Hz, 3H, CH_3_), 4.35 (q, *J* = 7.2 Hz, 2H, CH_2_), 7.48–7.51 (m, 4H, ArH), 7.56 (d, *J* = 7.6 Hz, 2H, ArH), 7.61 (t, *J* = 7.6 Hz, 2H, ArH), 7.86 (d, *J* = 7.6 Hz, 2H, ArH), 7.90 (d, *J* = 8.4 Hz, 4H, ArH), 7.95 (d, *J* = 8.4 Hz, 4H, ArH), 8.20–8.23 (m, 4H, ArH); ^13^C-NMR (100 MHz, CDCl_3_): δ 16.0, 40.1, 121.0, 121.8, 122.7, 124.5, 125.2, 127.0, 127.3, 128.9, 129.4, 135.7, 135.9, 136.5, 138.5, 139.4, 142.4, 152.2, 155.1; HRMS *m/z* calcd for (C_40_H_27_N_3_S_2_ + H^+^): 614.1725; found: 614.1727.

3,5-Bis[4-(dibenzofuran-4-yl)phenyl]-4-ethyl-4*H*-1,2,4-triazole (**7g**). Creamy solid in 92% yield, 0.267 g, m.p. 255–256 °C; UV (CH_2_Cl_2_) λ_max_ 286.0 nm (ε⋅10^−3^ 70.5 cm^−1^M^−1^) and 315.0 (41.0); IR (ATR) ν: 3055, 2949, 1586, 1479, 1450, 1430, 1412, 1395, 1360, 1259, 1189, 1101, 1058, 1014, 968, 874, 838, 801, 791, 741, 729 cm^−1^; ^1^H-NMR (400 MHz, CDCl_3_): δ 1.27 (t, *J* = 7.2 Hz, 3H, CH_3_), 4.35 (q, *J* = 7.2 Hz, 2H, CH_2_), 7.39 (t, *J* = 8.0 Hz, 2H, ArH), 7.46–7.52 (m, 4H, ArH), 7.65 (d, *J* = 8.0 Hz, 2H, ArH), 7.70 (d, *J* = 8.0 Hz, 2H, ArH), 7.91 (d, *J* = 8.4 Hz, 4H, ArH), 7.99–8.03 (m, 4H, ArH), 8.14 (d, *J* = 8.4 Hz, 4H, ArH); ^13^C-NMR (100 MHz, CDCl_3_): δ 16.1, 40.1, 111.9, 120.3, 120.8, 123.0, 123.4, 124.1, 124.7, 125.2, 126.8, 127.0, 127.4, 129.2, 129.3, 138.2, 153.3, 155.2, 156.2; HRMS *m/z* calcd for (C_40_H_27_N_3_O_2_ + H^+^): 582.2182; found: 582.2183.

4-Ethyl-3,5-bis[4-(9-methyl-9*H*-carbazol-3-yl)phenyl]-4*H*-1,2,4-triazole (**7h**). Creamy solid in 70% yield, 0.107 g, m.p. 313–315 °C; UV (CH_2_Cl_2_) λ_max_ 300.0 nm (ε⋅10^−3^ 69.9 cm^−1^M^−1^) and 321.0 (46.0); IR (ATR) ν: 3054, 2990, 2935, 1598, 1483, 1466, 1424, 1359, 1337, 1323, 1294, 1254, 1221, 1120, 1012, 844, 825, 804, 764, 750, 739, 719 cm^−1^; ^1^H-NMR (400 MHz, CDCl_3_): δ 1.22 (t, *J* = 7.2 Hz, 3H, CH_3_), 3.92 (s, 6H, CH_3_), 4.29 (q, *J* = 7.2 Hz, 2H, CH_2_), 7.29 (t, *J* = 7.6 Hz, 2H, ArH), 7.45 (d, *J* = 7.6 Hz, 2H, ArH), 7.50–7.55 (m, 4H, ArH), 7.80–7.83 (m, 6H, ArH), 7.91 (d, *J* = 8.4 Hz, 4H, ArH), 8.18 (d, *J* = 7.6 Hz, 2H, ArH), 8.40 (s, 2H, ArH); ^13^C-NMR (100 MHz, CDCl_3_): δ 15.9, 29.3, 40.0, 108.7, 108.9, 118.9, 119.2, 120.4, 122.9, 123.4, 125.1, 125.7, 126.1, 127.6, 129.4, 131.1, 140.9, 141.5, 143.8, 155.3; HRMS *m/z* calcd for (C_42_H_33_N_5_ + H^+^): 608.2814; found: 608.2817.

4-Ethyl-3,5-bis[4-(9-ethyl-9*H*-carbazol-3-yl)phenyl]-4*H*-1,2,4-triazole (**7i**). Creamy solid in 76% yield, 0.121 g, m.p. 232–234 °C; UV (CH_2_Cl_2_) λ_max_ 243.0 nm (ε⋅10^−3^ 54.6 cm^−1^M^−1^), 301.0 (76.5) and 322.0 (49.9); IR (ATR) ν: 3047, 2977, 1597, 1459, 1379, 1346, 1334, 1295, 1254, 1233, 1196, 1154, 1125, 1086, 848, 806, 769, 747, 729, 695 cm^−1^; ^1^H-NMR (400 MHz, CDCl_3_): δ 1.22 (t, *J* = 7.2 Hz, 3H, CH_3_), 1.49 (t, *J* = 7.2 Hz, 6H, CH_3_), 4.29 (q, *J* = 7.2 Hz, 2H, CH_2_), 4.43 (q, *J* = 7.2 Hz, 4H, CH_2_), 7.28 (t, *J* = 7.6 Hz, 2H, ArH), 7.45 (d, *J* = 7.6 Hz, 2H, ArH), 7.49–7.53 (m, 4H, ArH), 7.79 (d, *J* = 7.6 Hz, 2H, ArH), 7.83 (d, *J* = 8.4 Hz, 4H, ArH), 7.90 (d, *J* = 8.4 Hz, 4H, ArH), 8.18 (d, *J* = 7.6 Hz, 2H, ArH), 8.40 (s, 2H, ArH); ^13^C-NMR (100 MHz, CDCl_3_): δ 13.8, 15.9, 37.7, 40.0, 108.7, 108.9, 119.0, 119.1, 120.5, 123.0, 123.6, 125.1, 125.7, 126.0, 127.6, 129.3, 131.0, 139.8, 140.5, 143.8, 155.3; HRMS *m/z* calcd for (C_44_H_37_N_5_ + H^+^): 636.3127; found: 636.3124.

3,5-Bis[4′-(9*H*-carbazol-9-yl)biphenyl-4-yl]-4-ethyl-4*H*-1,2,4-triazole (**7j**). Grey solid in 77% yield, 0.141 g, m.p. 277–280 °C; UV (CH_2_Cl_2_) λ_max_ 293.0 nm (ε⋅10^−3^ 50.0 cm^−1^M^−1^) and 322.0 (41.7); IR (ATR) ν: 3055, 1740, 1599, 1521, 1477, 1450, 1414, 1367, 1334, 1318, 1303, 1227, 1184, 1170, 1119, 1004, 969, 825, 742, 719 cm^−1^; ^1^H-NMR (400 MHz, CDCl_3_): δ 1.25 (t, *J* = 7.2 Hz, 3H, CH_3_), 4.32 (q, *J* = 7.2 Hz, 2H, CH_2_), 7.32 (t, *J* = 7.6 Hz, 4H, ArH), 7.45 (t, *J* = 7.6 Hz, 4H, ArH), 7.51 (d, *J* = 7.6 Hz, 4H, ArH), 7.72 (d, *J* = 8.4 Hz, 4H, ArH), 7.86–7.92 (m, 12H, ArH), 8.19 (d, *J* = 7.6 Hz, 4H, ArH); ^13^C-NMR (100 MHz, CDCl_3_): δ 16.0, 40.1, 109.8, 120.1, 120.4, 123.5, 126.0, 126.9, 127.5, 127.6, 128.6, 129.5, 137.6, 139.0, 140.8, 142.0, 155.1; HRMS *m/z* calcd for (C_52_H_37_N_5_ + H^+^): 732.3127; found: 732.3126.

4-Ethyl-3,5-bis[4-(thiantren-1-yl)phenyl]-4*H*-1,2,4-triazole (**7k**). Grey solid in 87% yield, 0.585 g, m.p. 198–200 °C; UV (CH_2_Cl_2_) λ_max_ 263.0 nm (ε⋅10^−3^ 53.9 cm^−1^M^−1^); IR (ATR) ν: 3051, 1475, 1441, 1425, 1396, 1190, 1107, 1017, 967, 853, 842, 787, 748, 728, 686, 664, 653, 604 cm^−1^; ^1^H-NMR (400 MHz, CDCl_3_): δ 1.30 (t, *J* = 7.2 Hz, 3H, CH_3_), 4.35 (q, *J* = 7.2 Hz, 2H, CH_2_), 7.20 (t, *J* = 7.6 Hz, 2H, ArH), 7.26 (t, *J* = 7.6 Hz, 2H, ArH), 7.30–7.36 (m, 4H, ArH), 7.39 (d, *J* = 7.6 Hz, 2H, ArH), 7.51 (d, *J* = 7.6 Hz, 2H, ArH), 7.57 (d, *J* = 7.6 Hz, 2H, ArH), 7.60 (d, *J* = 8.4 Hz, 4H, ArH), 7.84 (d, *J* = 8.4 Hz, 4H, ArH); ^13^C-NMR (100 MHz, CDCl_3_): δ 16.0, 40.1, 127.2, 127.7, 127.9, 128.3, 128.6, 128.6, 128.7, 128.9, 129.1, 130.1, 135.0, 135.6, 135.9, 136.2, 141.5, 142.0, 155.2; HRMS *m/z* calcd for (C_40_H_27_N_3_S_4_ + H^+^): 678.1166; found: 678.1166.

3,5-Bis[4′-(*N,N*-diphenylamino)biphenyl-4-yl]-4-propyl-4*H*-1,2,4-triazole (**8a**). Yellow solid in 93% yield, 0.347 g, m.p. 189–192 °C; UV (CH_2_Cl_2_) λ_max_ 352.0 nm (ε⋅10^−3^ 51.8 cm^−1^M^−1^); IR (ATR) ν: 3033, 2958, 1735, 1587, 1509, 1486, 1327, 1272, 1241, 1179, 1046, 1004, 862, 843, 819, 773, 753, 737, 693 cm^−1^; ^1^H-NMR (400 MHz, CDCl_3_): δ 0.66 (t, *J* = 7.2 Hz, 3H, CH_3_), 1.49 (sext, *J* = 7.2 Hz, 2H, CH_2_), 4.13 (t, *J* = 7.2 Hz, 2H, CH_2_), 7.06 (t, *J* = 7.2 Hz, 4H, ArH), 7.14–7.18 (m, 12H, ArH), 7.28 (t, *J* = 7.2 Hz, 8H, ArH), 7.55 (d, *J* = 8.4 Hz, 4H, ArH), 7.71–7.75 (m, 8H, ArH); ^13^C-NMR (100 MHz, CDCl_3_): δ 10.7, 23.4, 46.6, 123.2, 123.6, 124.6, 126.1, 126.9, 127.7, 129.3, 129.4, 133.5, 142.1, 147.5, 147.8, 155.5; HRMS *m/z* calcd for (C_53_H_43_N_5_ + H^+^): 750.3597; found: 750.3588.

4-Butyl-3,5-bis[4′-(*N,N*-diphenylamino)biphenyl-4-yl]-4*H*-1,2,4-triazole (**9a**). Creamy solid in 99% yield, 0.382 g, m.p. 208–209 °C; UV (CH_2_Cl_2_) λ_max_ 351.0 nm (ε⋅10^−3^ 52.5 cm^−1^M^−1^); IR (ATR) ν: 3034, 2959, 2864, 1735, 1586, 1487, 1326, 1273, 1239, 1178, 1046, 1004, 974, 861, 843, 821, 754, 737, 693 cm^−1^; ^1^H-NMR (400 MHz, CDCl_3_): δ 0.68 (t, *J* = 7.6 Hz, 3H, CH_3_), 1.06 (sext, *J* = 7.6 Hz, 2H, CH_2_), 1.43 (quin, *J* = 7.6 Hz, 2H, CH_2_), 4.17 (t, *J* = 7.6 Hz, 2H, CH_2_), 7.06 (t, *J* = 7.2 Hz, 4H, ArH), 7.14–7.18 (m, 12H, ArH), 7.29 (t, *J* = 7.2 Hz, 8H, ArH), 7.54 (d, *J* = 8.4 Hz, 4H, ArH), 7.71–7.75 (m, 8H, ArH); ^13^C-NMR (100 MHz, CDCl_3_): δ 13.2, 19.3, 32.0, 44.8, 123.2, 123.6, 124.6, 126.1, 126.9, 127.7, 129.3, 129.4, 133.5, 142.1, 147.5, 147.9, 155.4; HRMS *m/z* calcd for (C_54_H_45_N_5_ + H^+^): 764.3753; found: 764.3749.

4-Butyl-3,5-bis[4-(naphthalen-1-yl)phenyl]-4*H*-1,2,4-triazole (**9b**). Grey solid in 62% yield, 0.165 g, m.p. 217–218 °C; UV (CH_2_Cl_2_) λ_max_ 296.0 nm (ε⋅10^−3^ 40.6 cm^−1^M^−1^); IR (ATR) ν: 3058, 2928, 2869, 1472, 1429, 1393, 1336, 1183, 1020, 965, 952, 858, 845, 831, 799, 791, 774, 752, 724 cm^−1^; ^1^H-NMR (400 MHz, CDCl_3_): δ 0.77 (t, *J* = 7.6 Hz, 3H, CH_3_), 1.17 (sext, *J* = 7.6 Hz, 2H, CH_2_), 1.57 (quin, *J* = 7.6 Hz, 2H, CH_2_), 4.30 (t, *J* = 7.6 Hz, 2H, CH_2_), 7.47–7.59 (m, 8H, ArH), 7.69 (d, *J* = 8.4 Hz, 4H, ArH), 7.86 (d, *J* = 8.4 Hz, 4H, ArH), 7.90–7.96 (m, 6H, ArH); ^13^C-NMR (100 MHz, CDCl_3_): δ 13.3, 19.4, 32.2, 44.8, 125.4, 125.7, 126.0, 126.4, 126.8, 127.0, 128.2, 128.4, 128.9, 130.7, 131.4, 133.8, 139.2, 142.7, 155.5; HRMS *m/z* calcd for (C_38_H_31_N_3_ + H^+^): 530.2596; found: 530.2596.

4-Butyl-3,5-bis[4-(naphthalen-2-yl)phenyl]-4*H*-1,2,4-triazole (**9c**). Creamy solid in 89% yield, 0.236 g, m.p. 331–334 °C; UV (CH_2_Cl_2_) λ_max_ 269.0 nm (ε⋅10^−3^ 73.8 cm^−1^M^−1^) and 305.0 (58.9); IR (ATR) ν: 3050, 2954, 2872, 1597, 1489, 1465, 1402, 1368, 1211, 1016, 972, 951, 895, 841, 813, 751, 717 cm^−1^; ^1^H-NMR (400 MHz, CDCl_3_): δ 0.72 (t, *J* = 7.6 Hz, 3H, CH_3_), 1.10 (sext, *J* = 7.6 Hz, 2H, CH_2_), 1.50 (quin, *J* = 7.6 Hz, 2H, CH_2_), 4.24 (t, *J* = 7.6 Hz, 2H, CH_2_), 7.51–7.57 (m, 4H, ArH), 7.81–7.85 (m, 6H, ArH), 7.89–7.95 (m, 8H, ArH), 7.97 (d, *J* = 8.8 Hz, 2H, ArH), 8.14 (d, *J* = 1.2 Hz, 2H, ArH); ^13^C-NMR (100 MHz, CDCl_3_): δ 13.3, 19.4, 32.1, 44.9, 125.2, 126.1, 126.3, 126.5, 126.8, 127.7, 127.9, 128.3, 128.7, 129.4, 132.9, 133.6, 137.4, 142.7, 155.4; HRMS *m/z* calcd for (C_38_H_31_N_3_ + H^+^): 530.2596; found: 530.2598.

4-Butyl-3,5-bis[4-(quinolin-3-yl)phenyl]-4*H*-1,2,4-triazole (**9d**). Grey solid in 99% yield, 0.263 g, m.p. 310–313 °C; UV (CH_2_Cl_2_) λ_max_ 270.0 nm (ε⋅10^−3^ 37.5 cm^−1^M^−1^) and 304.0 (30.4); IR (ATR) ν: 3060, 2961, 2918, 2862, 1574, 1489, 1471, 1436, 1363, 1208, 1018, 972, 951, 906, 861, 844, 833, 783, 745, 724 cm^−1^; ^1^H-NMR (400 MHz, CDCl_3_): δ 0.73 (t, *J* = 7.6 Hz, 3H, CH_3_), 1.11 (sext, *J* = 7.6 Hz, 2H, CH_2_), 1.50 (quin, *J* = 7.6 Hz, 2H, CH_2_), 4.26 (t, *J* = 7.6 Hz, 2H, CH_2_), 7.63 (t, *J* = 8.0 Hz, 2H, ArH), 7.77 (t, *J* = 8.0 Hz, 2H, ArH), 7.88–7.95 (m, 10H, ArH), 8.18 (d, *J* = 8.0 Hz, 2H, ArH), 8.41 (d, *J* = 2.4 Hz, 2H, ArH), 9.26 (d, *J* = 2.4 Hz, 2H, ArH); ^13^C-NMR (100 MHz, CDCl_3_): δ 13.3, 19.4, 32.2, 45.0, 127.3, 127.5, 127.8, 127.9, 128.1, 129.3, 129.7, 129.8, 132.7, 133.6, 139.6, 147.7, 149.5, 155.3; HRMS *m/z* calcd for (C_36_H_29_N_5_ + H^+^): 532.2501; found: 532.2507.

4-Butyl-3,5-bis[4-(quinolin-6-yl)phenyl]-4*H*-1,2,4-triazole (**9e**). White solid in 91% yield, 0.242 g, m.p. 268–270 °C; UV (CH_2_Cl_2_) λ_max_ 271.0 nm (ε⋅10^−3^ 52.9 cm^−1^M^−1^) and 306.0 (39.8); IR (ATR) ν: 2954, 2872, 1593, 1571, 1481, 1351, 1127, 975, 886, 870, 827, 792, 776, 743, 716 cm^−1^; ^1^H-NMR (400 MHz, CDCl_3_): δ 0.72 (t, *J* = 7.6 Hz, 3H, CH_3_), 1.11 (sext, *J* = 7.6 Hz, 2H, CH_2_), 1.50 (quin, *J* = 7.6 Hz, 2H, CH_2_), 4.25 (t, *J* = 7.6 Hz, 2H, CH_2_), 7.47 (dd, *J* = 8.4 and 4.4 Hz, 2H, ArH), 7.86 (d, *J* = 8.4 Hz, 4H, ArH), 7.92 (d, *J* = 8.4 Hz, 4H, ArH), 8.05 (dd, *J* = 8.4 and 2.0 Hz, 2H, ArH), 8.10 (d, *J* = 2.0 Hz, 2H, ArH), 8.22–8.27 (m, 4H, ArH), 8.96 (dd, *J* = 4.4 and 2.0 Hz, 2H, ArH); ^13^C-NMR (100 MHz, CDCl_3_): δ 13.3, 19.4, 32.1, 44.9, 121.7, 125.8, 127.2, 127.9, 128.5, 128.9, 129.5, 130.2, 136.3, 138.1, 141.9, 147.9, 150.8, 155.4; HRMS *m/z* calcd for (C_36_H_29_N_5_ + H^+^): 532.2501; found: 532.2504.

3,5-Bis[4-(dibenzothiophen-4-yl)phenyl]-4-butyl-4*H*-1,2,4-triazole (**9f**). Creamy solid in 93% yield, 0.299 g, m.p. 256–258 °C; UV (CH_2_Cl_2_) λ_max_ 241.0 nm (ε⋅10^−3^ 40.5 cm^−1^M^−1^) and 290.0 (22.4); IR (ATR) ν: 3056, 2959, 1472, 1441, 1377, 1249, 1101, 1048, 1020, 973, 846, 826, 804, 796, 755, 742, 727, 704 cm^−1^; ^1^H-NMR (400 MHz, CDCl_3_): δ 0.76 (t, *J* = 7.6 Hz, 3H, CH_3_), 1.16 (sext, *J* = 7.6 Hz, 2H, CH_2_), 1.55 (quin, *J* = 7.6 Hz, 2H, CH_2_), 4.29 (t, *J* = 7.6 Hz, 2H, CH_2_), 7.48–7.51 (m, 4H, ArH), 7.56 (d, *J* = 7.6 Hz, 2H, ArH), 7.61 (t, *J* = 7.6 Hz, 2H, ArH), 7.86 (d, *J* = 7.6 Hz, 2H, ArH), 7.89 (d, *J* = 8.4 Hz, 4H, ArH), 7.95 (d, *J* = 8.4 Hz, 4H, ArH), 8.20–8.23 (m, 4H, ArH); ^13^C-NMR (100 MHz, CDCl_3_): δ 13.3, 19.4, 32.2, 44.9, 121.0, 121.8, 122.7, 124.5, 125.2, 127.0, 127.4, 128.9, 129.4, 135.7, 135.9, 136.5, 138.5, 139.4, 142.4, 152.2, 155.4; HRMS *m/z* calcd for (C_42_H_31_N_3_S_2_ + H^+^): 642.2038; found: 642.2034.

3,5-Bis[4-(dibenzofuran-4-yl)phenyl]-4-butyl-4*H*-1,2,4-triazole (**9g**). Creamy solid in 88% yield, 0.268 g, m.p. 228–230 °C; UV (CH_2_Cl_2_) λ_max_ 286.0 nm (ε⋅10^−3^ 57.0 cm^−1^M^−1^) and 317.0 (30.6); IR (ATR) ν: 3049, 2960, 1737, 1471, 1450, 1430, 1411, 1386, 1237, 1220, 1192, 1174, 1063, 1014, 975, 854, 841, 812, 792, 772, 741 cm^−1^; ^1^H-NMR (400 MHz, CDCl_3_): δ 0.75 (t, *J* = 7.6 Hz, 3H, CH_3_), 1.15 (sext, *J* = 7.6 Hz, 2H, CH_2_), 1.55 (quin, *J* = 7.6 Hz, 2H, CH_2_), 4.30 (t, *J* = 7.6 Hz, 2H, CH_2_), 7.39 (t, *J* = 8.0 Hz, 2H, ArH), 7.46–7.53 (m, 4H, ArH), 7.65 (d, *J* = 8.0 Hz, 2H, ArH), 7.71 (d, *J* = 8.0 Hz, 2H, ArH), 7.91 (d, *J* = 8.4 Hz, 4H, ArH), 7.99–8.03 (m, 4H, ArH), 8.14 (d, *J* = 8.4 Hz, 4H, ArH); ^13^C-NMR (100 MHz, CDCl_3_): δ 13.3, 19.4, 32.2, 45.0, 111.9, 120.3, 120.8, 122.9, 123.3, 124.1, 124.7, 125.2, 126.8, 127.1, 127.4, 129.2, 129.3, 138.1, 153.4, 155.5, 156.2; HRMS *m/z* calcd for (C_42_H_31_N_3_O_2_ + H^+^): 610.2495; found: 610.2491.

4-Butyl-3,5-bis[4-(9-methyl-9*H*-carbazol-3-yl)phenyl]-4*H*-1,2,4-triazole (**9h**). Creamy solid in 97% yield, 0.308 g, m.p. 267–268 °C; UV (CH_2_Cl_2_) λ_max_ 300.0 nm (ε⋅10^−3^ 76.8 cm^−1^M^−1^) and 323.0 (51.0); IR (ATR) ν: 3047, 2957, 2934, 1597, 1483, 1466, 1424, 1360, 1335, 1323, 1294, 1254, 1221, 1154, 1121, 1012, 843, 825, 813, 799, 772, 749, 739, 721 cm^−1^; ^1^H-NMR (400 MHz, CDCl_3_): δ 0.73 (t, *J* = 7.6 Hz, 3H, CH_3_), 1.11 (sext, *J* = 7.6 Hz, 2H, CH_2_), 1.50 (quin, *J* = 7.6 Hz, 2H, CH_2_), 3.91 (s, 6H, CH_3_), 4.24 (t, *J* = 7.6 Hz, 2H, CH_2_), 7.28 (t, *J* = 7.6 Hz, 2H, ArH), 7.44 (d, *J* = 7.6 Hz, 2H, ArH), 7.49–7.54 (m, 4H, ArH), 7.80–7.82 (m, 6H, ArH), 7.90 (d, *J* = 8.4 Hz, 4H, ArH), 8.18 (d, *J* = 7.6 Hz, 2H, ArH), 8.40 (s, 2H, ArH); ^13^C-NMR (100 MHz, CDCl_3_): δ 13.3, 19.4, 29.3, 32.1, 44.9, 108.7, 108.9, 118.9, 119.2, 120.4, 122.9, 123.4, 125.1, 125.8, 126.1, 127.6, 129.3, 131.1, 140.9, 141.5, 143.7, 155.6; HRMS *m/z* calcd for (C_44_H_37_N_5_ + H^+^): 636.3127; found: 636.3123.

4-Butyl-3,5-bis[4-(9-ethyl-9*H*-carbazol-3-yl)phenyl]-4*H*-1,2,4-triazole (**9i**). Creamy solid in 98% yield, 0.326 g, m.p. 269–270 °C; UV (CH_2_Cl_2_) λ_max_ 243.0 nm (ε⋅10^−3^ 51.1 cm^−1^M^−1^), 301.0 (70.7) and 323.0 (47.1); IR (ATR) ν: 3049, 2973, 1740, 1598, 1460, 1379, 1346, 1325, 1295, 1252, 1233, 1197, 1154, 1125, 1087, 848, 804, 769, 747, 730, 694 cm^−1^; ^1^H-NMR (400 MHz, CDCl_3_): δ 0.72 (t, *J* = 7.6 Hz, 3H, CH_3_), 1.11 (sext, *J* = 7.6 Hz, 2H, CH_2_), 1.46–1.53 (m, 8H, CH_2_ + CH_3_), 4.24 (t, *J* = 7.6 Hz, 2H, CH_2_), 4.42 (q, *J* = 7.2 Hz, 4H, CH_2_), 7.28 (t, *J* = 7.6 Hz, 2H, ArH), 7.45 (d, *J* = 7.6 Hz, 2H, ArH), 7.49–7.53 (m, 4H, ArH), 7.78–7.82 (m, 6H, ArH), 7.90 (d, *J* = 8.4 Hz, 4H, ArH), 8.18 (d, *J* = 7.6 Hz, 2H, ArH), 8.40 (s, 2H, ArH); ^13^C-NMR (100 MHz, CDCl_3_): δ 13.3, 13.8, 19.4, 32.1, 37.7, 44.9, 108.7, 108.8, 119.0, 119.1, 120.5, 123.0, 123.6, 125.1, 125.8, 126.0, 127.6, 129.3, 131.0, 139.8, 140.4, 143.7, 155.6; HRMS *m/z* calcd for (C_46_H_41_N_5_ + H^+^): 664.3440; found: 664.3439.

4-Butyl-3,5-bis[4′-(9*H*-carbazol-9-yl)biphenyl-4-yl]-4*H*-1,2,4-triazole (**9j**). Grey solid in 91% yield, 0.344 g, m.p. 320–323 °C; UV (CH_2_Cl_2_) λ_max_ 293.0 nm (ε⋅10^−3^ 50.5 cm^−1^M^−1^) and 320.0 (42.6); IR (ATR) ν: 3054, 2954, 1599, 1521, 1490, 1478, 1451, 1413, 1367, 1334, 1317, 1303, 1226, 1185, 1171, 1004, 974, 826, 771, 743, 719 cm^−1^; ^1^H-NMR (400 MHz, CDCl_3_): δ 0.74 (t, *J* = 7.6 Hz, 3H, CH_3_), 1.13 (sext, *J* = 7.6 Hz, 2H, CH_2_), 1.52 (quin, *J* = 7.6 Hz, 2H, CH_2_), 4.26 (t, *J* = 7.6 Hz, 2H, CH_2_), 7.32 (t, *J* = 7.6 Hz, 4H, ArH), 7.45 (t, *J* = 7.6 Hz, 4H, ArH), 7.50 (d, *J* = 7.6 Hz, 4H, ArH), 7.71 (d, *J* = 8.4 Hz, 4H, ArH), 7.85–7.89 (m, 8H, ArH), 7.92 (d, *J* = 8.4 Hz, 4H, ArH), 8.18 (d, *J* = 7.6 Hz, 4H, ArH); ^13^C-NMR (100 MHz, CDCl_3_): δ 13.3, 19.4, 32.1, 44.9, 109.8, 120.1, 120.4, 123.5, 126.0, 127.0, 127.5, 127.6, 128.6, 129.5, 137.6, 139.0, 140.8, 141.9, 155.4; HRMS *m/z* calcd for (C_54_H_41_N_5_ + H^+^): 760.3440; found: 760.3438.

4-Butyl-3,5-bis[4-(thiantren-1-yl)phenyl]-4*H*-1,2,4-triazole (**9k**). Creamy solid in 86% yield, 0.305 g, m.p. 264–266 °C; UV (CH_2_Cl_2_) λ_max_ 263.0 nm (ε⋅10^−3^ 66.7 cm^−1^M^−1^); IR (ATR) ν: 3048, 2952, 2870, 1474, 1441, 1397, 1247, 1188, 1109, 1018, 973, 843, 807, 788, 748, 728, 686 cm^−1^; ^1^H-NMR (400 MHz, CDCl_3_): δ 0.78 (t, *J* = 7.6 Hz, 3H, CH_3_), 1.20 (sext, *J* = 7.6 Hz, 2H, CH_2_), 1.57 (quin, *J* = 7.6 Hz, 2H, CH_2_), 4.29 (t, *J* = 7.6 Hz, 2H, CH_2_), 7.21 (t, *J* = 7.6 Hz, 2H, ArH), 7.26 (t, *J* = 7.6 Hz, 2H, ArH), 7.31–7.34 (m, 4H, ArH), 7.37 (d, *J* = 7.6 Hz, 2H, ArH), 7.52 (d, *J* = 7.6 Hz, 2H, ArH), 7.57 (d, *J* = 7.6 Hz, 2H, ArH), 7.60 (d, *J* = 8.4 Hz, 4H, ArH), 7.83 (d, *J* = 8.4 Hz, 4H, ArH); ^13^C-NMR (100 MHz, CDCl_3_): δ 13.3, 19.3, 32.1, 44.8, 127.2, 127.7, 127.9, 128.3, 128.6, 128.7, 128.8, 128.9, 129.1, 130.1, 135.1, 135.7, 135.9, 136.2, 141.5, 141.9, 155.4; HRMS *m/z* calcd for (C_42_H_31_N_3_S_4_ + H^+^): 706.1479; found: 706.1474.

4-Hexyl-3,5-bis[4′-(*N,N*-diphenylamino)biphenyl-4-yl]-4*H*-1,2,4-triazole (**10a**). Yellow solid in 61% yield, 0.240 g, m.p. 244–246 °C; UV (CH_2_Cl_2_) λ_max_ 352.0 nm (ε⋅10^−3^ 68.0 cm^−1^M^−1^); IR (ATR) ν: 3033, 2925, 2855, 1586, 1508, 1484, 1411, 1328, 1278, 1203, 1173, 1121, 1076, 1025, 1005, 968, 822, 748, 729, 691 cm^−1^; ^1^H-NMR (400 MHz, CDCl_3_): δ 0.73 (t, *J* = 7.2 Hz, 3H, CH_3_), 0.99–1.02 (m, 4H, 2 × CH_2_), 1.08 (quin, *J* = 7.2 Hz, 2H, CH_2_), 1.44 (quin, *J* = 7.2 Hz, 2H, CH_2_), 4.16 (t, *J* = 7.2 Hz, 2H, CH_2_), 7.06 (t, *J* = 7.2 Hz, 4H, ArH), 7.13–7.18 (m, 12H, ArH), 7.29 (t, *J* = 7.2 Hz, 8H, ArH), 7.55 (d, *J* = 8.4 Hz, 4H, ArH), 7.71–7.75 (m, 8H, ArH); ^13^C-NMR (100 MHz, CDCl_3_): δ 13.8, 22.2, 25.7, 29.9, 30.7, 45.0, 123.2, 123.6, 124.6, 126.1, 126.9, 127.8, 129.3, 129.4, 133.5, 142.1, 147.5, 147.9, 155.5; HRMS *m/z* calcd for (C_56_H_49_N_5_ + H^+^): 792.4066; found: 792.4061.

## 3. Results and Discussion

**Scheme 2:**
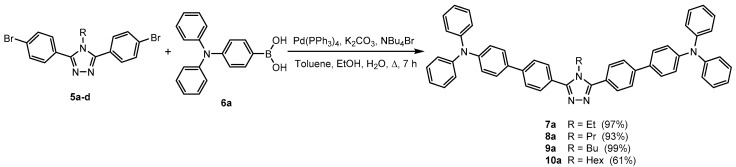
The conventional Suzuki cross-coupling reaction for 4-alkyl-3,5-bis(4-bromophenyl)-4*H*-1,2,4-triazole derivatives. Reagents and conditions: aryl dibromide **5a–d** (1.00 mmol), 4-(*N*,*N*-diphenylamino)phenylboronic acid (**6a**) (2.50 mmol), Pd(PPh_3_)_4_ (0.05 mmol), NBu_4_Br (0.10 mmol), K_2_CO_3_ (10 mmol), toluene/H_2_O/EtOH (10:6:3 mL), oil bath 130 °C, 7 h.

Four basic 4-alkyl-3,5-bis(4-bromophenyl)-4*H*-1,2,4-triazole (**5a–d**) substrates were synthesized starting from 4-bromobenzoic acid (**1**), using our previously elaborated methodologies [58] (Scheme 1). For each reaction, triazole precursors (**5a–d**) containing substituents, differing in their alkyl chain length at the nitrogen atom (position 4), were coupled with 4-(*N*,*N*-diphenylamino)phenylboronic acid (**6a**) in a Suzuki reaction. Transformations were completed using a two-phase solvent system with conventional heating in an oil bath over a sufficiently long period (7 h, TLC) using Pd(PPh_3_)_4_ as a catalyst, and potassium carbonate as a base which gives the desired products in high yields (Scheme 2).

**Figure 1:**
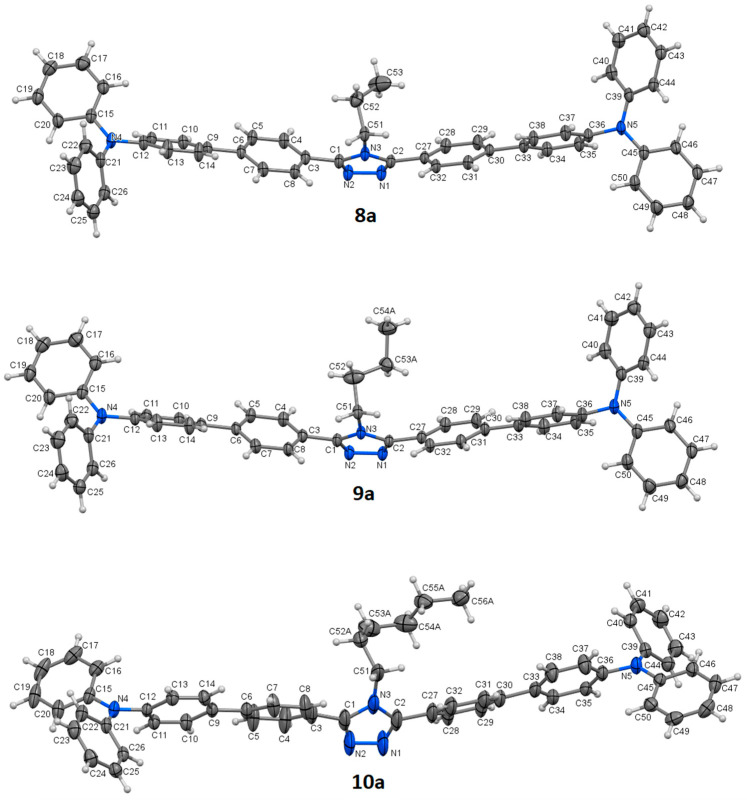
The molecular structures of compounds **8a**–**10a**, with an atom numbering scheme, plotted with a 50% probability of the displacement ellipsoids of non-hydrogen atoms. Hydrogen atoms are plotted as spheres with arbitrary radii. The disordered solvent molecules of **8a** and **9a**, as well as a minor contribution from the distorted alkyl chains of **9a** and **10a**, are omitted for clarity.

Three (**8a**–**10a**) of the final products form single crystals in the solid-state (Figure 1), enabling the determination of their molecular structures (Table S1). Compounds **8a** and **9a** crystallize as ethanol solvates (Figure S1), while **10a** forms a solventless compound. This is caused by the presence of an *n*-hexyl substituent on the triazole ring of **10a**. The *n*-hexyl terminal methyl and methylene atoms (-CH_2_–CH_3_) are located in the space occupied by ethanol molecules in **8a** and **9a** (Figures S2–S4). The shorter *n*-butyl and *n*-propyl substituents cannot fill the crystal net space and leave empty voids with volumes of 153–216 Å^3^, which are accessible for solvent molecules. Due to discrepancy between the volume of the solvent molecules (~97 Å^3^ for one ethanol molecule) and voids, these molecules are disordered in the crystal net (Figures S1 and S5). This affects the neighboring parts of alkyl substituents, which are also disordered in **9a** and **10a** due to the relative freedom of their packing (Figure S1). Compounds **8a** and **9a** in the solid-state are effectively isostructural, whilst **10a** adopts a different packing pattern. In all compounds (**8a–10a**), the molecules extend in one direction of the crystal net, but their mutual shifts are different. In **8a** and **9a,** the subsequent molecules are shifted to approximately half of the preceding molecule length, i.e., the terminal *N*,*N*-diphenylamino group of one molecule is located near the central triazole ring of the neighboring molecule (Figures S2 and S3). In **10a,** the terminal *N*,*N*-diphenylamino groups of the neighboring molecules are adjacent (Figure S4). The overall conformation of molecules **8a–10a** is similar, such that the molecules extend along axes going through the N(triazole)–C(phenylene) bonds due to the presence of 1,4-phenylene moieties. The mutual arrangement of the ring groups is different in all compounds (**8a–10a**), including clearly visible differences in the isostructural **8a** and **9a**. The dihedral angles between the least-squares plane of the diphenylene moiety rings vary from 12.3(1)° to 31.95(6)°, while the angles between the *N*,*N*-diphenylamino moiety rings differ from 52.80(5)° to 66.52(6)° (Table S3). The C–N and N–N bonds with the triazole ring possess similar lengths (Table S2) and respective values are between the length of single and double bonds C–N and N–N, [59] what proves the electron delocalization within the ring. The C(triazole)-C(phenylene) bonds exhibit shortening observed typically in exocyclic bonds connected to aromatic rings [59], which results in the redirecting of the electron density toward the C–C bond. The complete hydrogen bonding scheme of compounds **8a** and **9a** could not be resolved due to the disorder in the solvent molecules’ positions. Nevertheless, the studied molecules possess only two classical hydrogen bonds acceptors and non-classical hydrogen bonds donors [60]. The N1 and N2 atoms participate in weak C–H···N hydrogen bonds (Table S4). The isostructural forms of **8a** and **9a** have the same π···π stacking interaction motifs due to the similarity of their crystal packing [61]. One of the phenyl rings from each terminal *N*,*N*-diphenylamino substituent interacts with their symmetry generated equivalent rings. Additionally, one of these rings interacts with the symmetry generated ring of the *N*,*N*-diphenylamino moiety located on the opposite end of the molecule (Table S5). Consequently, one of the four rings belonging to *N*,*N*-diphenylamino substituents interacts with two rings, meaning two interact with one ring, and one does not form any π···π stacking interactions. In **10a,** only two different π···π stacking interactions exist. The first one is formed between the neighboring triazole rings and the second one between one at the phenyl rings of each terminal *N*,*N*-diphenylamino moiety, i.e., similarly to **8a** and **9a**, the phenyl ring of one terminal moiety interacts with the symmetry generated ring of the oppositely located terminal moiety (Table S5).

**Figure 2:**
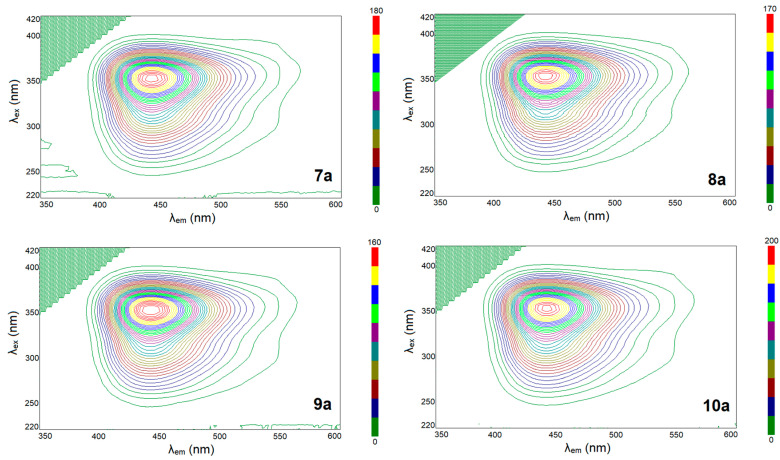
Three-dimensional fluorescence spectra of compounds **7a**–**10a**. The color scale represents the flux of emitted photons. The number above the color scales indicates the maximum relative value of emission intensity represented by color scale (and indicated in the respective figure). The unit of measurement in each spectrum represents the same number of emitted photons per second, i.e., all values of intensity presented in Figure 2, Figure 3 and Figure 4 can be directly compared. The green area (upper left triangles in each spectrum) indicates the non-fluorescent region in which *λ*_ex_ is close to or larger than *λ*_em_.

The final products were tested for their luminescent properties, displaying strong fluorescent properties (Table 1, Figure 2). The compounds have a relatively large Stokes shift (Δ*λ* = ~90 nm) and a near-unity quantum yield (Φ > 0.98). From these results, an alternative approach to the Suzuki reaction was investigated (Table 2).

The use of ionic liquids in the Suzuki cross-coupling reaction opens up the possibility for the additional acceleration of the reaction with the use of ultrasound or microwaves. These variants (Table 2, entries: 3–6, 9–12, 15–18 and 21–24) have a shorter reaction time compared to conventional heating (Table 2, entries: 1, 2, 7, 8, 13, 14, 19 and 20) in an oil bath while maintaining a high coupling efficiency. The most beneficial aspects from an economic point of view is the use of a small addition of a classical solvent (toluene, 1 mL) and conducting the reaction in a microwave reactor, where the complete conversion of substrates takes place after only 6 min (TLC), and the products are obtained in high yields (Table 2, entries: 6, 12, 18 and 24, 62–97%).

**Scheme 3:**
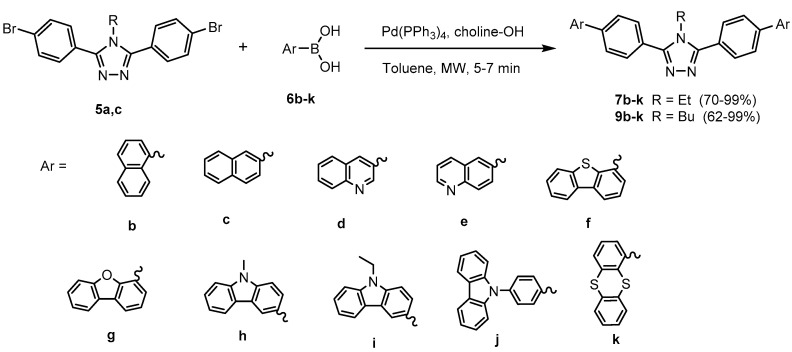
The Suzuki coupling reaction of 4*H*-1,2,4-triazole precursors **5a** and **5c** with selected boronic acids **6b–k**. Reagents and conditions: aryl dibromide **5a,c** (1.00 mmol), arylboronic acid **6b–k** (2.50 mmol), Pd(PPh_3_)_4_ (0.05 mmol), choline–OH/toluene (10:1 mL), and microwave irradiation, 5–7 min.

The highest yields were obtained for derivatives with two and four carbon alkyl chains, the subsequent reactions were carried out for these two series of triazole precursors, **5a** and **5c** (Scheme 3), and then their emission properties were tested (Table 3). Generally, compounds containing a 4*H*-1,2,4-triazole core substituted with an ethyl group at the position 4 were synthesized with a slightly smaller range of yields (70–99% for **7a**–**k**, Table 1 and Table 3) than their counterparts with the butyl substituent (62–99% for **9a**–**k**, Table 1 and Table 3).

**Figure 3:**
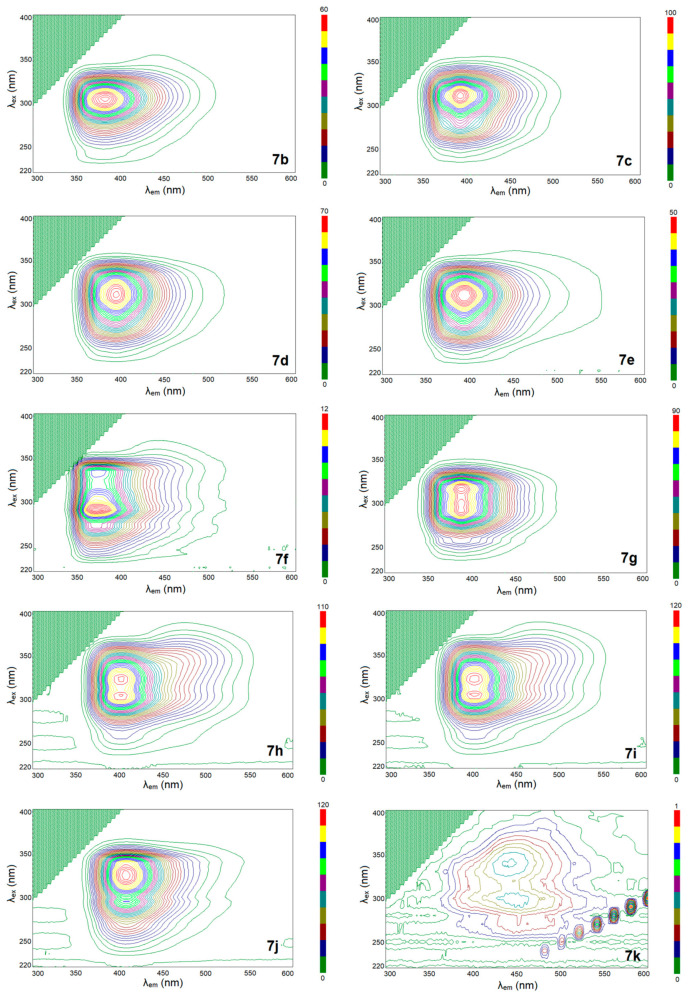
Three-dimensional fluorescence spectra of compounds **7b**–**7k**. The color scale represents the flux of emitted photons. The number above the color scales indicates the maximum relative value of emission intensity represented by color scale (and indicated in the respective figure). The unit of measurement in each spectrum represents the same number of emitted photons per second, i.e., all values of intensity presented in Figure 2, Figure 3 and Figure 4 can be directly compared. The green area (upper left triangles in each spectrum) indicates the non-fluorescent region in which *λ*_ex_ is close to or larger than *λ*_em_.

**Figure 4:**
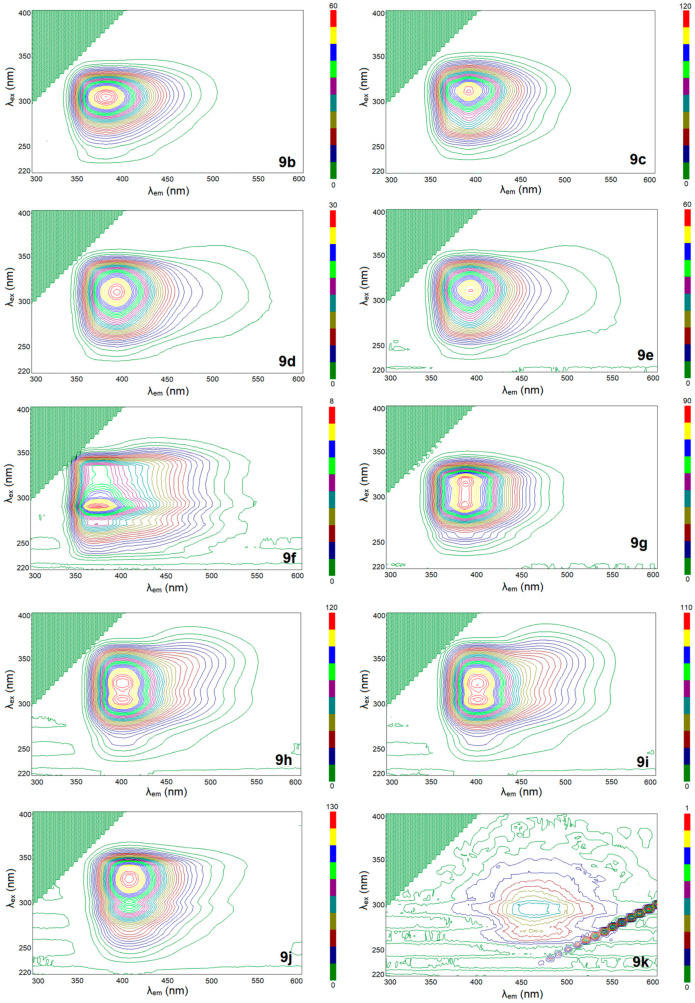
Three-dimensional fluorescence spectra of compounds **9b**–**9k**. The color scale represents the flux of emitted photons. The number above the color scales indicates the maximum relative value of emission intensity represented by color scale (and indicated in the respective figure). The unit of measurement in each spectrum represents the same number of emitted photons per second, i.e., all values of intensity presented in Figure 2, Figure 3 and Figure 4 can be directly compared. The green area (upper left triangles in each spectrum) indicates the non-fluorescent region in which *λ*_ex_ is close to or larger than *λ*_em_.

Three-dimensional fluorescence characteristics were determined for all the final products from the outlined reactions. The spectra of compounds **7a–7e**, **8a**, **9a–9e** and **10a** possess a single fluorescence maximum, while compounds **7f–7k** and **9f–9k** have two maxima (Figure 2, Figure 3 and Figure 4). Almost all of the synthesized compounds emit strong fluorescence upon irradiation with UV light. The only exceptions are the compounds **7k** and **9k** containing the thianthrene substituent (Table 3). This is due to the presence of two sulfur atoms in the thianthrene moiety. Typically, the presence of one sulfur atom in a molecule causes the severe quenching of fluorescence [65,66]. In each molecule of compound **7k** and **9k,** four sulfur atoms are present, thus, the almost total quenching of fluorescence is observed (Figure 3 and Figure 4). Similarly, the existence of two sulfur atoms per one molecule in **7f** and **9f** distinctly decreases the number of emitted photons in comparison to the other studied compounds without a sulfur atom in the structure. The change in the aliphatic substituent length at the triazole nitrogen N atom does not affect the position and shape of the three-dimensional emission maxima. The effect of increased emission wavelengths for aliphatic substituents with even number of carbon atoms (in comparison to these with odd number of carbon atoms) is not observed in the studied case [58]. This suggests that the presence of substituents containing a single ring at the ends of the 4-alkyl-3,5-bis(phenyl)-4*H*-1,2,4-triazole core is necessary for the aforementioned even/odd number of carbon atoms and its influence on fluorescence. In the studied compounds, the terminal substituents consists of two up to four rings (in the subsequent or fused arrangement) and this completely diminishes the effect of the aliphatic substituent parity at the triazole nitrogen N atom.

The number of rings in terminal substituents also affects the excitation and emission wavelengths (Figure S8). There is a decrease in the excitation wavelength for the first maximum on the spectrum (accompanied by an increase in the excitation wavelength for the second maximum on the spectrum) with an increase in the number of rings (Figure S8). The difference between the excitation and emission wavelengths at the maxima falls by approximately 70–84 nm (for compounds possessing two fluorescence maxima, at least one fulfills this relation), thus the energy gap between the respective orbitals responsible for the fluorescence is very similar in all studied compounds. The spectra of the compounds with rings separated by a single bond (**7a–10a**) or with no more than two fused rings (**7b–7e** and **9b–9e**) possess a single maximum in the three-dimensional fluorescence spectrum. The compounds with three fused rings (**7f–7k** and **9f–9k**) possess two more or less shaped maxima at the same emission wavelength and two different excitation wavelengths. Manifestations of these maxima were faintly but unambiguously visible in the compounds with almost completely quenched fluorescence (**7k** and **9k**). All the single maxima possess a considerable degree of asymmetry, with the emission drastically fading with a decrease in the emission wavelengths and/or increase in the excitation wavelengths. In two other directions of the spectra, the emission decreases slowly. This originates from the electronic transitions of the excited state (S_1_) to the different energy sub-levels (vibrational energy levels 0, 1, 2, …) of the ground state (S_0_). The asymmetricity increases with the number of electrons in the delocalized system (**7b** ≈ **7c** ≈ **9b** ≈ **9c** < **7d** ≈ **7e** ≈ **9d** ≈ **9e** < **7a** ≈ **8a** ≈ **9a** ≈ **10a**), due to the increased number of vibrational energy levels. The presence of two fluorescence maxima in the spectra of compounds **7f–7k** and **9f–9k** is caused by the presence of ring systems possessing two different heteroatomic rings (central and terminal ones). The intensity of both maxima is similar for compounds **7g–7j** and **9g–9j**. For compounds **7f** and **9f,** the first (at lower excitation wavelengths) maxima is stronger than the second one (existing at larger excitation wavelengths), while for compounds **7j** and **9j** the mutual relation of maxima intensities is reversed. This effect is caused by the presence of a thiophene moiety in the thianthrene ring system of **7f** and **9f** and an occurrence of an additional phenylene moiety in **7j** and **9j** (in comparison to the other studied compounds). The conjugation of the triazole ring with appropriately selected substituents containing together more than six aromatic rings (**7a–10a**, **7g–7j**, and **9g–9j** excluding **7f**, **7k**, **9f**, and **9k,** containing sulfur atoms) leads to reach a nearly 1.00 value of quantum yield (Φ). Such properties are not achieved by triazoles conjugated with four aromatic rings [58]. Their Φ, although high, reaches values maximally around 0.9. The triazoles which are not bonded directly to aryl substituents are characterized by a very low Φ (below 0.01) [67]. To improve the fluorescence properties of triazoles conjugated with a lower number of aromatic rings, the coordination compounds with *d*^10^ transition metals are synthesized [68]. Even then, the zinc compounds of triazoles with one aromatic ring achieve Φ only up to 0.075 [69]. The Φ of the studied compounds correlate well with the fluorescence intensities (larger fluorescence leads to larger Φ, Figure S9), whereas there is no discernible relationship between Φ and absorption (Figure S10). This suggests that the mechanism of fluorescence is similar in all compounds and the relatively large differences in the absorption values derive from the variations in the amount of electromagnetic energy (photons) transformed directly into internal energy. The above described effects (presence/lack of a correlation) exist also in 4-alkyl-3,5-bis(phenyl)-4*H*-1,2,4-triazole substituted at the ends by a single ring [58] and there is no such effects in the di(1,3,4-oxadiazol-2-yl)-1,2,4,5-tetrazine and 3,6-di(1,3,4-thiadiazol-2-yl)-1,2,4,5-tetrazine derivatives [70]. In the case of the compounds exhibiting a single fluorescence maximum, the n→π* absorption transition is the main source of the excited states leading to the subsequent emission of the fluorescence photons. The origin of fluorescence in the compounds exhibiting two fluorescence maxima are n→π* (for higher $\lambda_{max}^{ex}$) and *π*→π* (for lower $\lambda_{max}^{ex}$) absorption transitions [68]. The involved n-type orbital originates from the nitrogen atom of the triazole ring (N1 or N2) and the source of the *π*-type orbital is also the triazole ring [71]. The lowest unoccupied molecular orbital in similarly conjugated 1,2,4-triazole compounds is antibonding delocalized orbital (π*), which most often is the benzene ring directly attached to the triazole ring [72] or fused π* orbital of triazole and the neighboring benzene [73]. The presence of a single fluorescence maximum or two fluorescence maxima of the same emission wavelength indicates that π*→*π* transitions are responsible for the fluorescence in the studied compounds [74]. Noteworthy is the fact that upon ultraviolet irradiation, the compounds **7a–10a** emit a strong deep-blue fluorescence light visible by the naked eye. The blue-violet color emitted light is clearly visible for compound **7c–7e, 7h–7j, 9c–9e, 9h–9j**.

## 4. Conclusions

Four alternative approaches using palladium-catalyzed Suzuki cross-coupling reactions to synthesize a series of highly-conjugated 4*H*-1,2,4-triazole derivatives were presented. The reactions of the intermediate 4-alkyl-3,5-bis(4-bromophenyl)-4*H*-1,2,4-triazoles and boronic acids were conducted using conventional heating in a two-phase solvent system (toluene/EtOH/H_2_O) or using an ionic liquid (choline–OH). For the ionic liquid approach, it was possible to use ultrasound or microwave assistance, which significantly reduces the reaction time. Additionally, this type of methodology removes the need for large amounts of organic solvents and for the addition of auxiliary reagents such as a base or a phase transfer catalyst. Generally, 4-alkyl-4*H*-1,2,4-triazole derivatives conjugated to different fused-bicyclic and fused-tricyclic systems via a 1,4-phenylene linker were obtained in excellent yields. Almost all compounds are effective fluorophores and emit visible light upon irradiation by ultraviolet electromagnetic waves. The exceptions are the products possessing sulfur atoms within terminal moieties, which cause a large decrease in the fluorescence. The studied compounds produce fluorescence with large quantum yields (up to almost 100%), which depend on a structure of the terminal substituents. The obtained compounds, due to their good emission properties and relatively good solubility in typical organic solvents, seem to be of particular interest for optoelectronic purposes.

## Figures and Tables

**Table 1:** 4-Alkyl-3,5-bis[4′-(*N*,*N*-diphenylamino)biphenyl-4-yl]-4*H*-1,2,4-triazoles (**7–10a**) prepared using the Suzuki cross-coupling reaction ^a.^

Product	Yield ^a^ (%)	$\mathrm{Absorption}\;\mathrm{Maximum}\;\lambda_{max}^{abs}\;(\mathrm{nm})$	$\mathrm{Excitation}\;\mathrm{Wavelength}\;\lambda_{max}^{ex}\;(\mathrm{nm})$	$\mathrm{Emission}\;\mathrm{Wavelength}\;\lambda_{max}^{em}\;(\mathrm{nm})$	Stokes Shift ^b^ Δ (nm)	Quantum Yield ^c,d,e^ Φ
**7a**	97	351	352	443	92	>0.98 ^c,d,e^
**8a**	93	352	352	442	90	>0.98 ^c,d,e^
**9a**	99	351	351	443	92	>0.98 ^c,d,e^
**10a**	61	352	352	443	91	>0.98 ^c,d,e^

^a^ isolated yield. ^b^ stokes shift (Δ) from the equation $\Delta \;=\;\lambda_{max}^{em}-\lambda_{max}^{abs}$. Wavelength determined from the 3D emission spectrum for C = 5.0 × 10^−6^ M CH_2_Cl_2_ solution (nm). ^c^ quinine sulfate was used as a standard [62]. ^d^ trans,trans-1,4-diphenyl-1,3-butadiene was used as a standard [63]. ^e^ exact value cannot be determined due to nonlinearity of standard/sample dependence in the Φ region of 0.97–1.00 [64].

**Table 2:** An alternative ionic liquids (IL) Suzuki cross-coupling reaction of the 4-alkyl-3,5-bis(4-bromophenyl)-4*H*-1,2,4-triazole moiety (**7a–10a**).

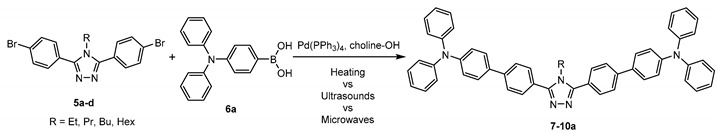					
Entry	Substrate	Approach	Reaction Time	Additional Solvent (1 mL)	Yield ^a^ (%)
1	**5a**	Conventional heating (oil bath 130 °C)	24 h	-	-
2				Toluene	85
3		Ultrasounds	10 h	-	5
4				Toluene	89
5		Microwaves	6 min	-	8
6				Toluene	94
7	**5b**	Conventional heating (oil bath 130 °C)	24 h	-	-
8				Toluene	80
9		Ultrasounds	10 h	-	3
10				Toluene	87
11		Microwaves	6 min	-	10
12				Toluene	89
13	**5c**	Conventional heating (oil bath 130 °C)	24 h	-	-
14				Toluene	85
15		Ultrasounds	10 h	-	3
16				Toluene	93
17		Microwaves	6 min	-	9
18				Toluene	97
19	**5d**	Conventional heating (oil bath 130 °C)	24 vh	-	-
20				Toluene	47
21		Ultrasounds	10 h	-	-
22				Toluene	56
23		Microwaves	6 min	-	5
24				Toluene	62

^a^ yield with respect to the 4-alkyl-3,5-bis(4-bromophenyl)-4*H*-1,2,4-triazole (**5a–d**). Reagents and conditions: aryl dibromide **5a–d** (1.00 mmol), boronic acid **6a** (2.50 mmol), Pd(PPh_3_)_4_ (0.05 mmol), choline–OH (10 mL).

**Table 3:** 4-Alkyl-3,5-bis(4-arylphenyl)-4*H*-1,2,4-triazoles **7b–k** and **9b–k** prepared using the Suzuki cross-coupling reaction ^a^.

Product	Yield ^a^ (%)	$\mathrm{Absorption}\;\mathrm{Maximum}\;\lambda_{max}^{abs}\;(\mathrm{nm})$	$\mathrm{Excitation}\;\mathrm{Wavelength}\;\lambda_{max}^{ex}\;(\mathrm{nm})$	$\mathrm{Emission}\;\mathrm{Wavelength}\;\lambda_{max}^{em}\;(\mathrm{nm})$	Stokes Shift ^b^ Δ (nm)	Quantum Yield ^c,d,e^ Φ
**7b**	88	297	304	382	85	0.75 ^c^/0.74 ^d^
**7c**	99	305	313	394	89	>0.98 ^c,d,e^
**7d**	79	305	312	395	90	0.75 ^c^/0.74 ^d^
**7e**	94	306	312	394	88	0.55 ^c^/0.54 ^d^
**7f**	96	290	291	371	81	0.11 ^c^/0.11 ^d^
		335	335	374	39	0.18 ^c^/0.18 ^d^
**7g**	92	286	290	388	102	0.63 ^c^/0.62 ^d^
		315	316	388	73	0.95 ^c^/0.93 ^d^
**7h**	70	300	304	403	103	0.77 ^c^/0.76 ^d^
		321	322	401	80	>0.98 ^c,d,e^
**7i**	76	301	304	404	103	0.78 ^c^/0.77 ^d^
		322	322	403	81	>0.98 ^c,d,e^
**7j**	77	293	293	409	116	0.77 ^c^/0.76 ^d^
		322	328	408	86	>0.98 ^c,d,e^
**7k**	87	263	-	-	-	-
**9b**	62	296	304	382	86	0.64 ^c^/0.62 ^d^
**9c**	89	305	313	393	88	0.95 ^c^/0.93 ^d^
**9d**	99	304	311	395	91	0.40 ^c^/0.39 ^d^
**9e**	91	306	313	393	87	0.60 ^c^/0.58 ^d^
**9f**	93	290	291	373	83	0.14 ^c^/0.14 ^d^
		335	335	376	41	0.27 ^c^/0.26 ^d^
**9g**	88	286	292	387	101	0.76 ^c^/0.75 ^d^
		317	318	388	71	>0.98 ^c,d,e^
**9h**	97	300	304	402	102	0.80 ^c^/0.78 ^d^
		323	323	402	79	>0.98 ^c,d,e^
**9i**	98	301	304	403	102	0.78 ^c^/0.77 ^d^
		323	323	402	79	>0.98 ^c,d,e^
**9j**	91	293	295	409	116	0.81 ^c^/0.79 ^d^
		320	327	408	88	>0.98 ^c,d,e^
**9k**	86	263	-	-	-	-

^a^ isolated yield. ^b^ stokes shift (Δ) from the equation $\Delta \;=\;\lambda_{max}^{em}-\lambda_{max}^{abs}$. Wavelength determined from the 3D emission spectrum for C = 5.0 × 10^−6^ M CH_2_Cl_2_ solution (nm). ^c^ quinine sulfate was used as a standard [62]. ^d^ trans,trans-1,4-diphenyl-1,3-butadiene was used as a standard [63]. ^e^ exact value cannot be determined due to the nonlinearity of standard/sample dependence in the Φ region 0.97–1.00 [64].

## Supplementary Materials

The following are available online at https://www.mdpi.com/1996-1944/13/24/5627/s1, copy of ^1^H NMR, ^13^C NMR and HRMS spectra; X-ray crystallography data; absorption spectrometry data. Figure S1: Complete asymmetric units of the structures of compounds **8a**, **9a**, and **10a**, with atom numbering scheme, plotted with 50% probability of displacement ellipsoids of non-hydrogen atoms. Hydrogen atoms are plotted as spheres of arbitrary radii, Figure S2: Solvent accessible voids within crystal structure of **8a**, Figure S3: Solvent accessible voids within crystal structure of **9a**, Figure S4: The part of crystal packing in **10a**, Figure S5: Disorder model of solvent in **8a**, Figure S6: UV-Vis spectra of **7a–10a** and **7b–7k**, Figure S7: UV-Vis spectra of **9b–9k**, Figure S8: Positions of global maxima for studied compounds (divided into groups containing 2 or 3 rings at the ends of 4-alkyl-3,5-bis(phenyl)-4*H*-1,2,4-triazole core). “1^st^ max.” and “2^nd^ max.” indicate the first (for smaller excitation wavelengths) and the second (for larger excitation wavelengths) maxima on three-dimensional fluorescence spectra, Figure S9: Quantum yield of studied compounds as a function of fluorescence intensity at global and local maximum, Figure S10: Quantum yield of studied compounds in relation to absorption at global and local maximum of fluorescence, Table S1: Crystal data and structure refinement details for **8a**, **9a**, and **10a**. The structures of **8a** and **9a** were refined twice: against measured and squeezed data, Table S2: Selected structural data of **8a**, **9a**, and **10a**, Table S3: Dihedral angles (°) between ring least squares planes in **8a**, **9a**, and **10a**. Each ring is indicated by one atom, which belongs solely to this ring, Table S4: Non-classic hydrogen bonds and the first level graph motifs in the studied compounds (Å, °), Table S5: Stacking interactions in the studied compounds. Each ring is indicated by one atom, which belongs solely to this ring. The *α* is a dihedral angle between planes I and J, *β* is an angle between Cg(I)-Cg(J) vector and normal to plane I, d_p_ is a perpendicular distance of Cg(I) on ring J plane.
