# Supplementary material for: Highly Luminescent 4H-1,2,4-Triazole Derivatives: Synthesis, Molecular Structure and Photophysical Properties
Source: Materials (Basel). 2020 Dec 10;13(24):5627. doi: 10.3390/ma13245627 (PMC7764194; doi:10.3390/ma13245627)
Supplement: Supplementary file 1 [file materials-13-05627-s001.pdf]

Supplementary Materials

Highly luminescent 4*H*-1,2,4-triazole derivatives: synthesis, molecular structure and photophysical properties

Monika Olesiejuk[1,*], Agnieszka Kudelko[1] and Marcin Swiatkowski[2]

[1] Department of Chemical Organic Technology and Petrochemistry, The Silesian University of Technology, Krzywoustego 4, PL-44100 Gliwice, Poland; monika.olesiejuk@polsl.pl

[2] Department of X-ray Crystallography and Crystal Chemistry, Institute of General and Ecological Chemistry, Lodz University of Technology, Żeromskiego 116, PL-90924 Łódź, Poland; marcin.swiatkowski@p.lodz.pl

***** Correspondence: Monika.Olesiejuk@polsl.pl; Tel.: +48-32-237-17-29

Table of contents

1. ^{1}H NMR and ^{13}C NMR spectra 2

2. HRMS spectra 50

3. X-ray crystallography data 74

4. **Absorption and emission spectrometry data** 83

1. ^{1}H NMR and ^{13}C NMR spectra

4-Ethyl-3,5-bis[4′-(N,N-diphenylamino)biphenyl-4-yl]-4H-1,2,4-triazole (**7a**).

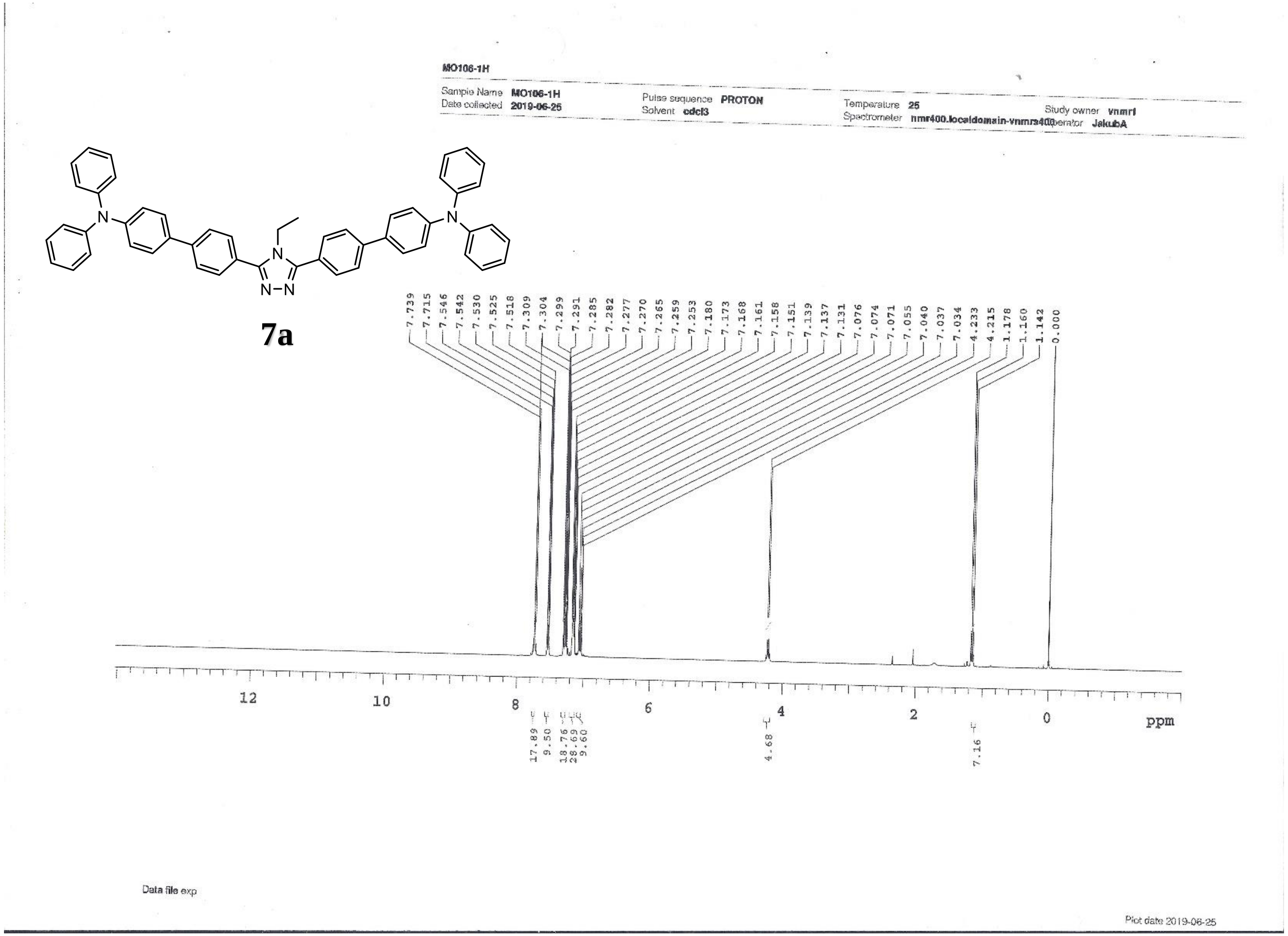

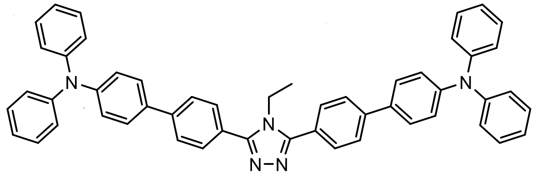

7a

MO106-13C

Sample Name	MO106-13C	Pulse sequence	CARBON	Temperature	25	Study owner	JakubA
Date collected	2019-06-25	Solvent	cdcl3	Spectrometer	nmr400.localdomain-vnmrs400	Operator	JakubA

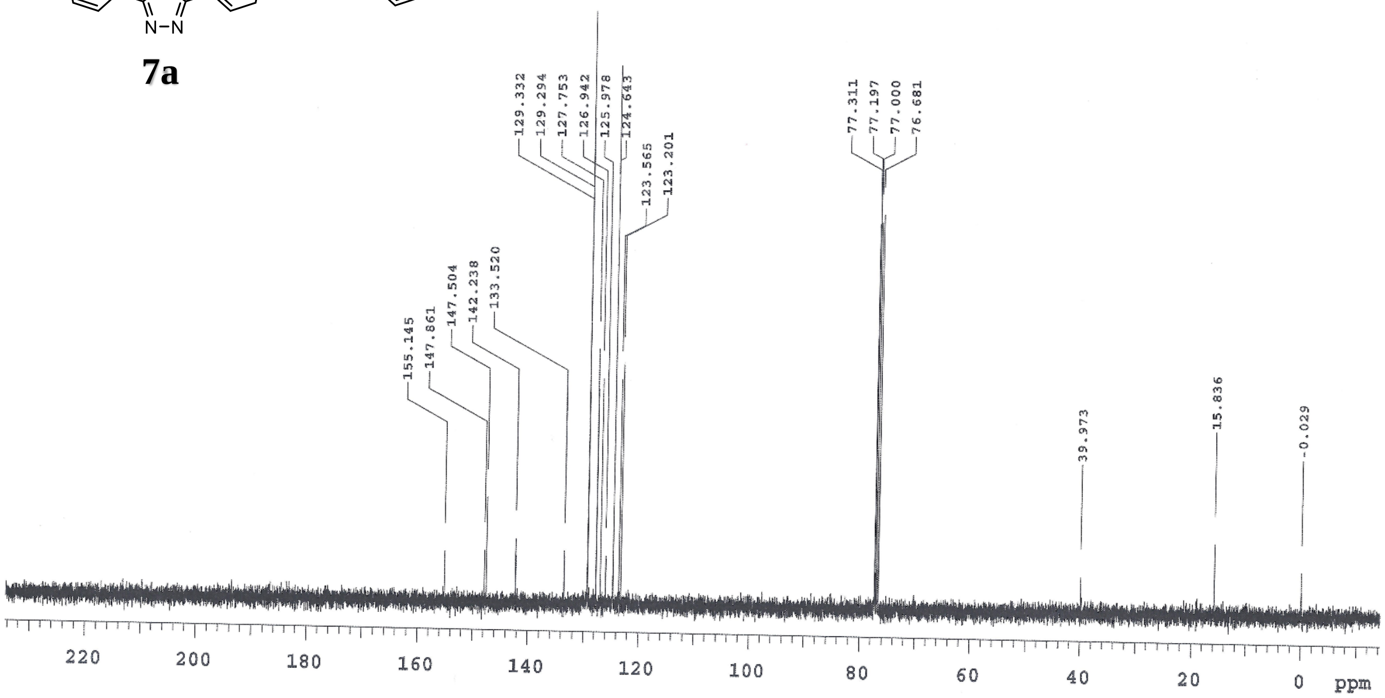

Data file exp

Plot date 2019-06-25

4-Ethyl-3,5-bis[4-(naphthalen-1-yl)phenyl]-4H-1,2,4-triazole (**7b**).

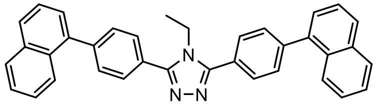

7b

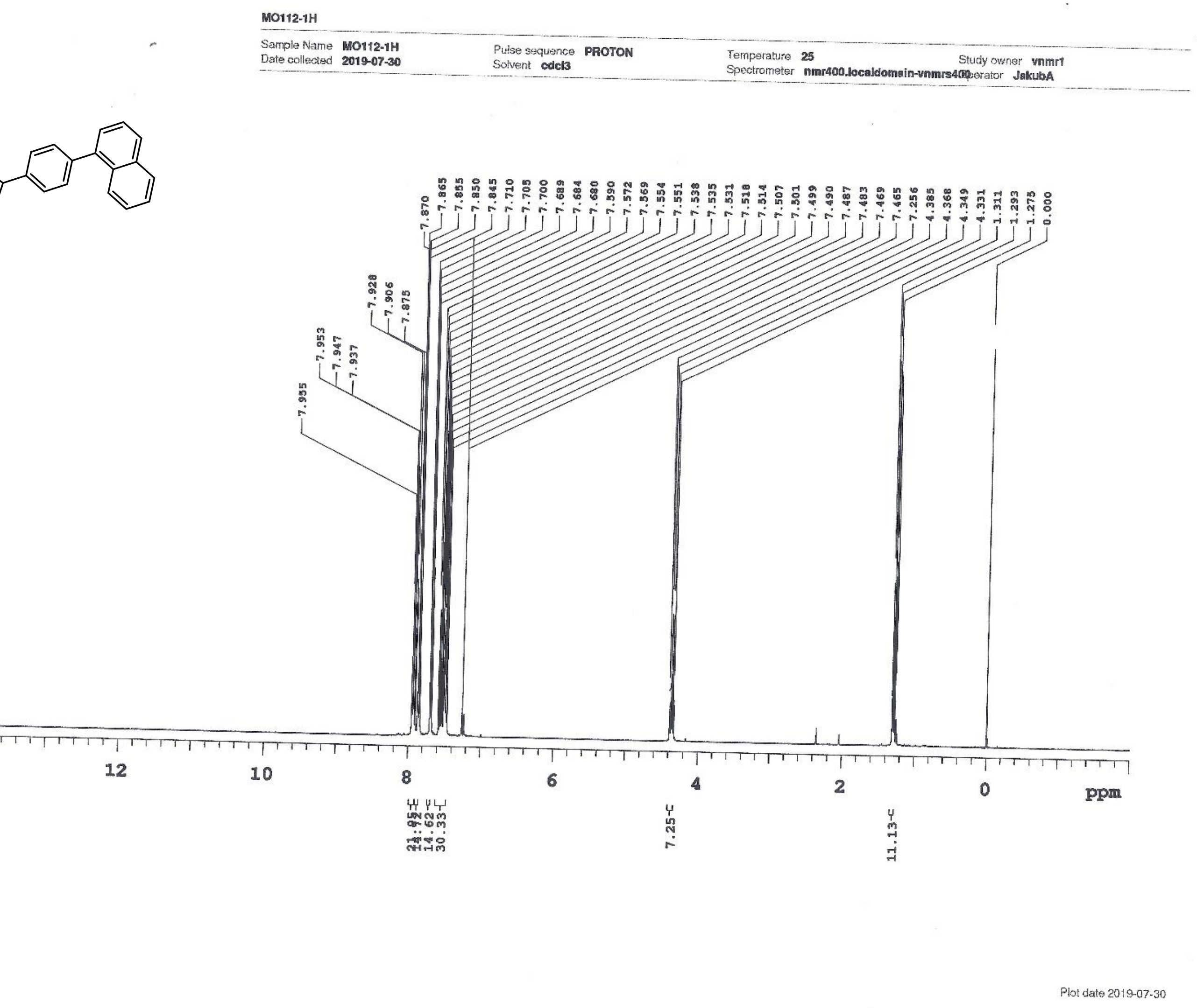

MO112-13C

Sample Name MO112-13C
Date collected 2019-07-09
Pulse sequence CARBON
Solvent cdcl3
Temperature 25
Spectrometer nmr400.localdomain-vnmrs400
Study owner JakubA
Operator JakubA

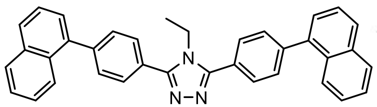

7b

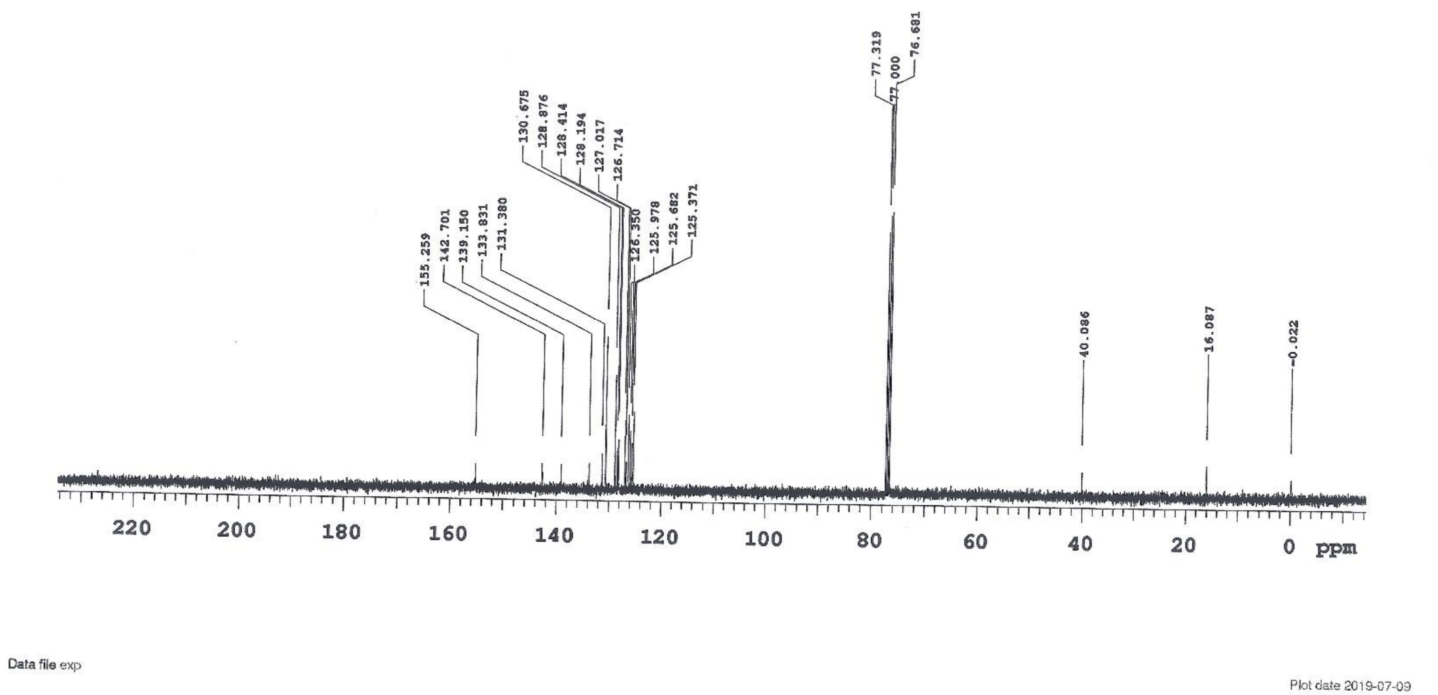

4-Ethyl-3,5-bis[4-(naphthalen-2-yl)phenyl]-4H-1,2,4-triazole (**7c**)

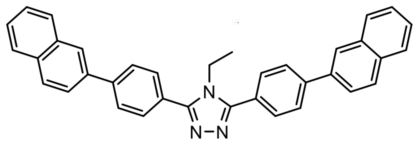

7c

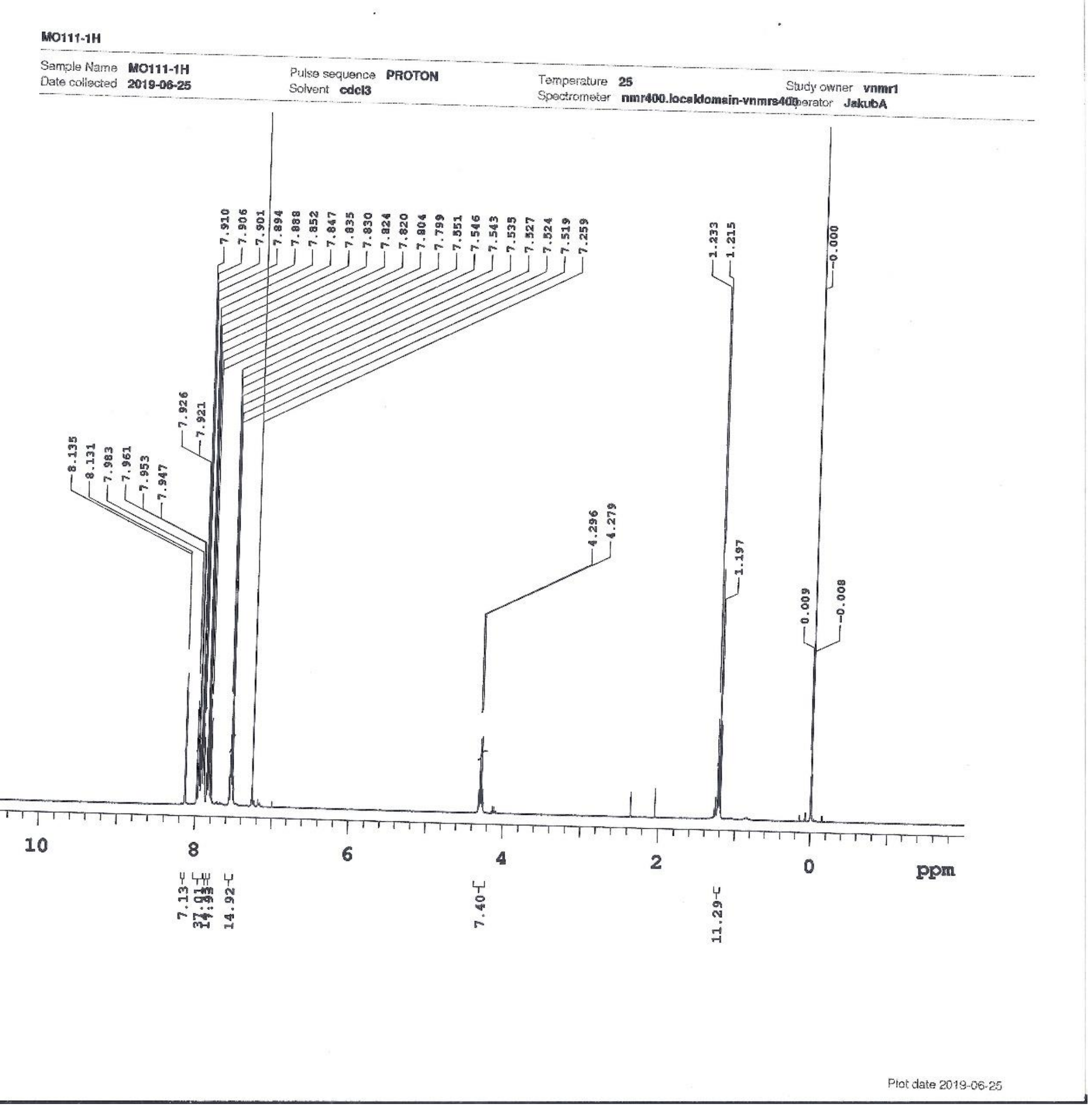

Sample Name
Date collected 2019-06-25
Pulse sequence CARBON
Solvent cdcl3
Temperature 25
Spectrometer nmr400.localdomain-vnmrs400
Study owner JakubA
Operator JakubA

MO-111

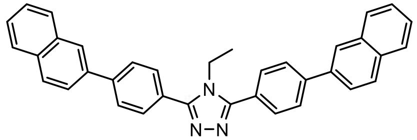

7c

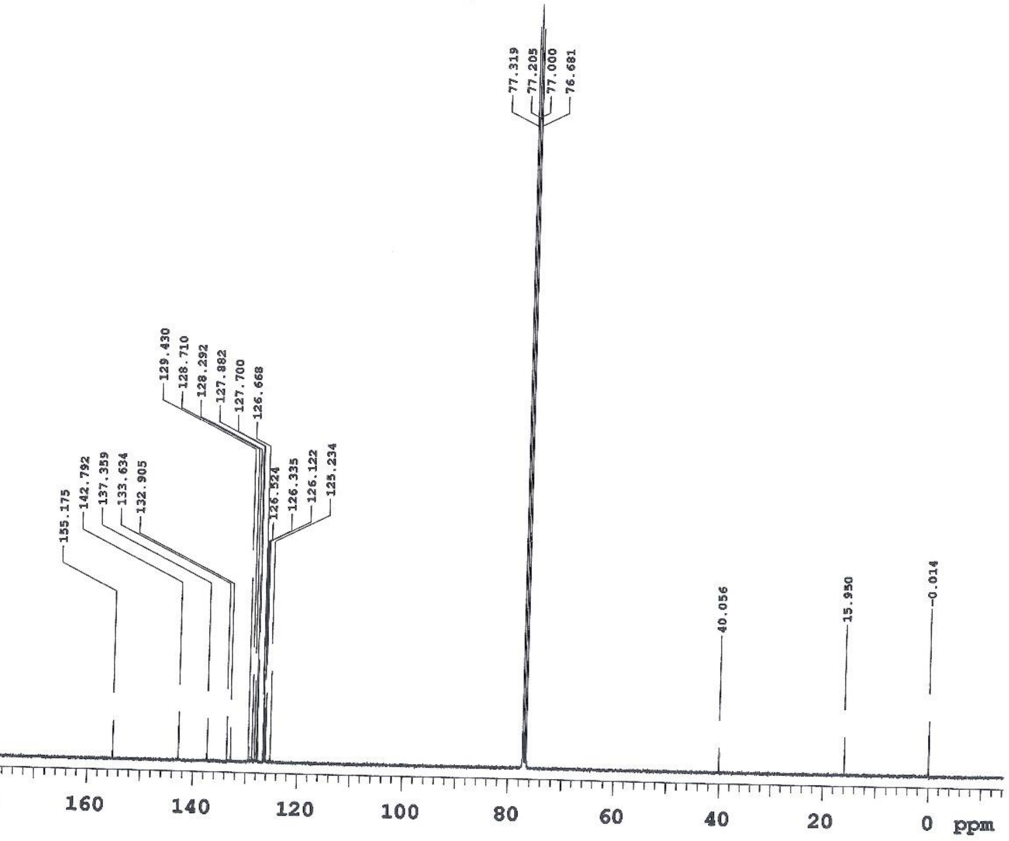

Data file /home/vnmrj_4.2_A/fidlib/Data/AgnieszkaKudelko/2019/Czerwiec/MO111b_13c.fid

Plot date 2019-06-26

4-Ethyl-3,5-bis[4-(quinolin-3-yl)phenyl]-4H-1,2,4-triazole (**7d**).

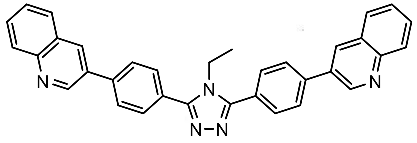

7d

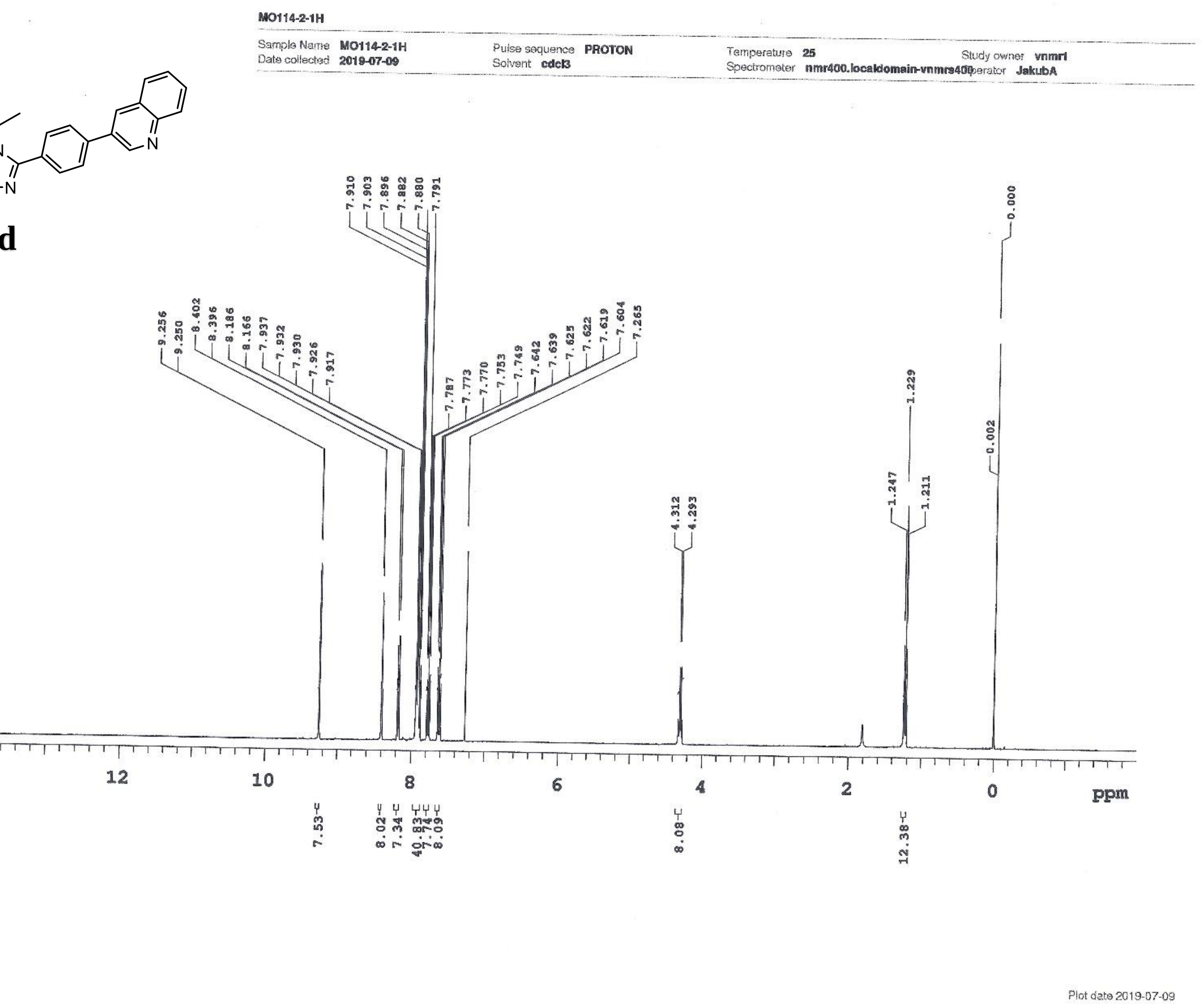

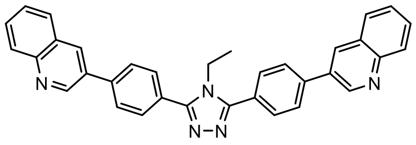

7d

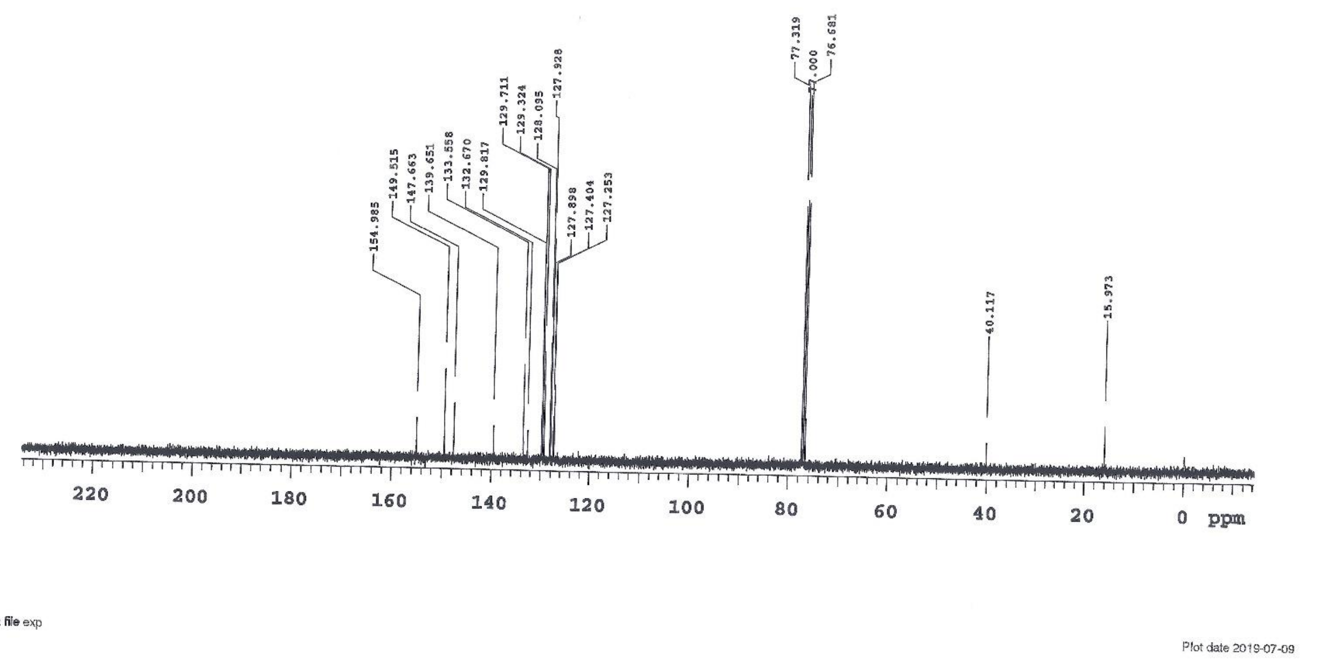
MO114-2-13C
Sample Name MO114-2-13C
Date collected 2019-07-09
Pulse sequence CARBON
Solvent cdcl3
Temperature 25
Spectrometer nmr400.localdomain-vnmrs400
Study owner JakubA
Operator JakubA
154.985
149.515
147.663
139.651
133.558
132.670
129.817
129.711
129.324
128.095
127.928
127.898
127.404
127.253
77.319
77.000
76.681
40.117
15.973
220
200
180
160
140
120
100
80
60
40
20
0
ppm
Data file exp
Plot date 2019-07-09

4-Ethyl-3,5-bis[4-(quinolin-6-yl)phenyl]-4H-1,2,4-triazole (**7e**).

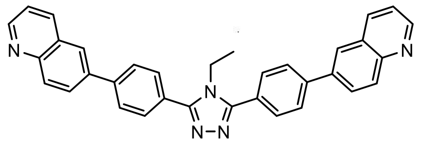

7e

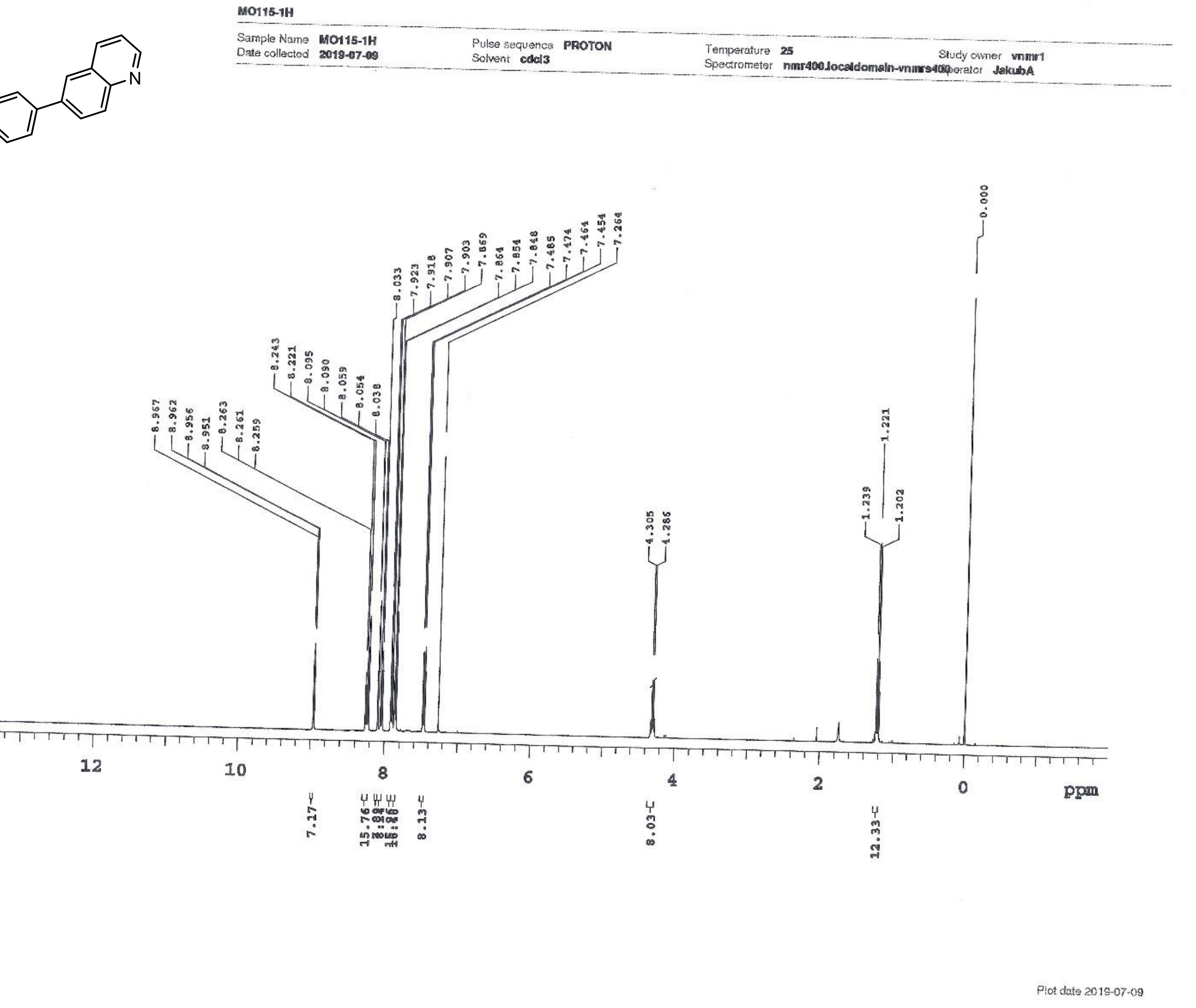

MO115-13C

Sample Name MO115-13C
Date collected 2019-07-09

Pulse sequence CARBON
Solvent cdcl3

Temperature 25
Spectrometer nmr400.localdomain-vnmrs400

Study owner JakubA
Operator JakubA

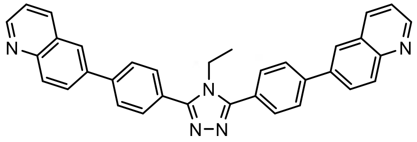

7e

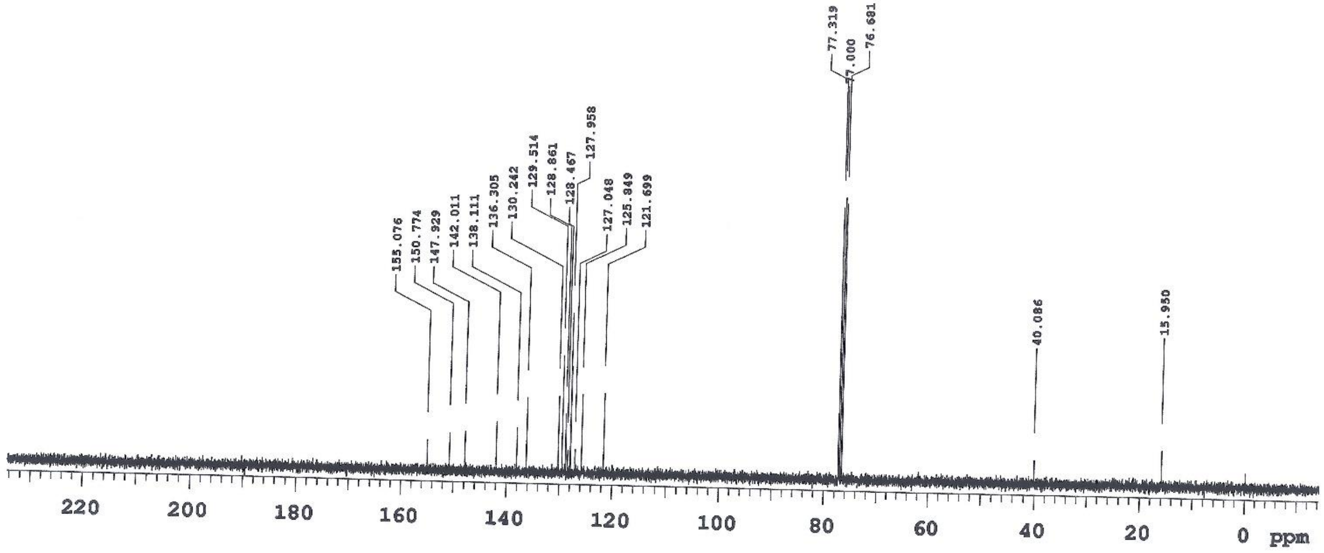

Data file exp

Plot date 2019-07-09

3,5-Bis[4-(dibenzothiophen-4-yl)phenyl]-4-ethyl-4H-1,2,4-triazole (**7f**).

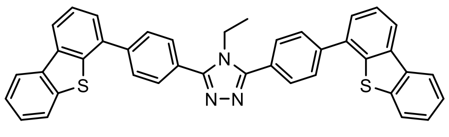

7f

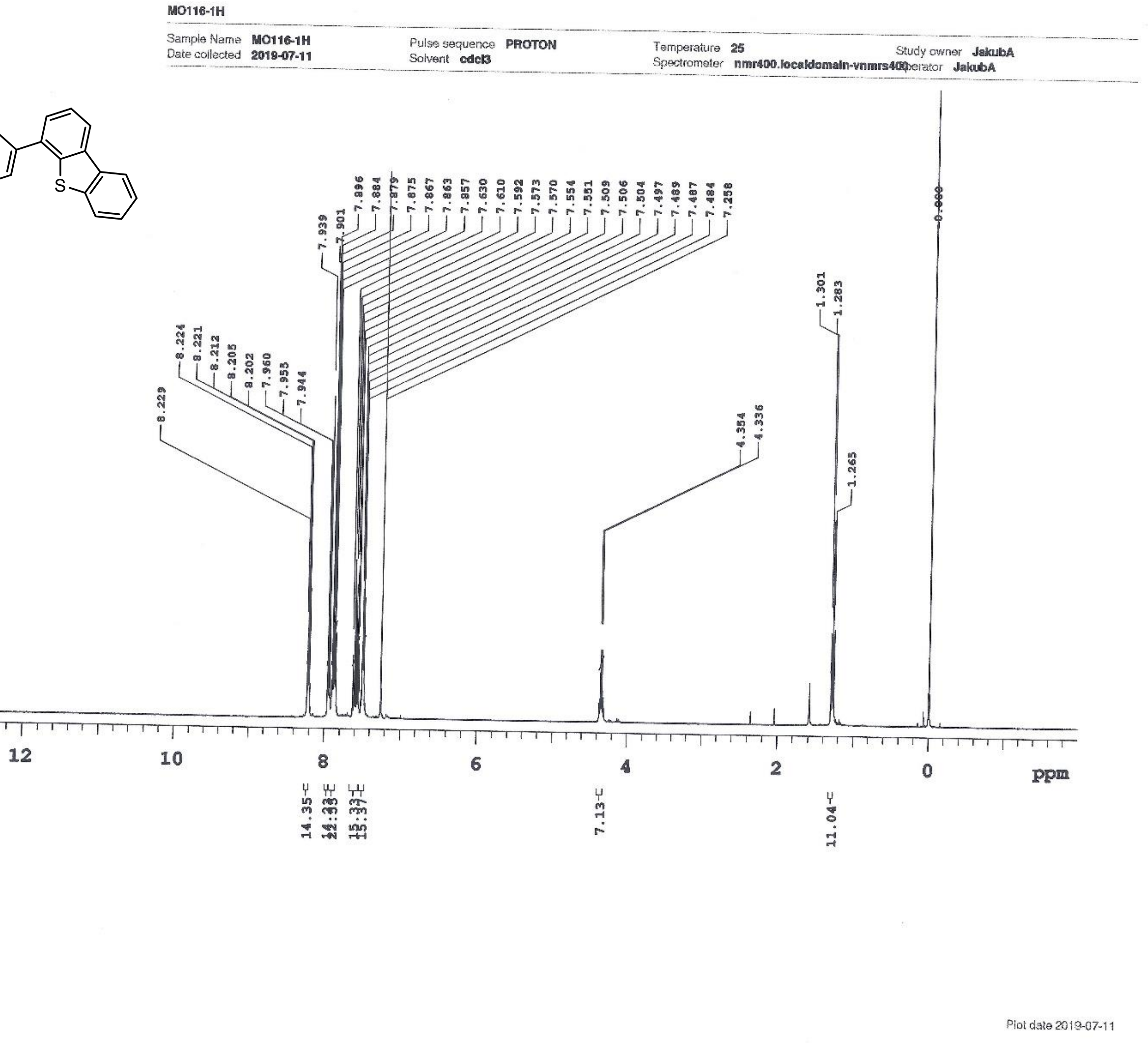

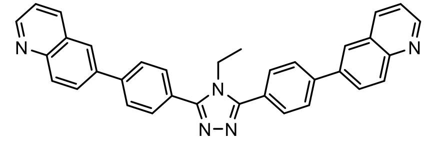

7f

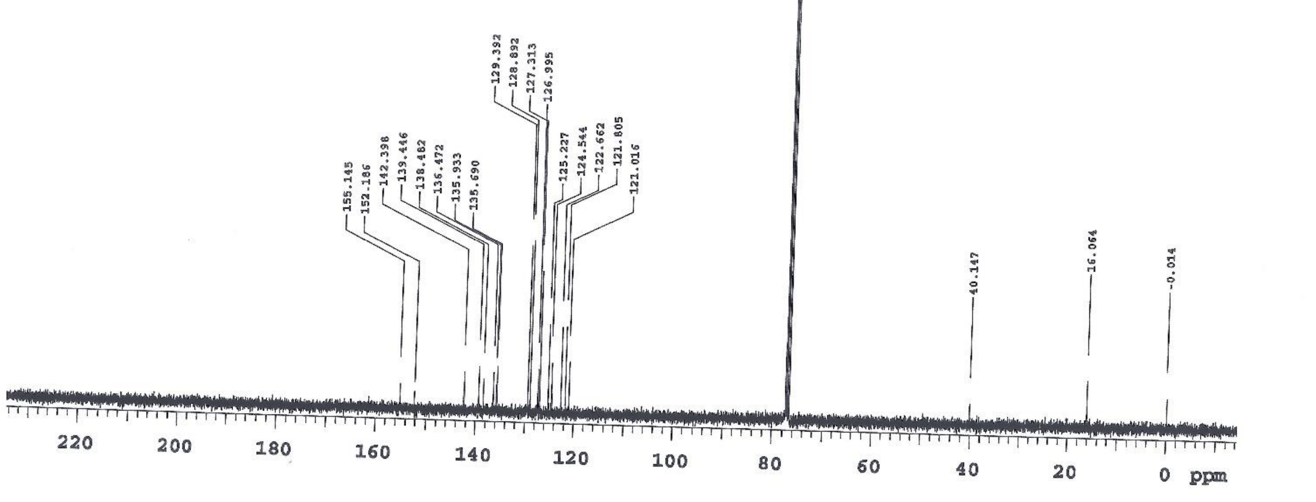
MO116-13C
Sample Name MO116-13C
Date collected 2019-07-11
Pulse sequence CARBON
Solvent cdcl3
Temperature 25
Spectrometer nmr400.localdomain-vnmrs400
Study owner JakubA
Operator JakubA
77.319
77.205
77.000
76.689
129.392
128.892
127.313
126.995
155.145
152.186
142.398
139.446
138.482
136.472
135.933
135.690
125.227
124.544
122.662
121.805
121.016
40.147
16.064
-0.014
220
200
180
160
140
120
100
80
60
40
20
0 ppm
Data file exp
Plot date 2019-07-11

3,5-Bis[4-(dibenzofuran-4-yl)phenyl]-4-ethyl-4H-1,2,4-triazole (**7g**).

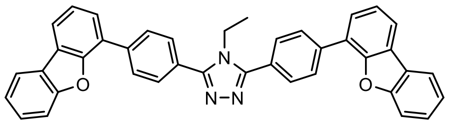

7g

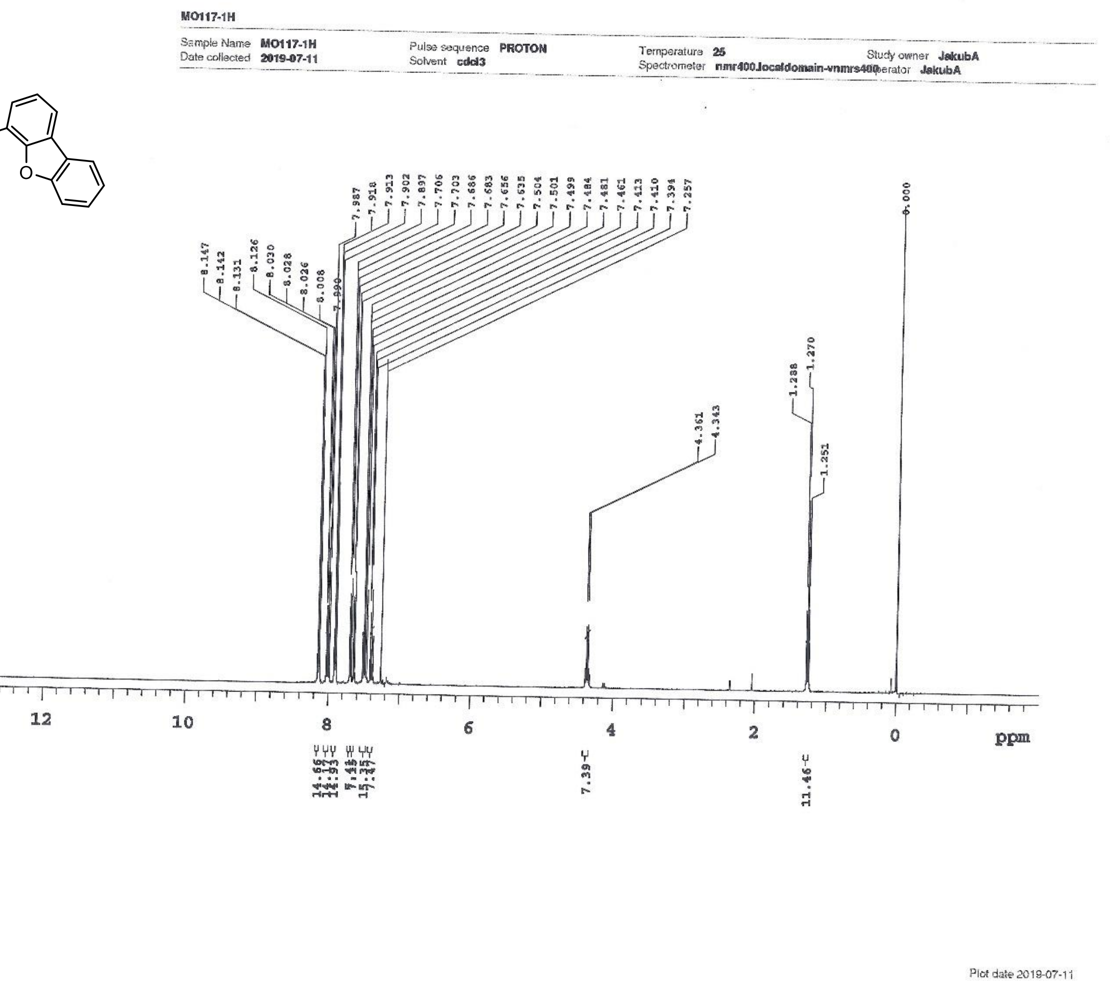

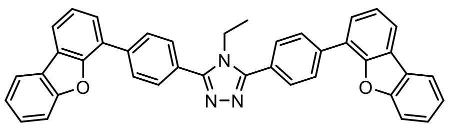

7g

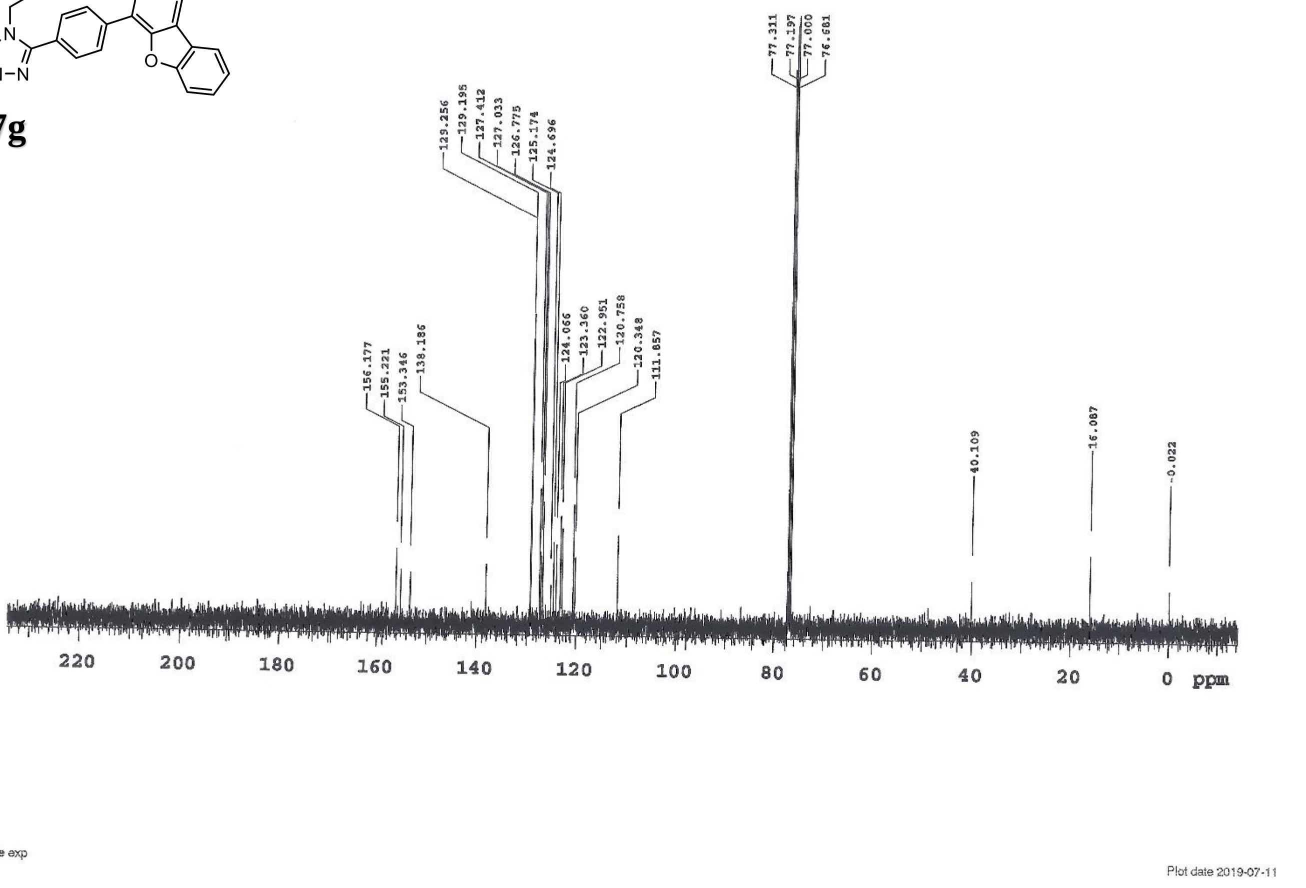
MO117-13C
Sample Name MO117-13C
Date collected 2019-07-11
Pulse sequence CARBON
Solvent cdcl3
Temperature 25
Spectrometer nmr400.localdomain-vnmrs400
Study owner JakubA
Operator JakubA
156.177
155.221
153.346
138.186
129.256
129.195
127.412
127.033
126.775
125.174
124.696
124.066
123.360
122.951
120.758
120.348
111.857
77.311
77.197
77.000
76.681
40.109
16.087
-0.022
220 200 180 160 140 120 100 80 60 40 20 0 ppm
Data file exp
Plot date 2019-07-11

4-Ethyl-3,5-bis[4-(9-methyl-9H-carbazol-3-yl)phenyl]-4H-1,2,4-triazole (**7h**).

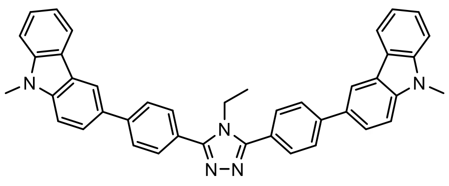

7h

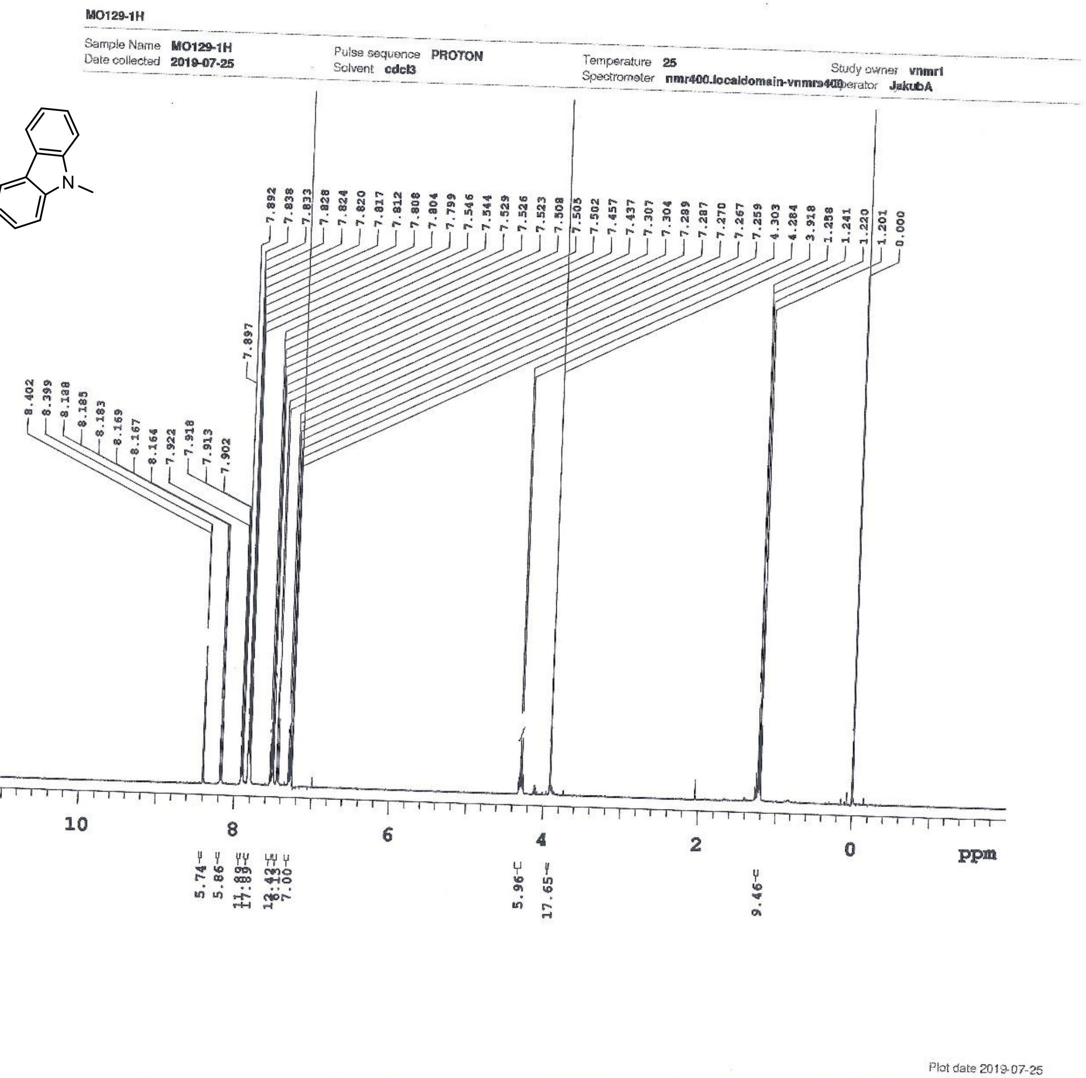

MO129-13C

Sample Name MO129-13C
Date collected 2019-07-25
Pulse sequence CARBON
Solvent cdcl3
Temperature 25
Spectrometer nmr400.localdomain-vnmrs400
Study owner JakubA
Operator JakubA

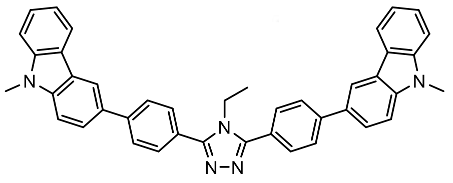

7h

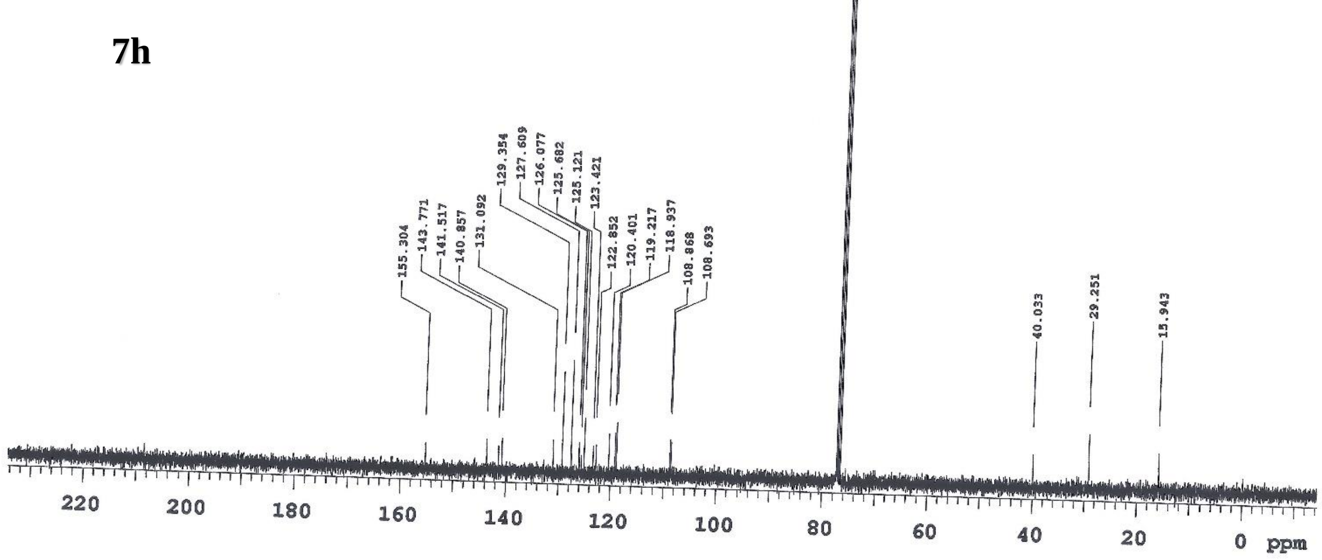

Data file exp

Plot date 2019-07-25

4-Ethyl-3,5-bis[4-(9-ethyl-9H-carbazol-3-yl)phenyl]-4H-1,2,4-triazole (**7i**).

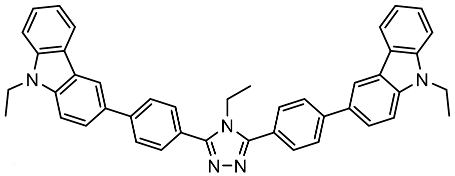

7i

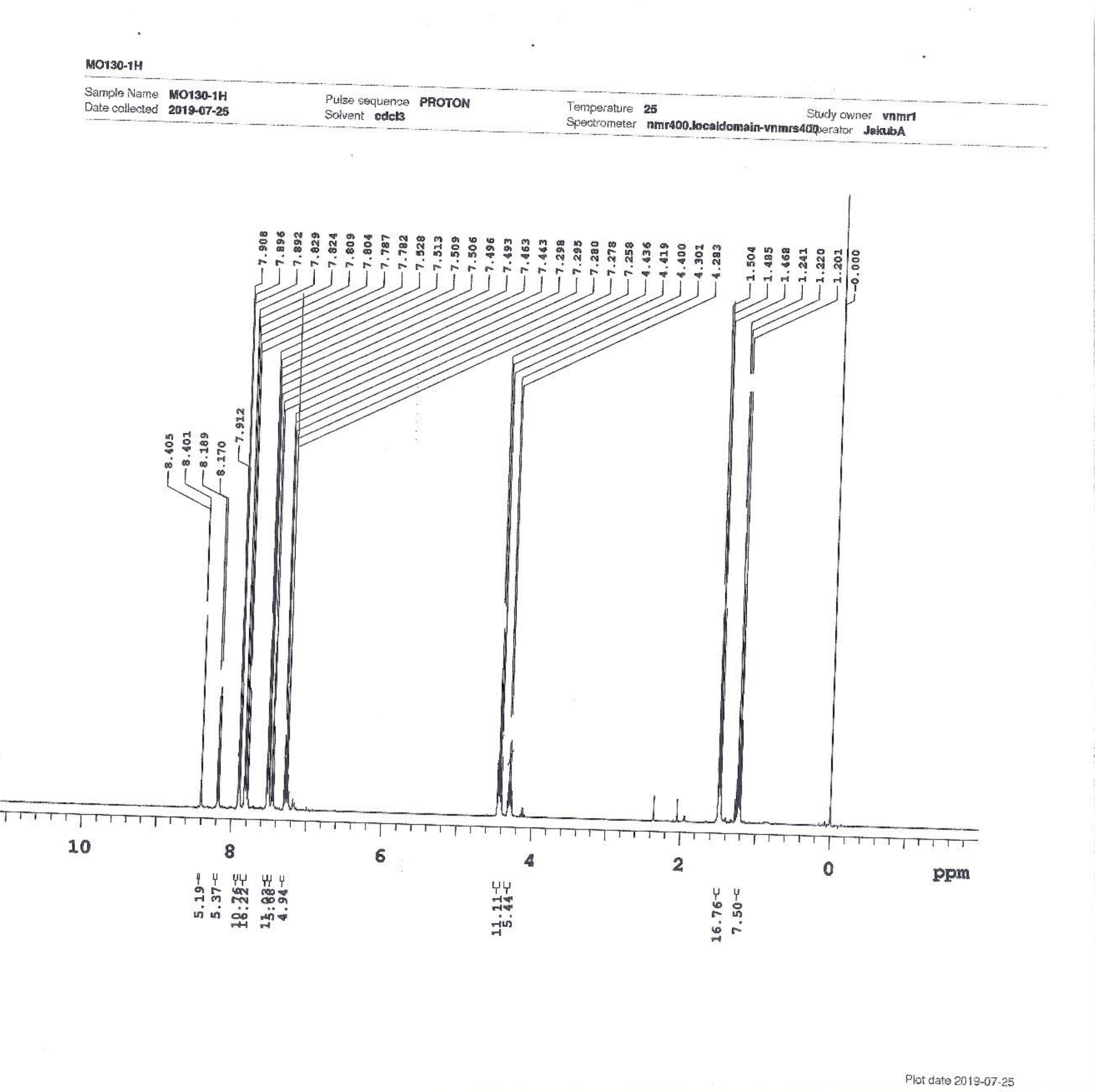

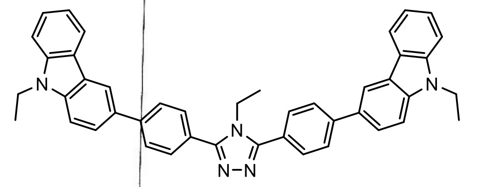
N
N
N-N
N

7i

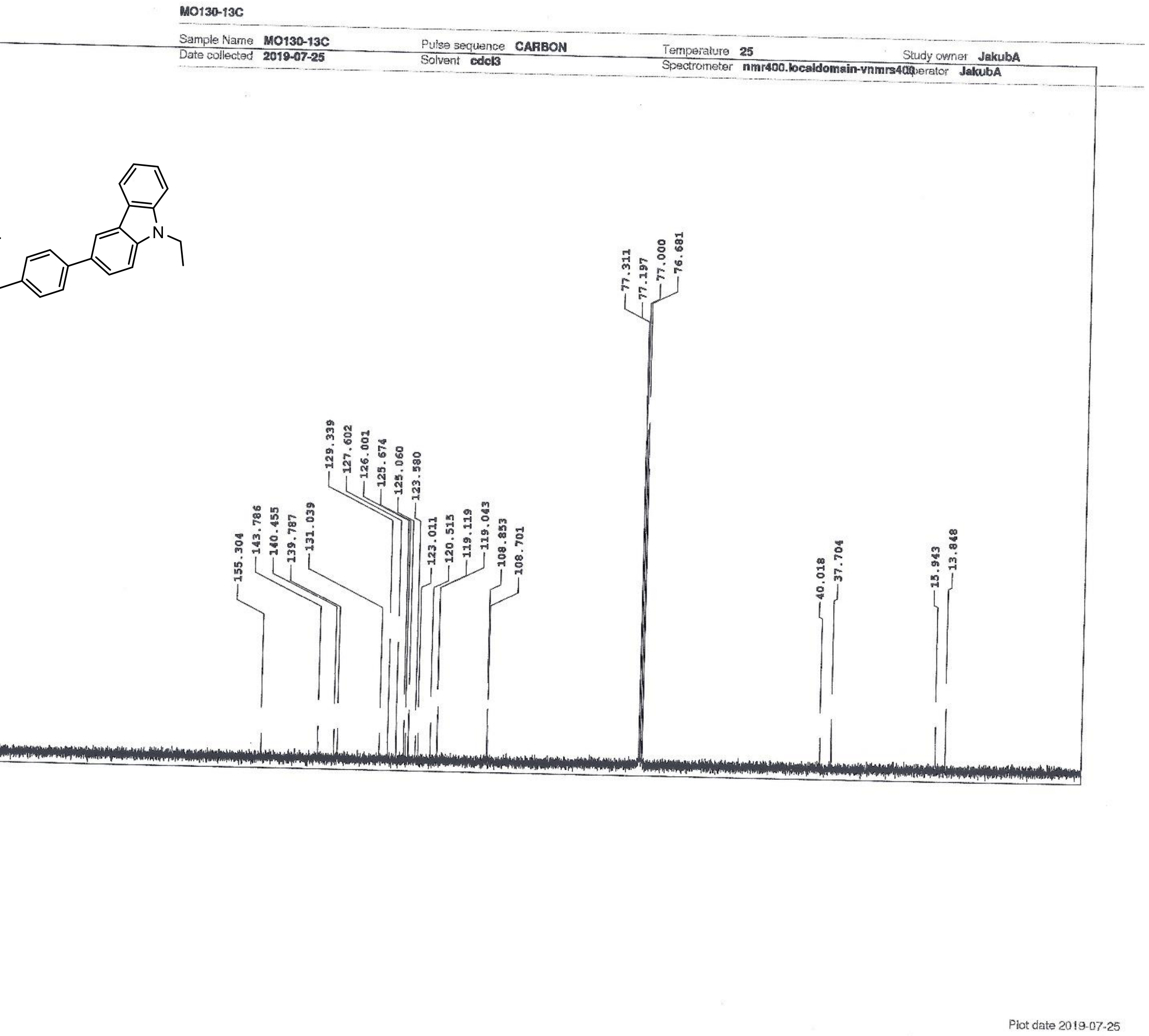
MO130-13C
Sample Name MO130-13C
Date collected 2019-07-25
Pulse sequence CARBON
Solvent cdcl3
Temperature 25
Spectrometer nmr400.localdomain-vnmrs400
Study owner JakubA
Operator JakubA
155.304
143.786
140.455
139.787
131.039
129.339
127.602
126.001
125.674
125.060
123.580
123.011
120.515
119.119
119.043
108.853
108.701
77.311
77.197
77.000
76.681
40.018
37.704
15.943
13.848
Data file exp
Plot date 2019-07-25

3,5-Bis[4′-(9H-carbazol-9-yl)biphenyl-4-yl]-4-ethyl-4H-1,2,4-triazole (**7j**).

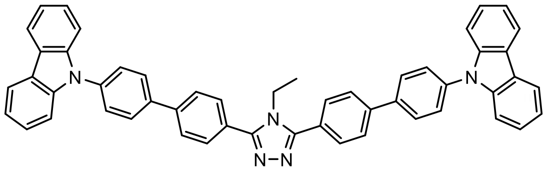

7j

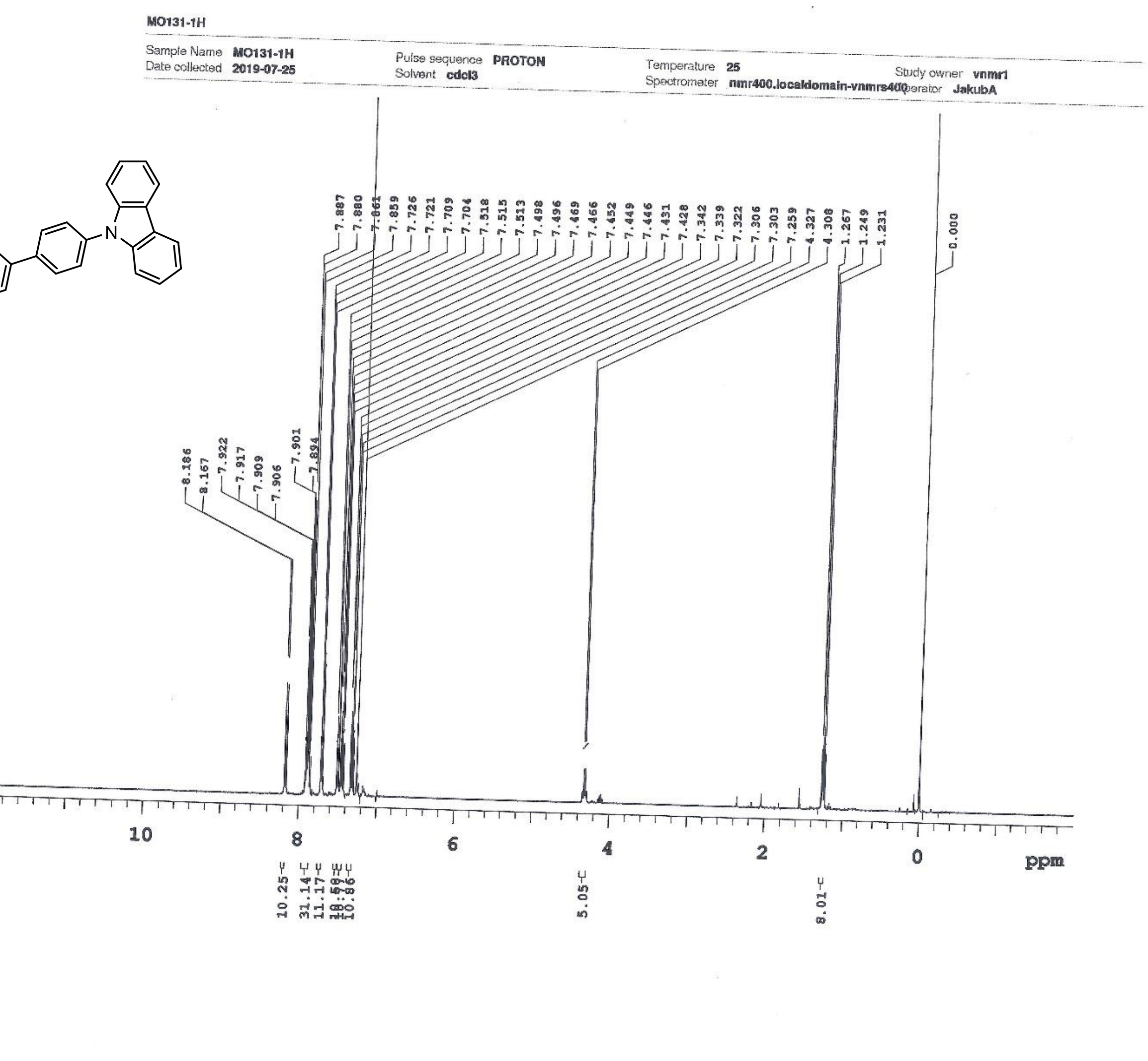

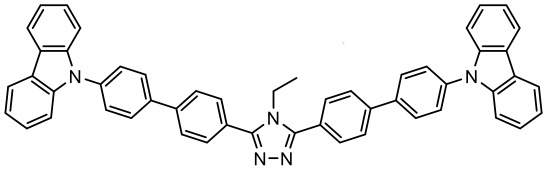

7j

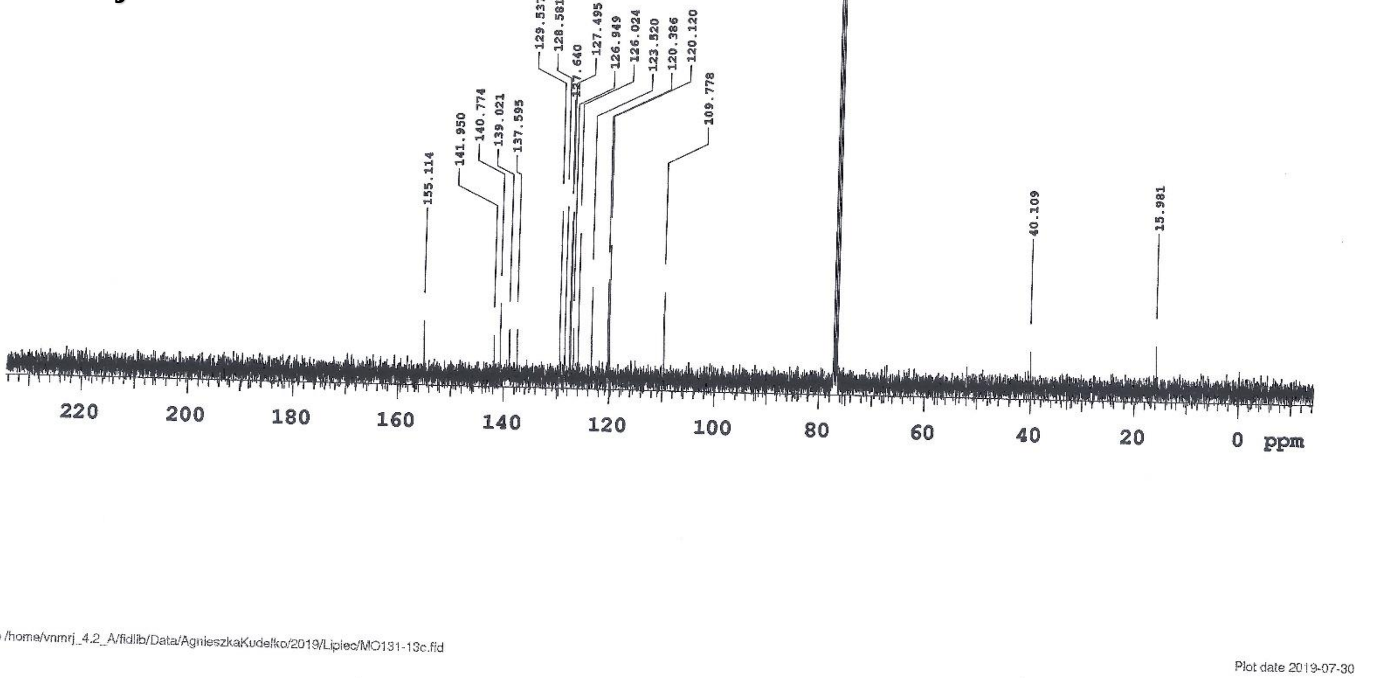
MO131-13C
Sample Name MO131-13C
Date collected 2019-07-25
Pulse sequence CARBON
Solvent cdcl3
Temperature 25
Spectrometer nmr400.localdomain-vnmrs400
Study owner JakubA
Operator JakubA
155.114
141.950
140.774
139.021
137.595
129.537
128.581
127.640
127.495
126.949
126.024
123.520
120.386
120.120
109.778
77.319
77.205
77.000
76.681
40.109
15.981
220 200 180 160 140 120 100 80 60 40 20 0 ppm
Data file /home/vnmrj_4.2_A/fidlib/Data/AgnieszkaKudelko/2019/Lipiec/MO131-13c.fid
Plot date 2019-07-30

4-Ethyl-3,5-bis[4-(thiantren-1-yl)phenyl]-4H-1,2,4-triazole (**7k**).

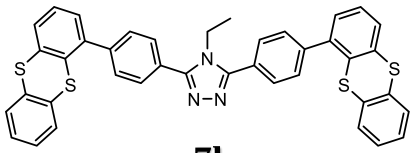

7k

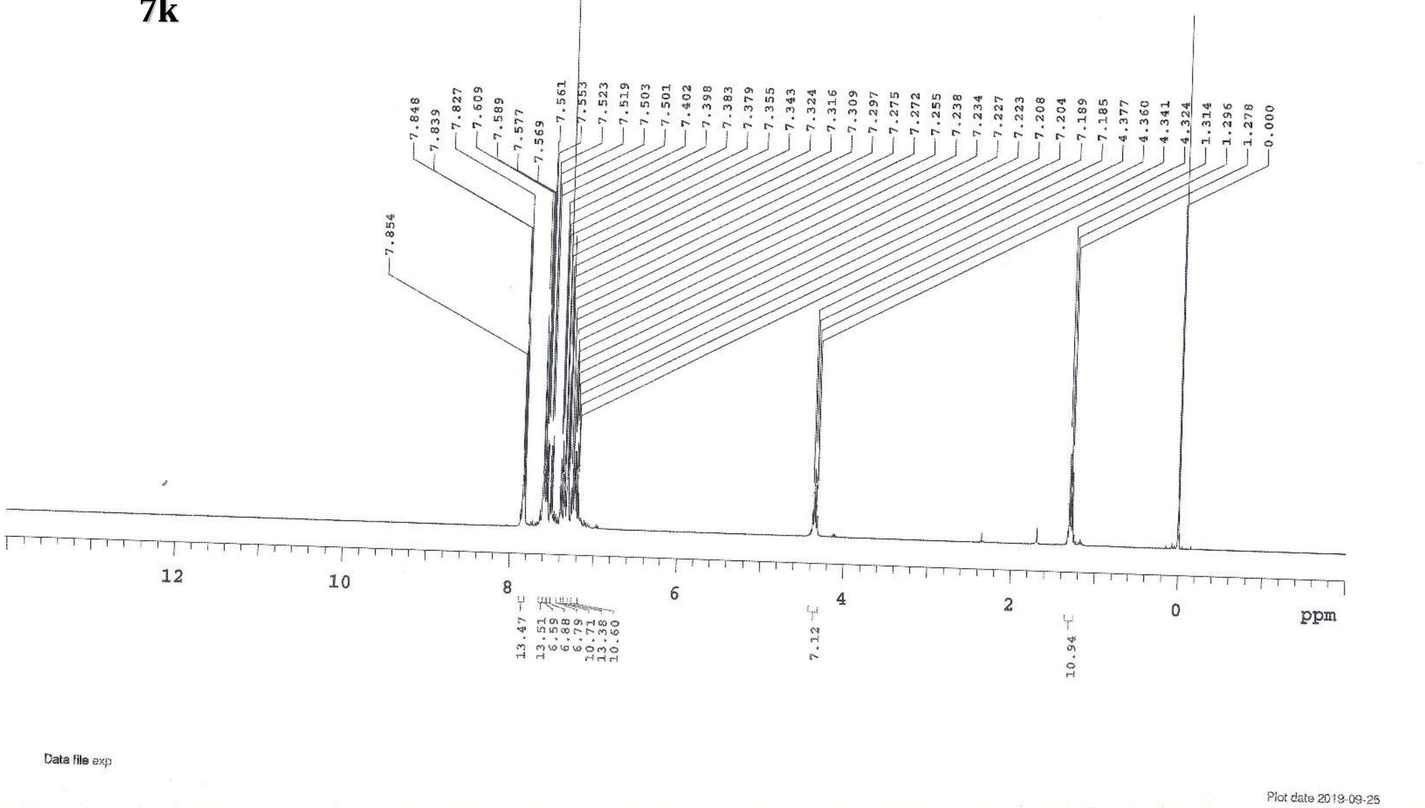

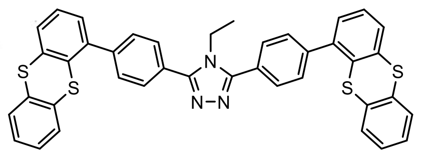

7k

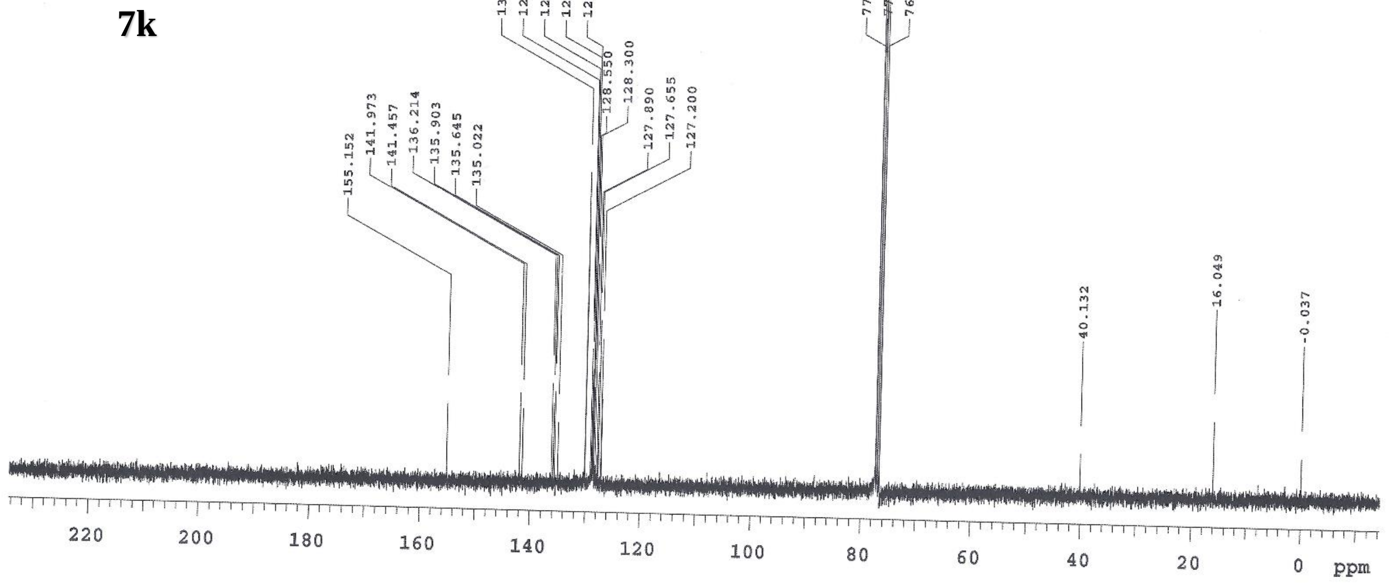
MO120-13C
Sample Name MO120-13C
Date collected 2019-09-25
Pulse sequence CARBON
Solvent cdcl3
Temperature 25
Spectrometer nmr400.localdomain-vnmrs400
Study owner JakubA
Operator JakubA
155.152
141.973
141.457
136.214
135.903
135.645
135.022
130.113
129.112
128.884
128.694
128.641
128.550
128.300
127.890
127.655
127.200
77.319
77.000
76.681
40.132
16.049
-0.037
220 200 180 160 140 120 100 80 60 40 20 0 ppm
Data file exp
Plot date 2019-09-25

3,5-Bis[4′-(N,N-diphenylamino)biphenyl-4-yl]-4-propyl-4H-1,2,4-triazole (**8a**).

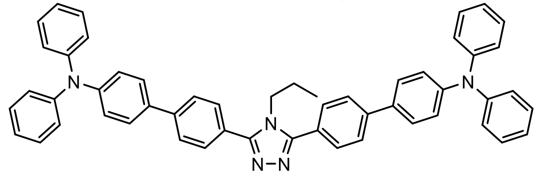

8a

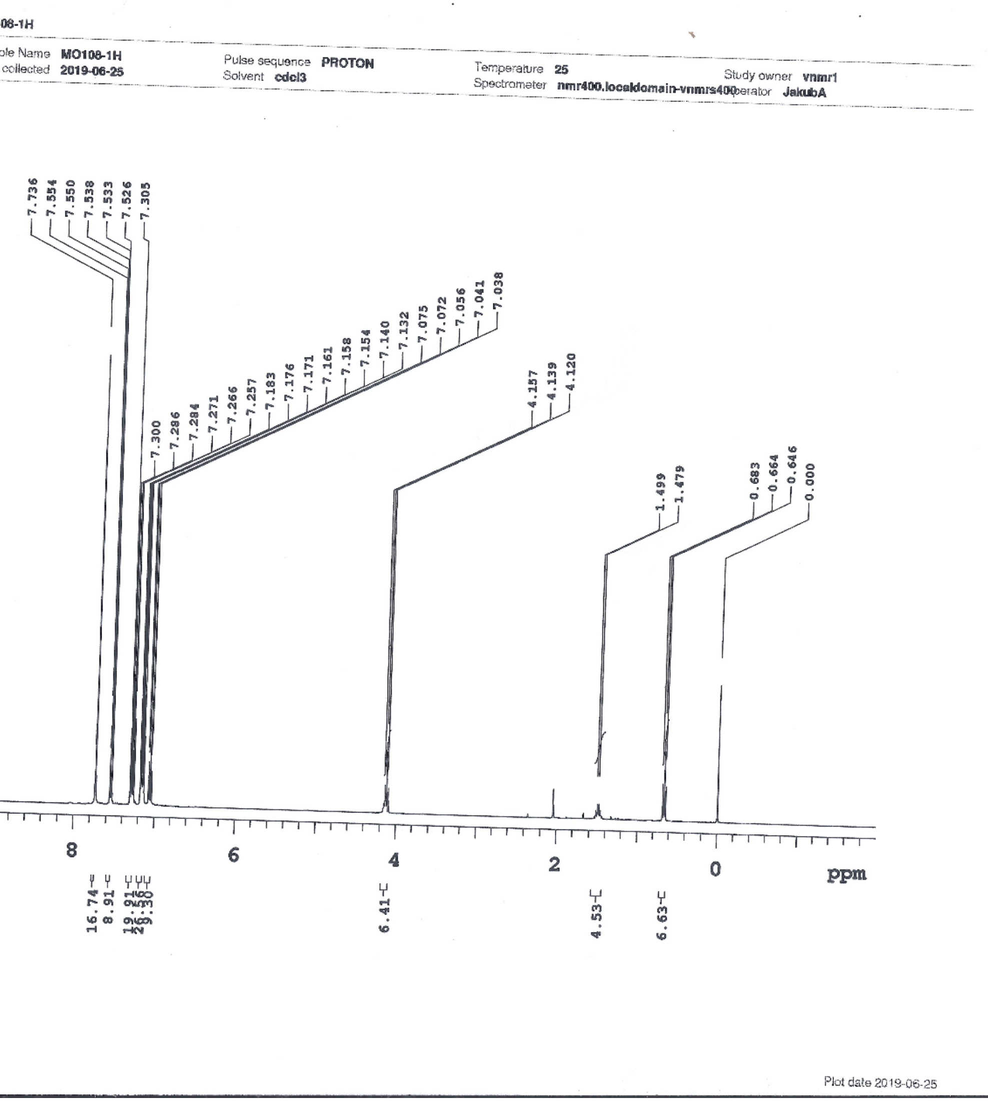

MO108-13C

Sample Name MO108-13C
Date collected 2019-06-25
Pulse sequence CARBON
Solvent cdcl3
Temperature 25
Spectrometer nmr400.localdomain-vnmrs400
Study owner JakubA
Operator JakubA

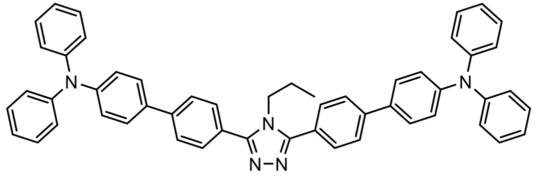

8a

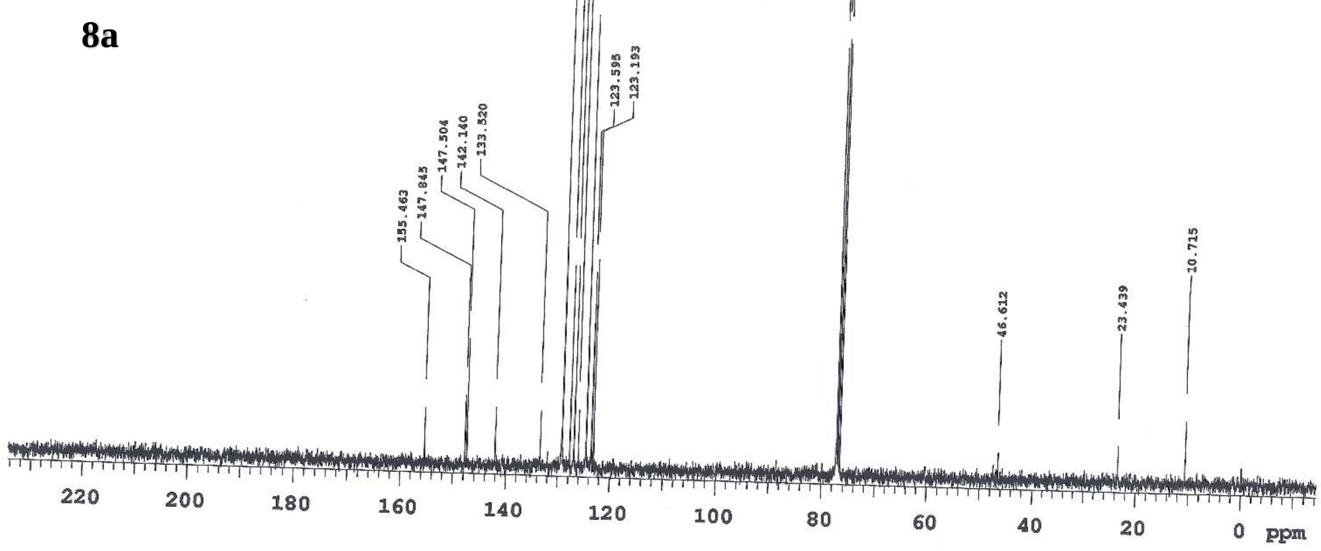

Data file exp

Plot date 2019-06-25

4-Butyl-3,5-bis[4′-(N,N-diphenylamino)biphenyl-4-yl]-4H-1,2,4-triazole (**9a**).

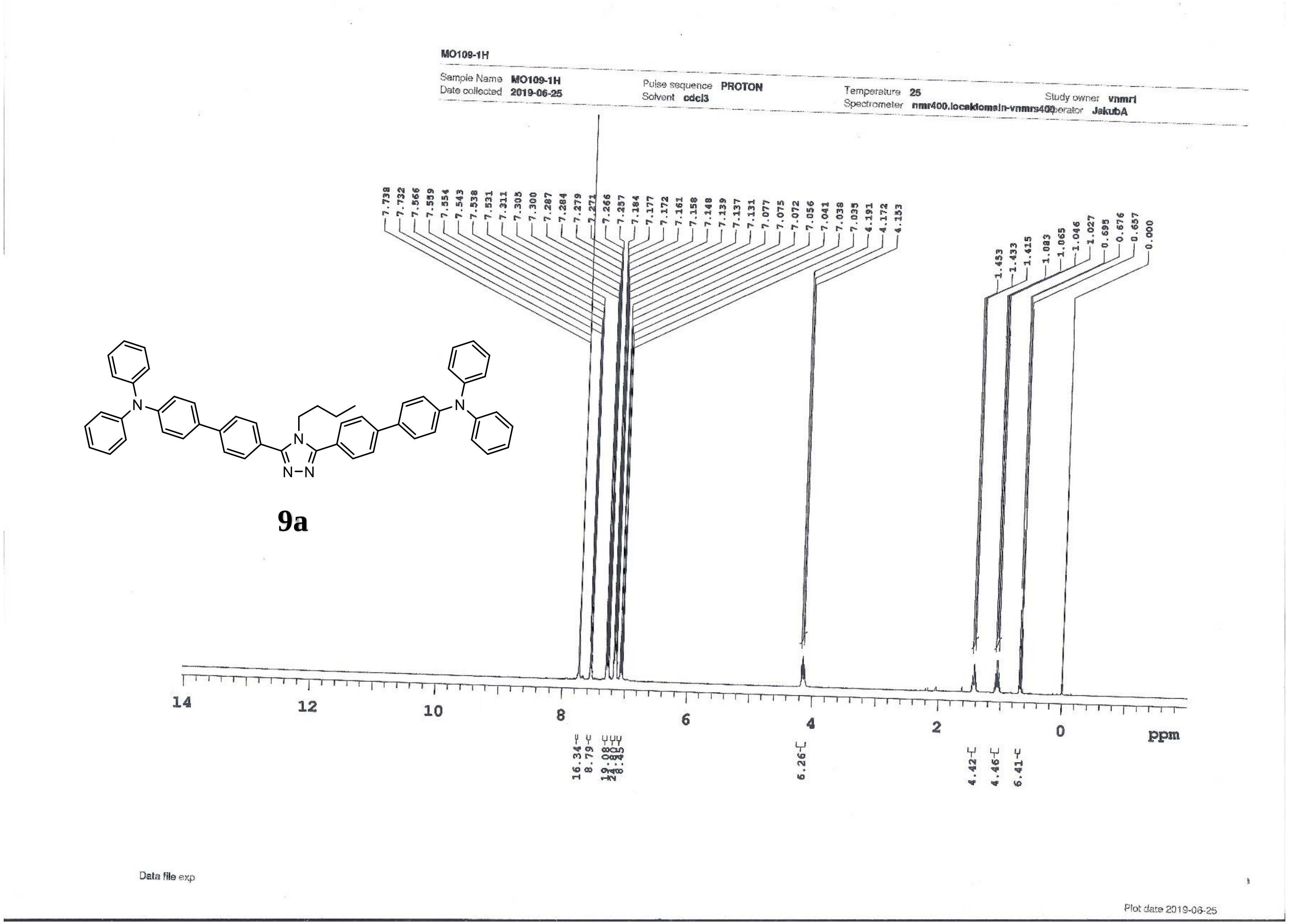

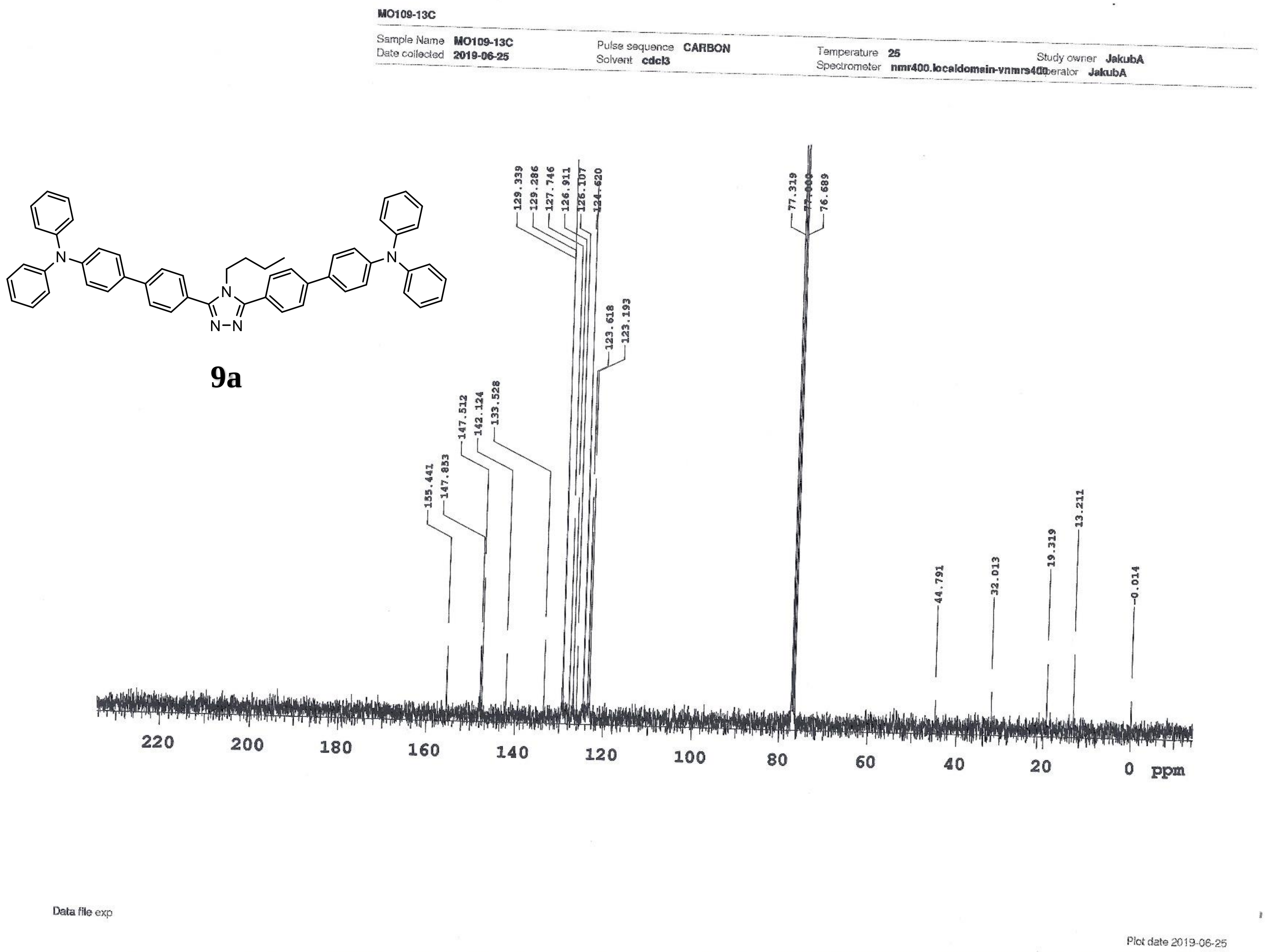

9a

MO109-13C
Sample Name MO109-13C
Date collected 2019-06-25
Pulse sequence CARBON
Solvent cdcl3
Temperature 25
Spectrometer nmr400.localdomain-vnmrs400
Study owner JakubA
Operator JakubA
129.339
129.286
127.746
126.911
126.107
124.620
123.618
123.193
155.441
147.853
147.512
142.124
133.528
77.319
76.689
44.791
32.013
19.319
13.211
-0.014
220
200
180
160
140
120
100
80
60
40
20
0 ppm
Data file exp
Plot date 2019-06-25

4-Butyl-3,5-bis[4-(naphthalen-1-yl)phenyl]-4H-1,2,4-triazole (**9b**).

9b

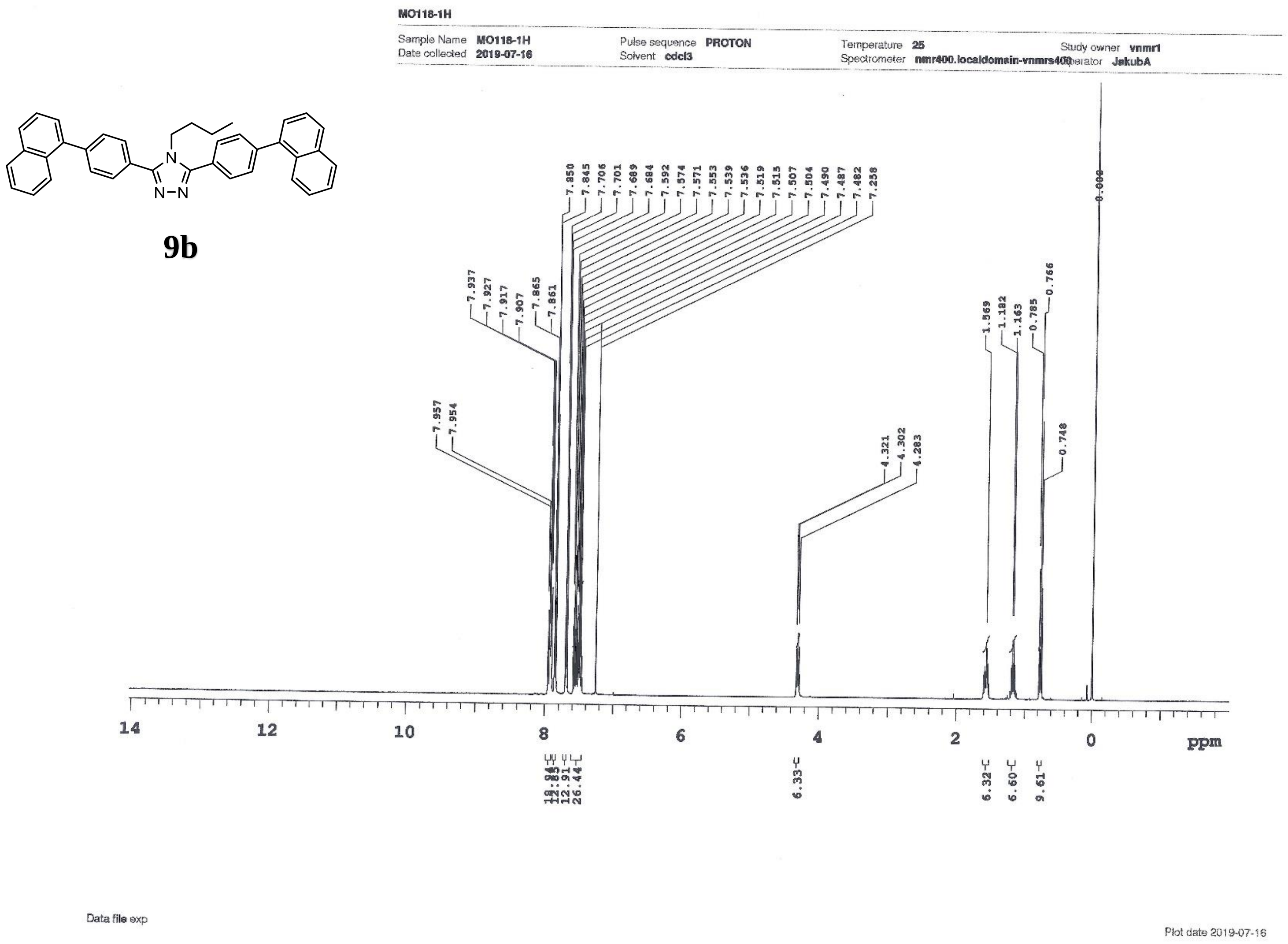

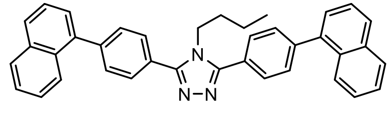

9b

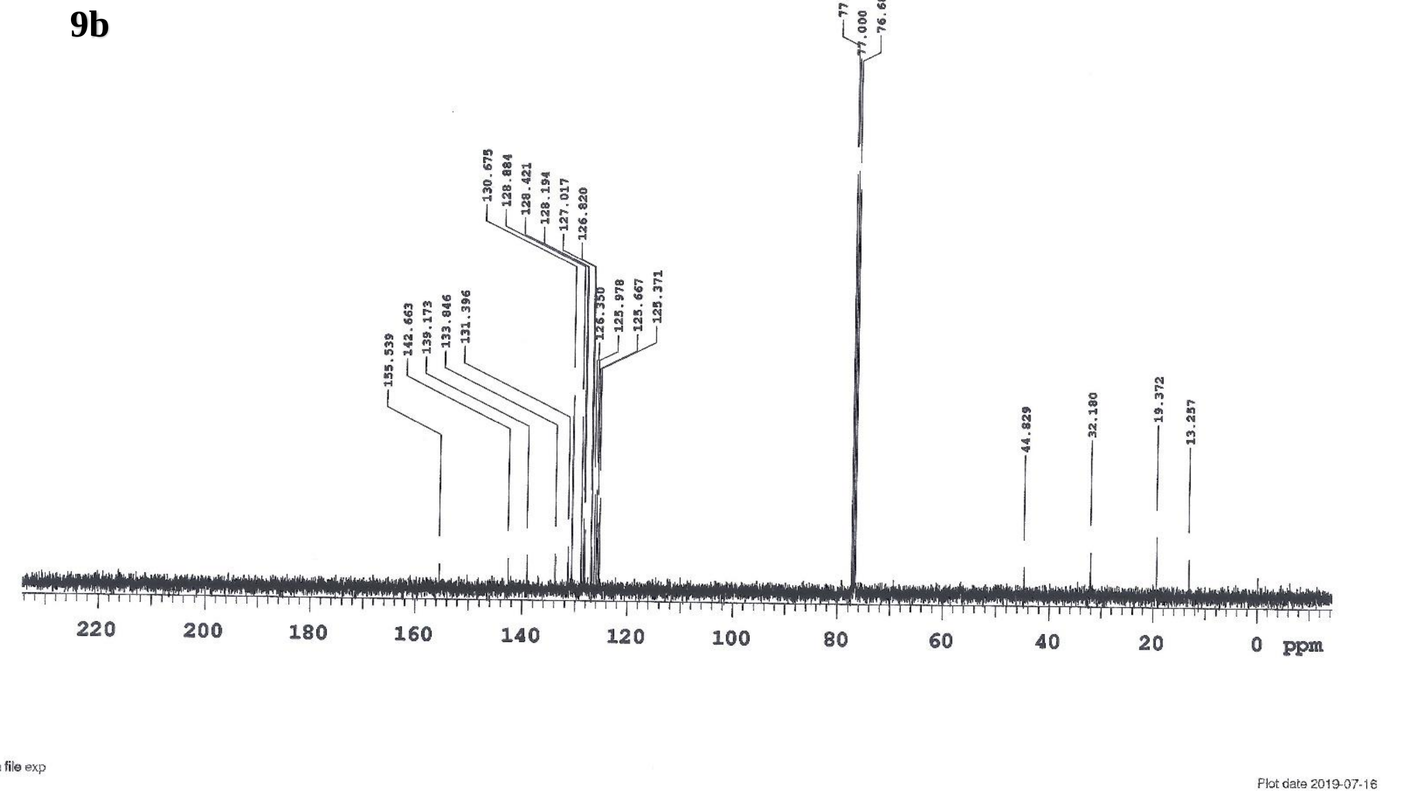
MO118-13C
Sample Name MO118-13C
Date collected 2019-07-16
Pulse sequence CARBON
Solvent cdcl3
Temperature 25
Spectrometer nmr400.localdomain-vnmrs400
Study owner JakubA
Operator JakubA
155.539
142.663
139.173
133.846
131.396
130.675
128.884
128.421
128.194
127.017
126.820
126.350
125.978
125.667
125.371
77.319
77.000
76.681
44.829
32.180
19.372
13.257
220 200 180 160 140 120 100 80 60 40 20 0 ppm
Data file exp
Plot date 2019-07-16

4-Butyl-3,5-bis[4-(naphthalen-2-yl)phenyl]-4H-1,2,4-triazole (**9c**).

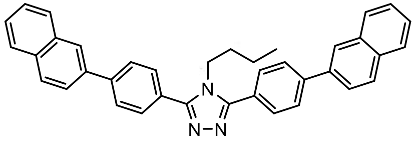

9c

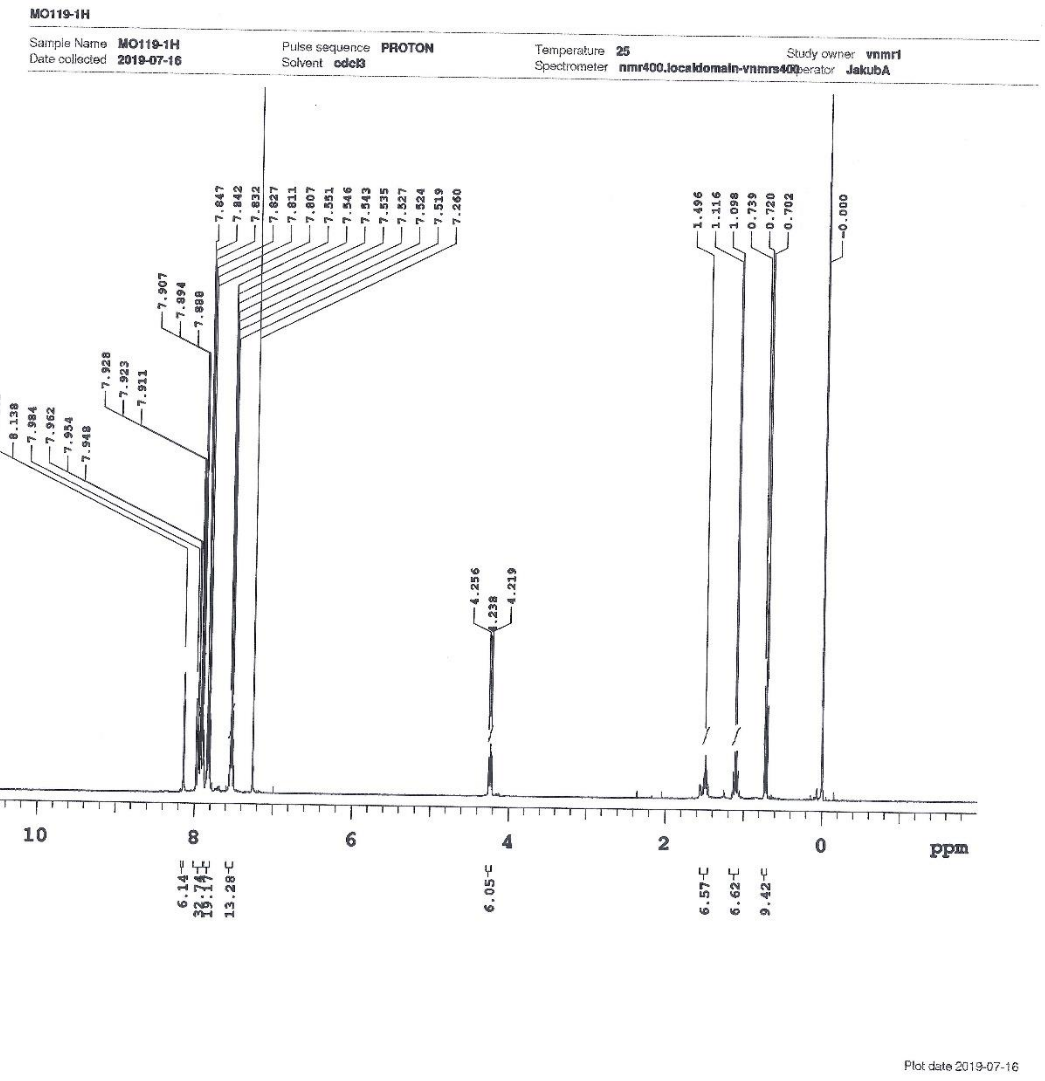

MO119-13C

Sample Name **MO119-13C** Pulse sequence **CARBON** Temperature **25** Study owner **JakubA**
Date collected **2019-07-16** Solvent **cdcl3** Spectrometer **nmr400.localdomain-vnmrs400** Operator **JakubA**

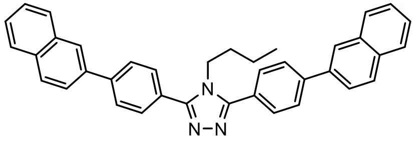

9c

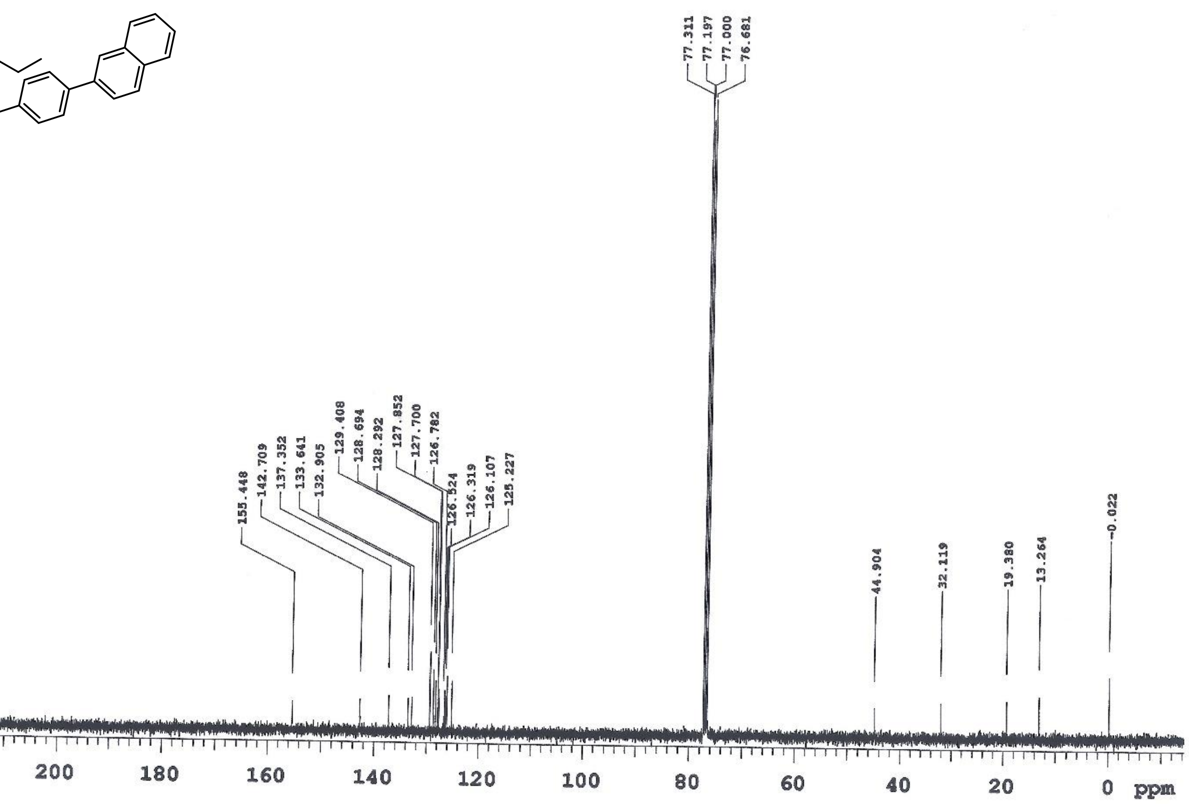

Data file exp

Plot date 2019-07-16

4-Butyl-3,5-bis[4-(quinolin-3-yl)phenyl]-4H-1,2,4-triazole (**9d**).

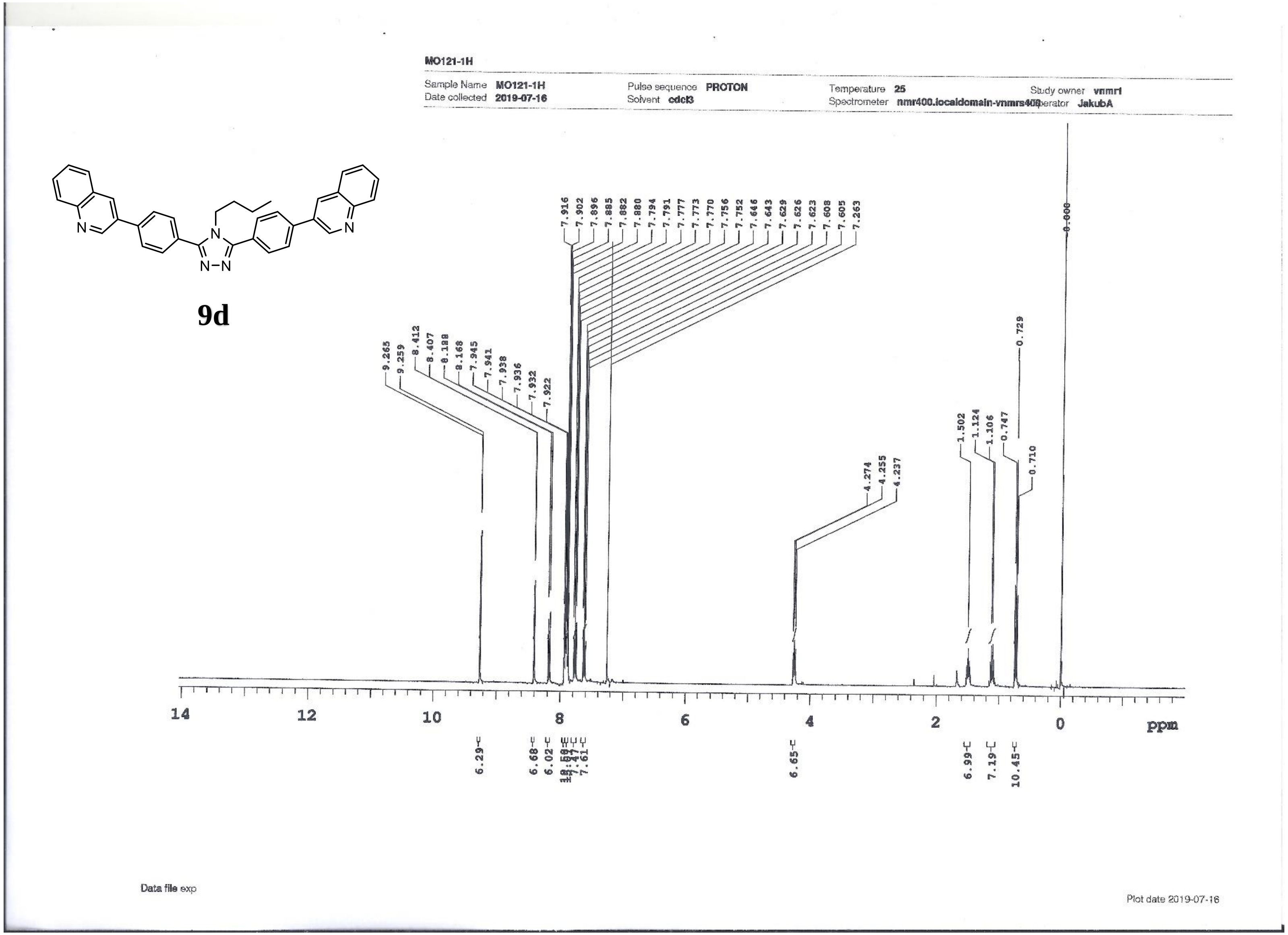

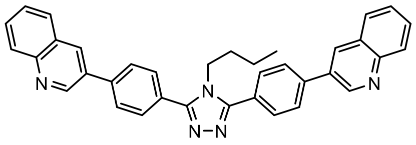

9d

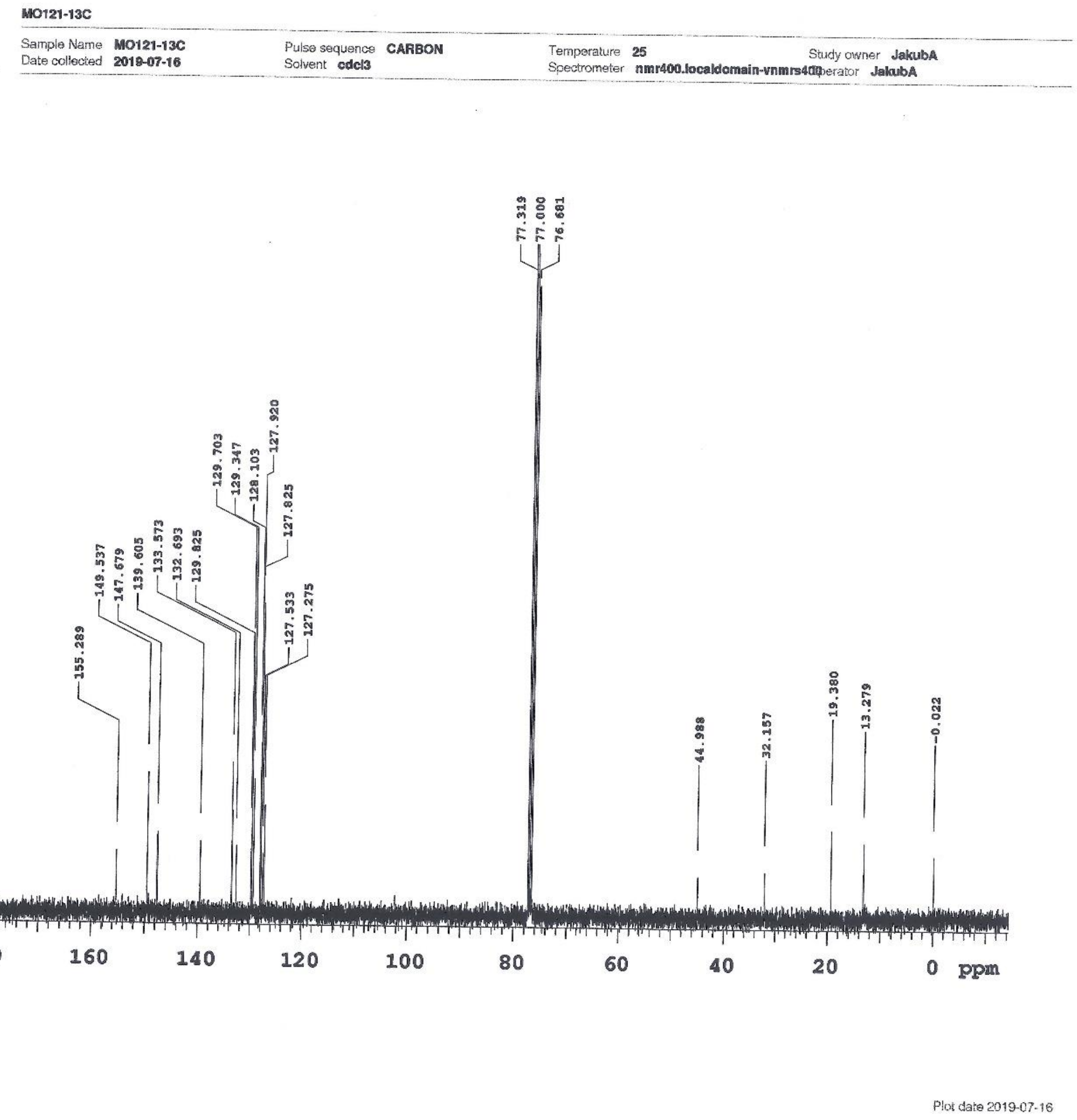
MO121-13C
Sample Name MO121-13C
Date collected 2019-07-16
Pulse sequence CARBON
Solvent cdcl3
Temperature 25
Spectrometer nmr400.localdomain-vnmrs400
Study owner JakubA
Operator JakubA
77.319
77.000
76.681
155.289
149.537
147.679
139.605
133.573
132.693
129.825
129.703
129.347
128.103
127.920
127.825
127.533
127.275
44.988
32.157
19.380
13.279
-0.022
220
200
180
160
140
120
100
80
60
40
20
0 ppm
Data file exp
Plot date 2019-07-16

4-Butyl-3,5-bis[4-(quinolin-6-yl)phenyl]-4H-1,2,4-triazole (**9e**).

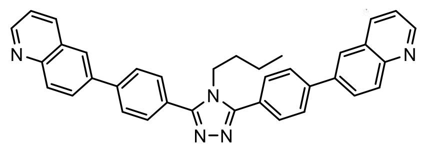

9e

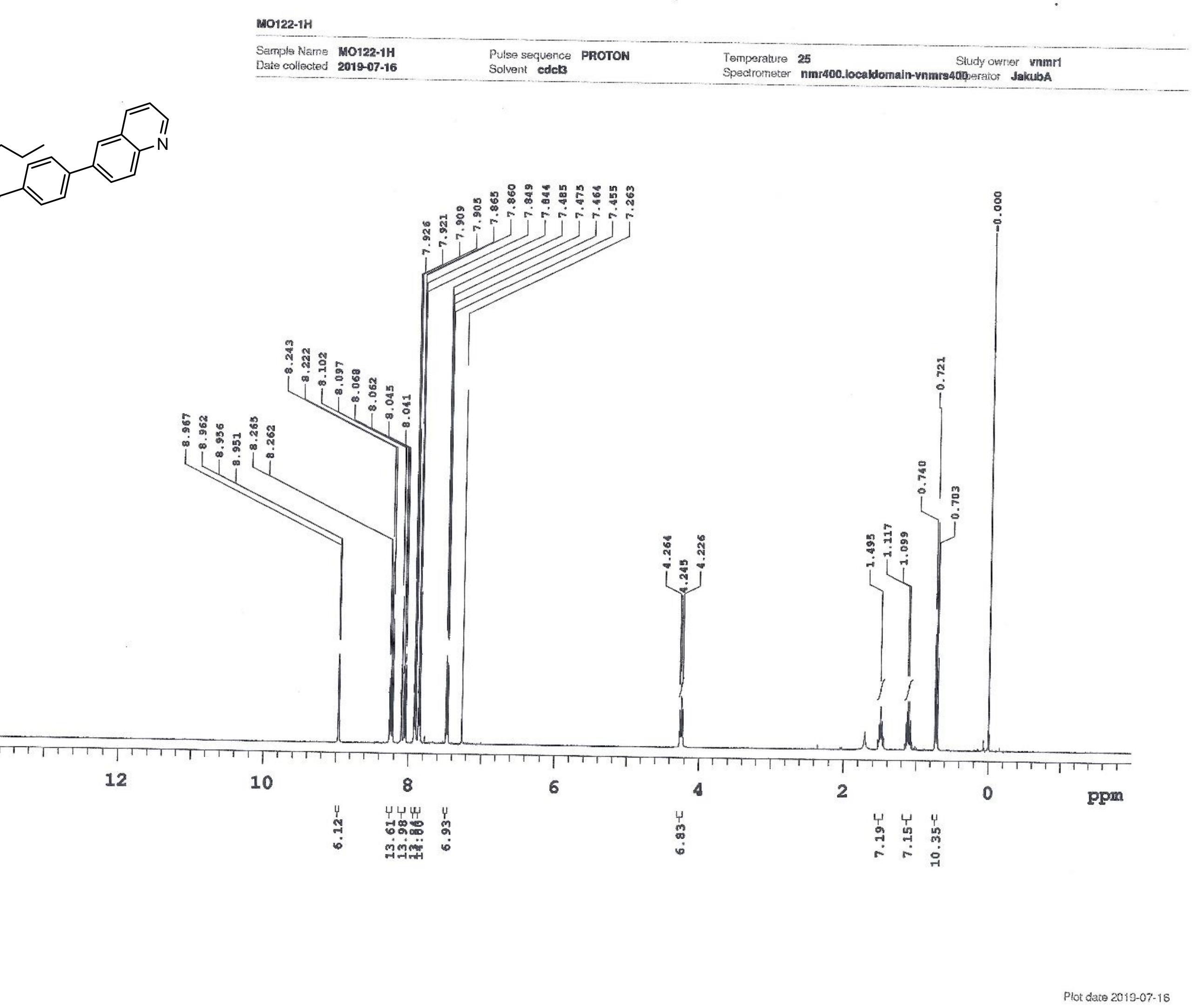

MO122-13C

Sample Name **MO122-13C** Pulse sequence **CARBON** Temperature **25** Study owner **JakubA**
Date collected **2019-07-16** Solvent **cdcl3** Spectrometer **nmr400.localdomain-vnmrs400** Operator **JakubA**

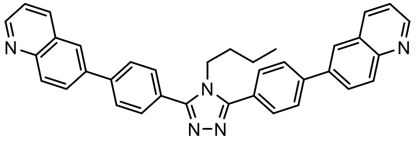

9e

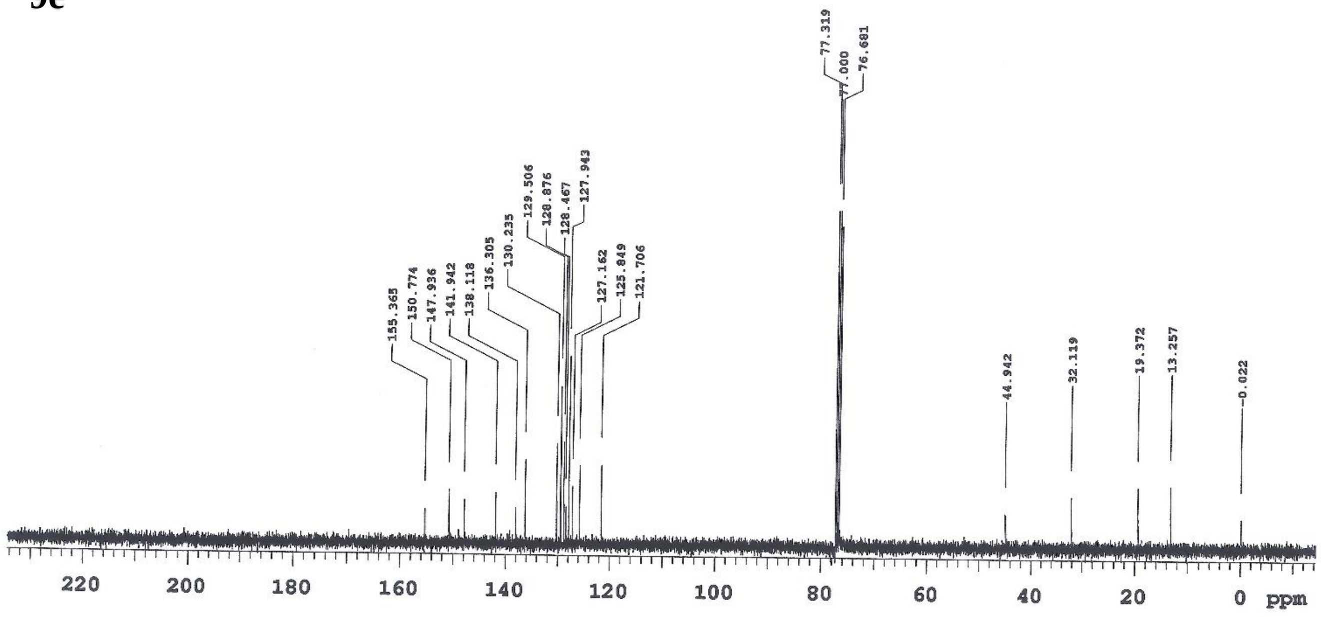

Data file exp

Plot date 2019-07-16

3,5-Bis[4-(dibenzothiophen-4-yl)phenyl]-4-butyl-4H-1,2,4-triazole (**9f**).

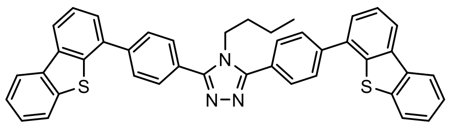

9f

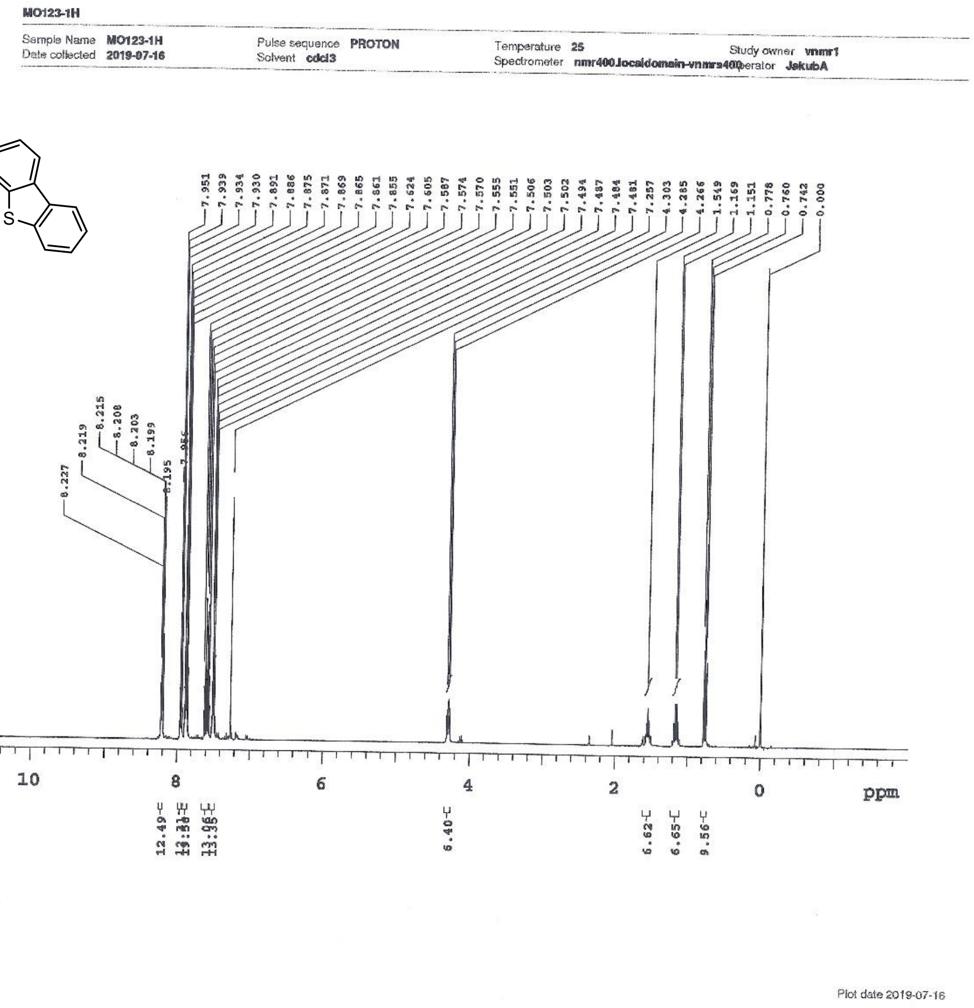

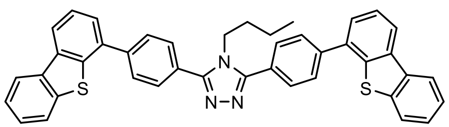

9f

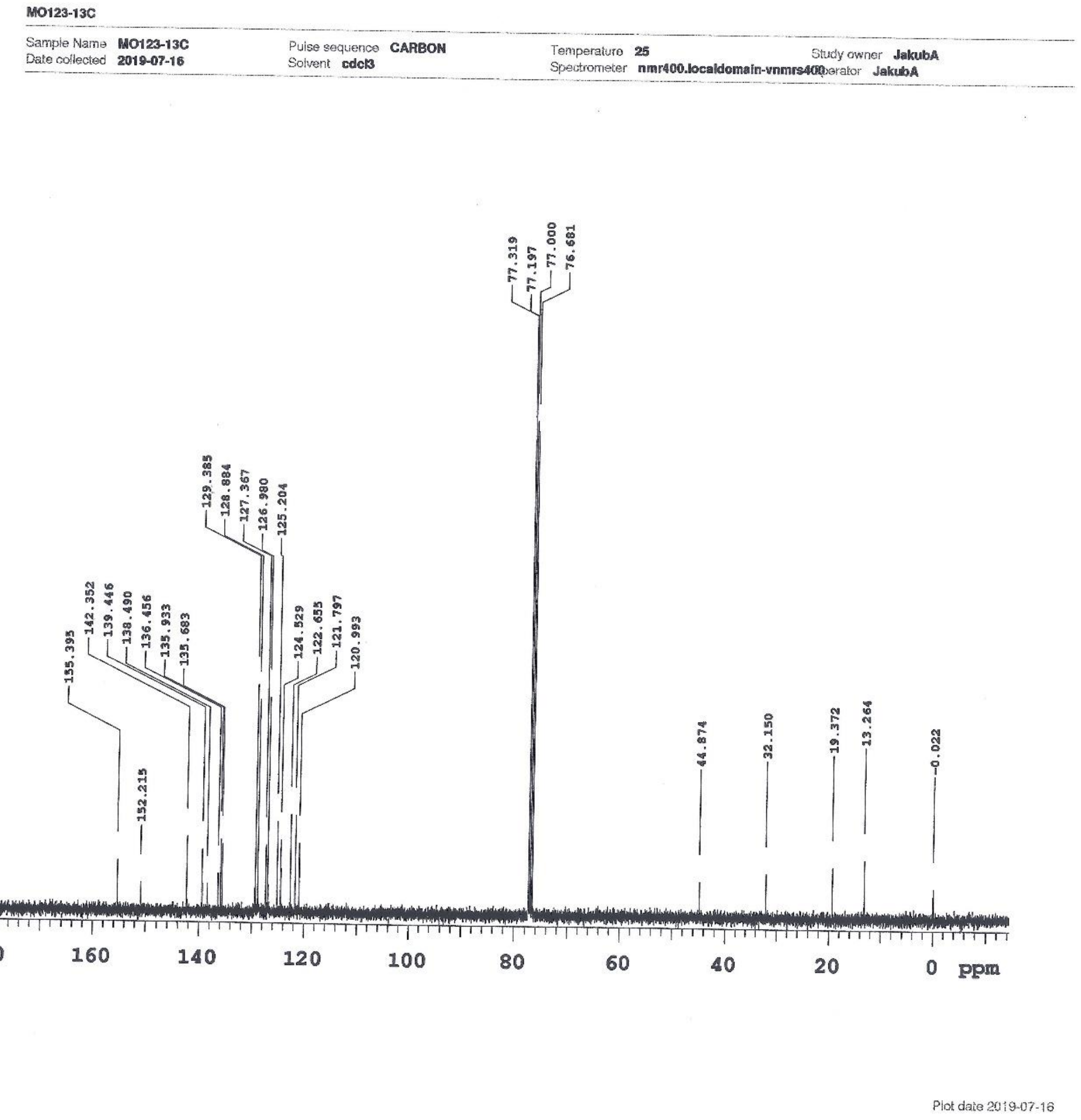

MO123-13C
Sample Name MO123-13C
Date collected 2019-07-16
Pulse sequence CARBON
Solvent cdcl3
Temperature 25
Spectrometer nmr400.localdomain-vnmrs400
Study owner JakubA
Operator JakubA
155.395
152.215
142.352
139.446
138.490
136.456
135.933
135.683
129.385
128.884
127.367
126.980
125.204
124.529
122.655
121.797
120.993
77.319
77.197
77.000
76.681
44.874
32.150
19.372
13.264
-0.022
220
200
180
160
140
120
100
80
60
40
20
0 ppm
Data file exp
Plot date 2019-07-16

3,5-Bis[4-(dibenzofuran-4-yl)phenyl]-4-butyl-4H-1,2,4-triazole (**9g**).

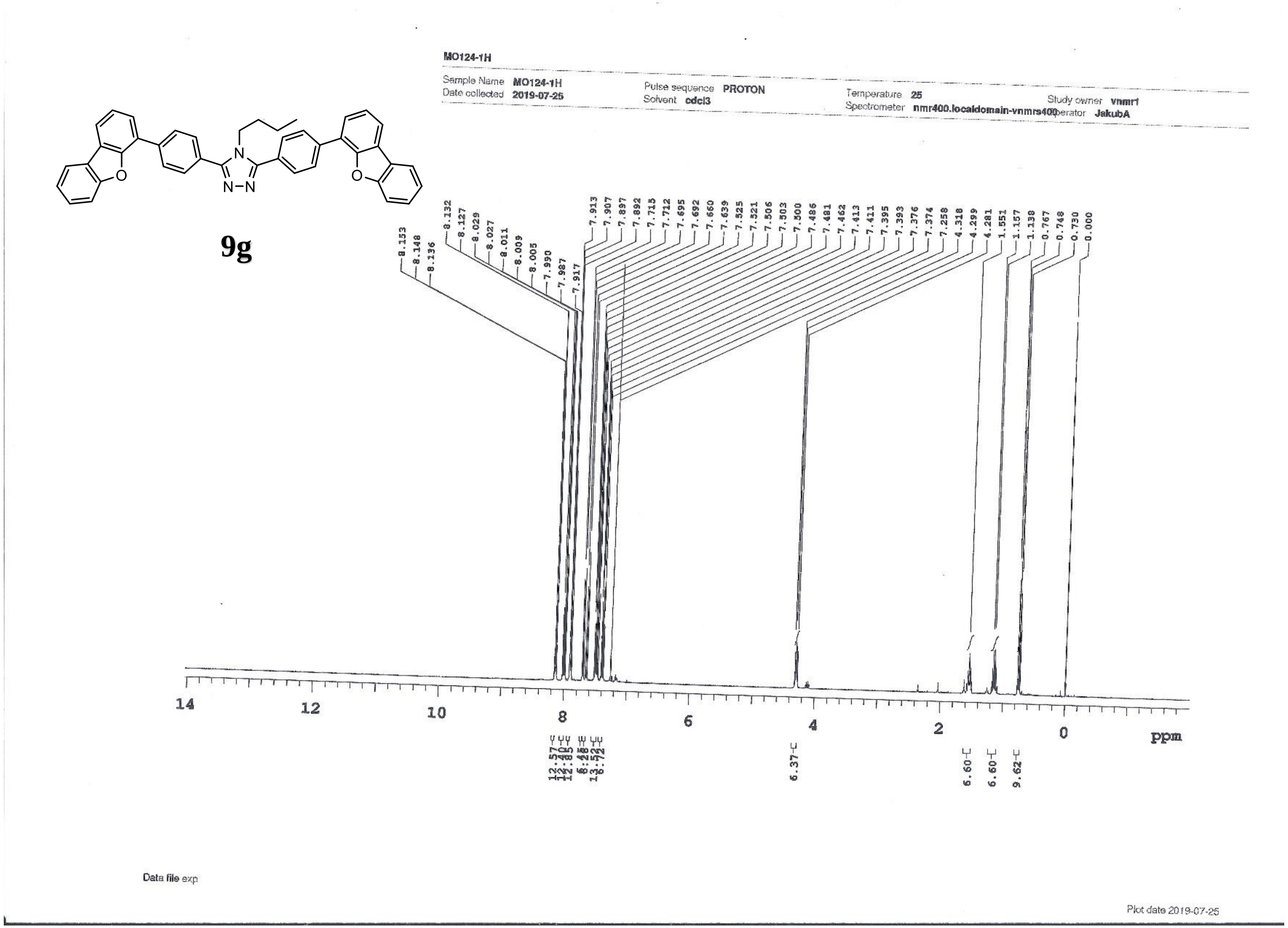

MO124-13C

Sample Name MO124-13C
Date collected 2019-07-25
Pulse sequence CARBON
Solvent cdcl3
Temperature 25
Spectrometer nmr400.localdomain-vnmrs400
Study owner JakubA
Operator JakubA

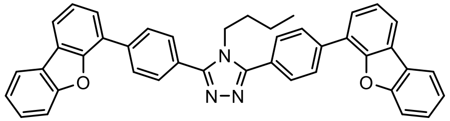

9g

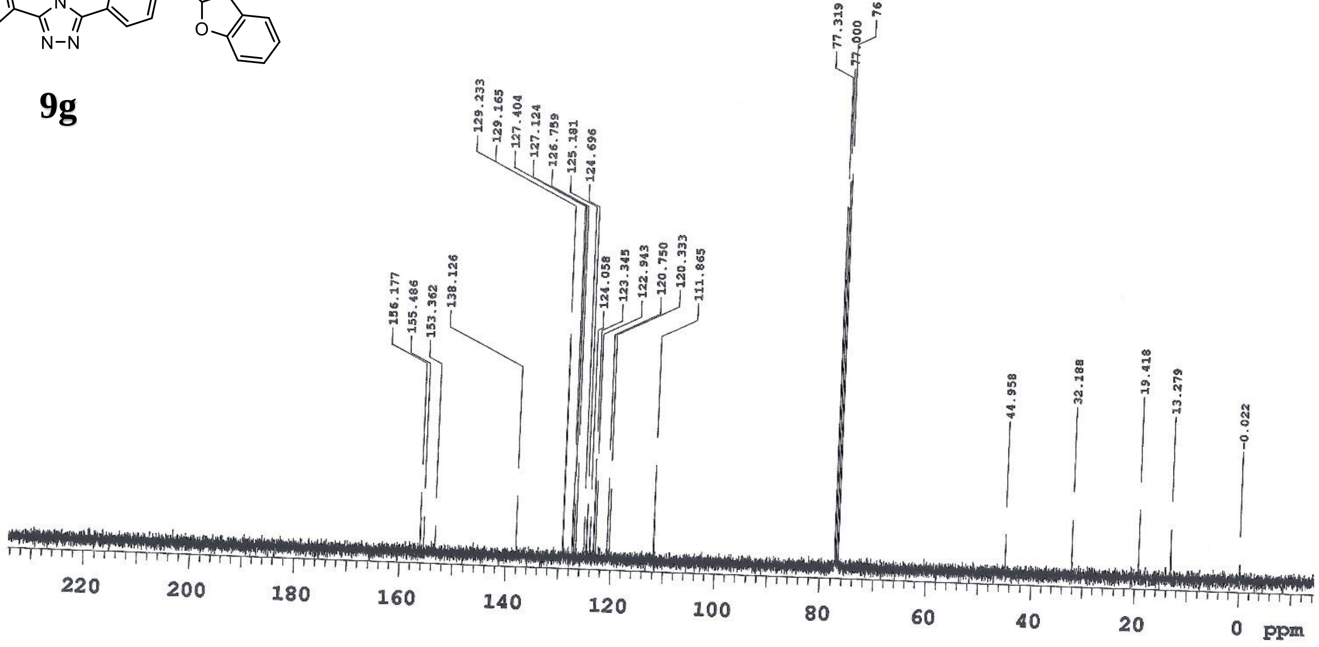

Data file exp

Plot date 2019-07-25

4-Butyl-3,5-bis[4-(9-methyl-9H-carbazol-3-yl)phenyl]-4H-1,2,4-triazole (**9h**).

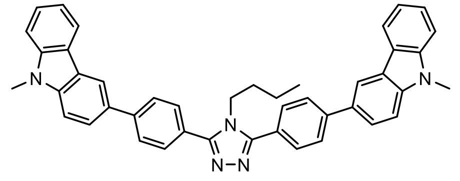

9h

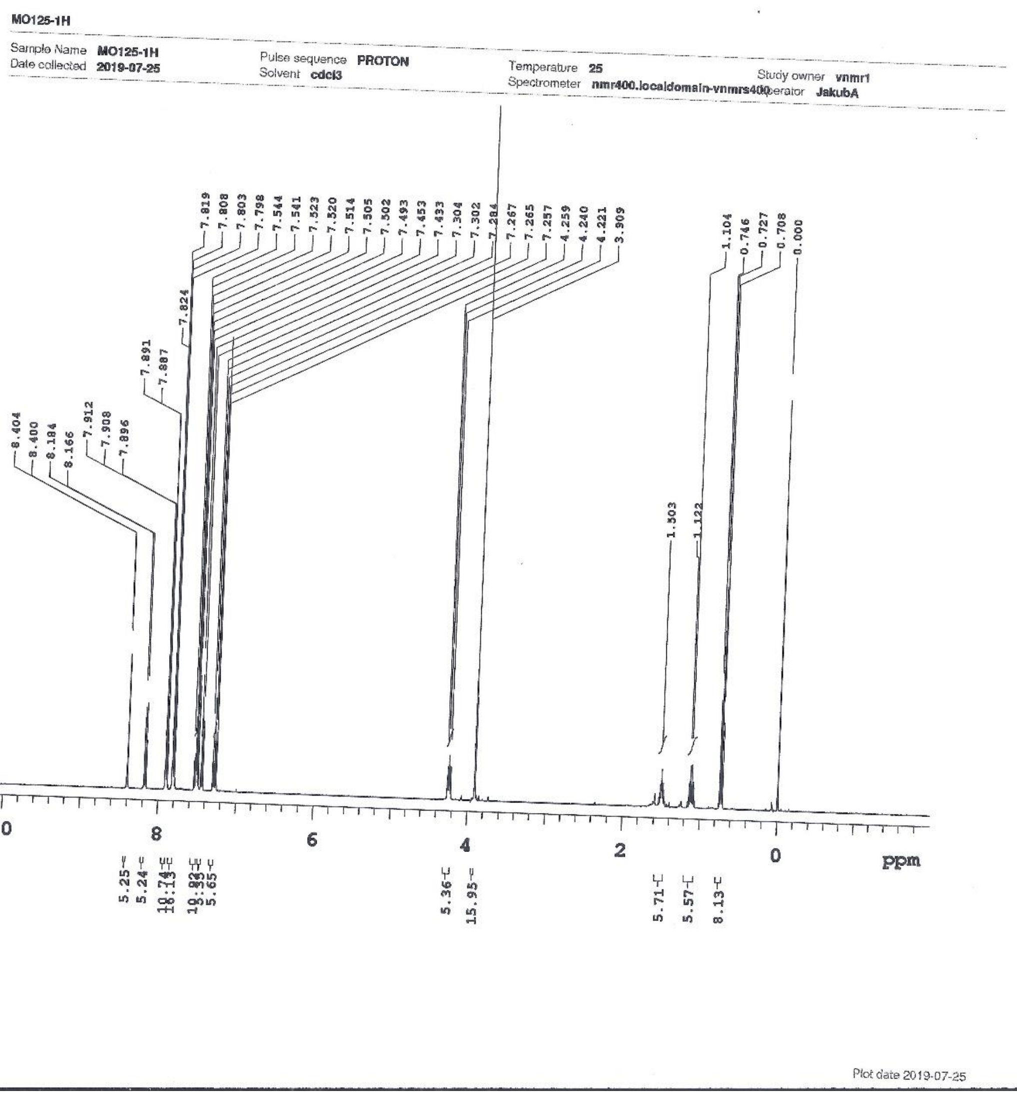

MO125-13C

Sample Name MO125-13C
Date collected 2019-07-25
Pulse sequence CARBON
Solvent cdcl3
Temperature 25
Spectrometer nmr400.localdomain-vnmrs400
Study owner JakubA
Operator JakubA

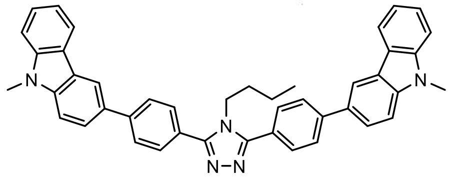

9h

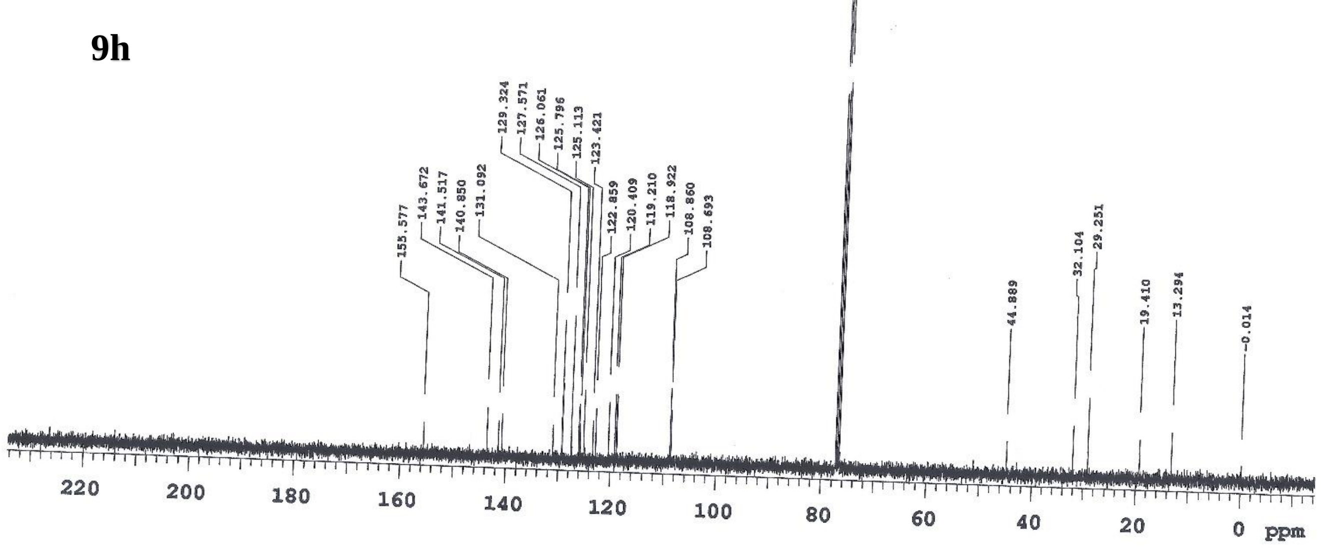

Data file exp

Plot date 2019-07-25

4-Butyl-3,5-bis[4-(9-ethyl-9H-carbazol-3-yl)phenyl]-4H-1,2,4-triazole (**9i**).

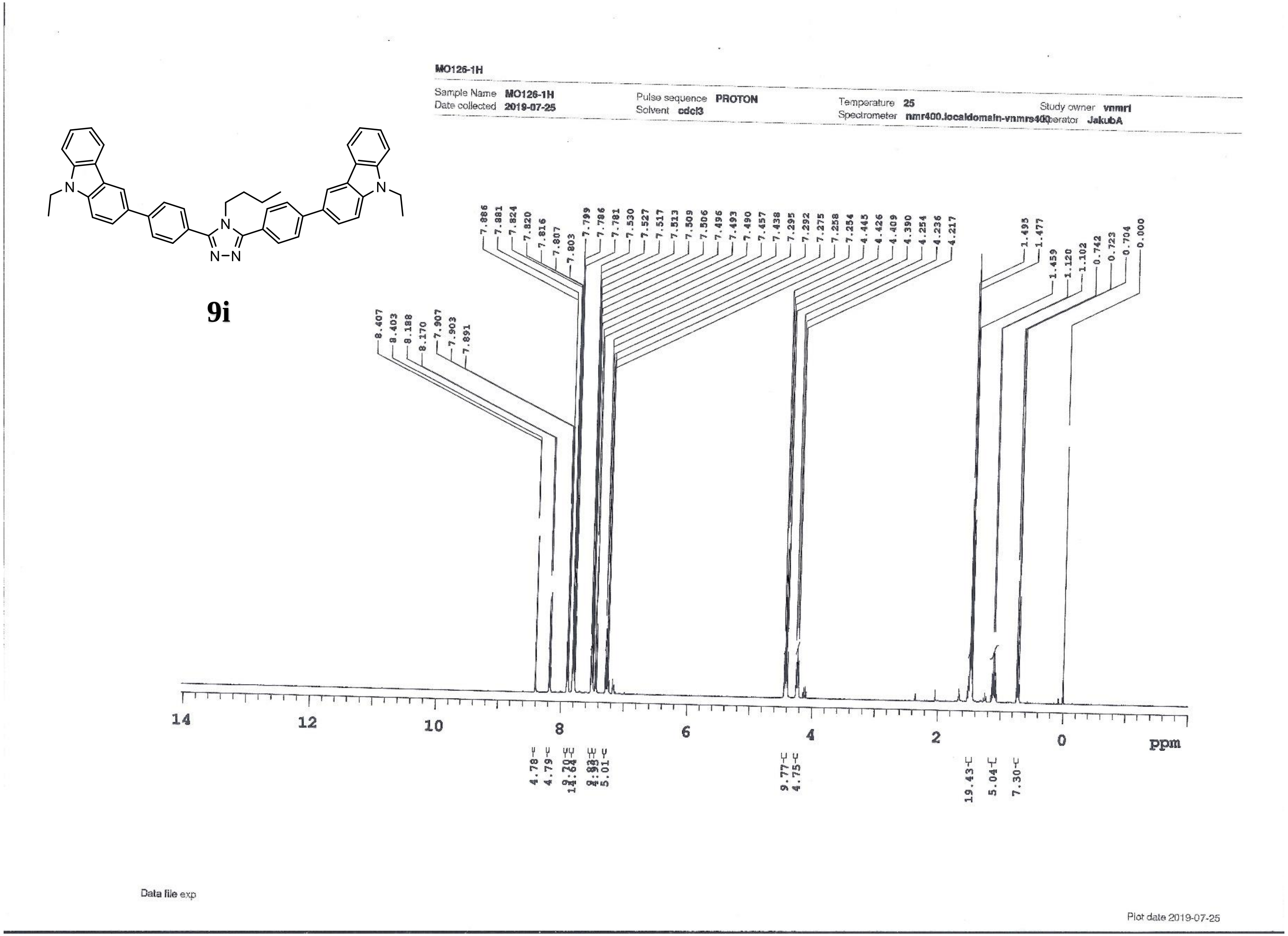

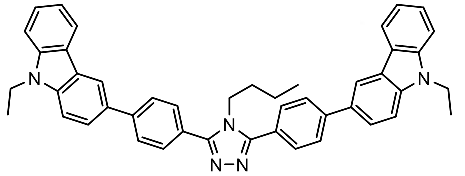

9i

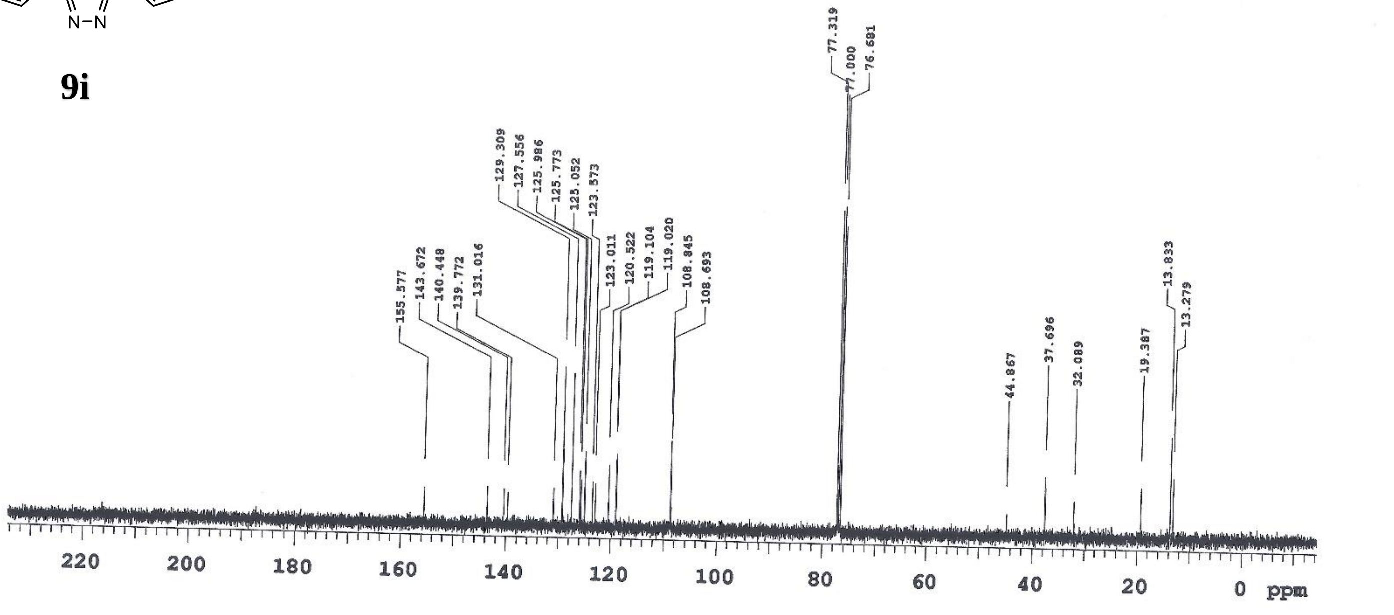
MO126-13C
Sample Name MO126-13C
Date collected 2019-07-25
Pulse sequence CARBON
Solvent cdcl3
Temperature 25
Spectrometer nmr400.localdomain-vnmrs400
Study owner JakubA
Operator JakubA
155.577
143.672
140.448
139.772
131.016
129.309
127.556
125.986
125.773
125.052
123.573
123.011
120.522
119.104
119.020
108.845
108.693
77.319
77.000
76.681
44.867
37.696
32.089
19.387
13.833
13.279
220
200
180
160
140
120
100
80
60
40
20
0 ppm
Data file exp
Plot date 2019-07-25

4-Butyl-3,5-bis[4'-(9H-carbazol-9-yl)biphenyl-4-yl]-4H-1,2,4-triazole (**9j**).

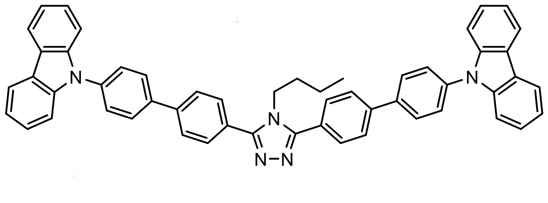

9j

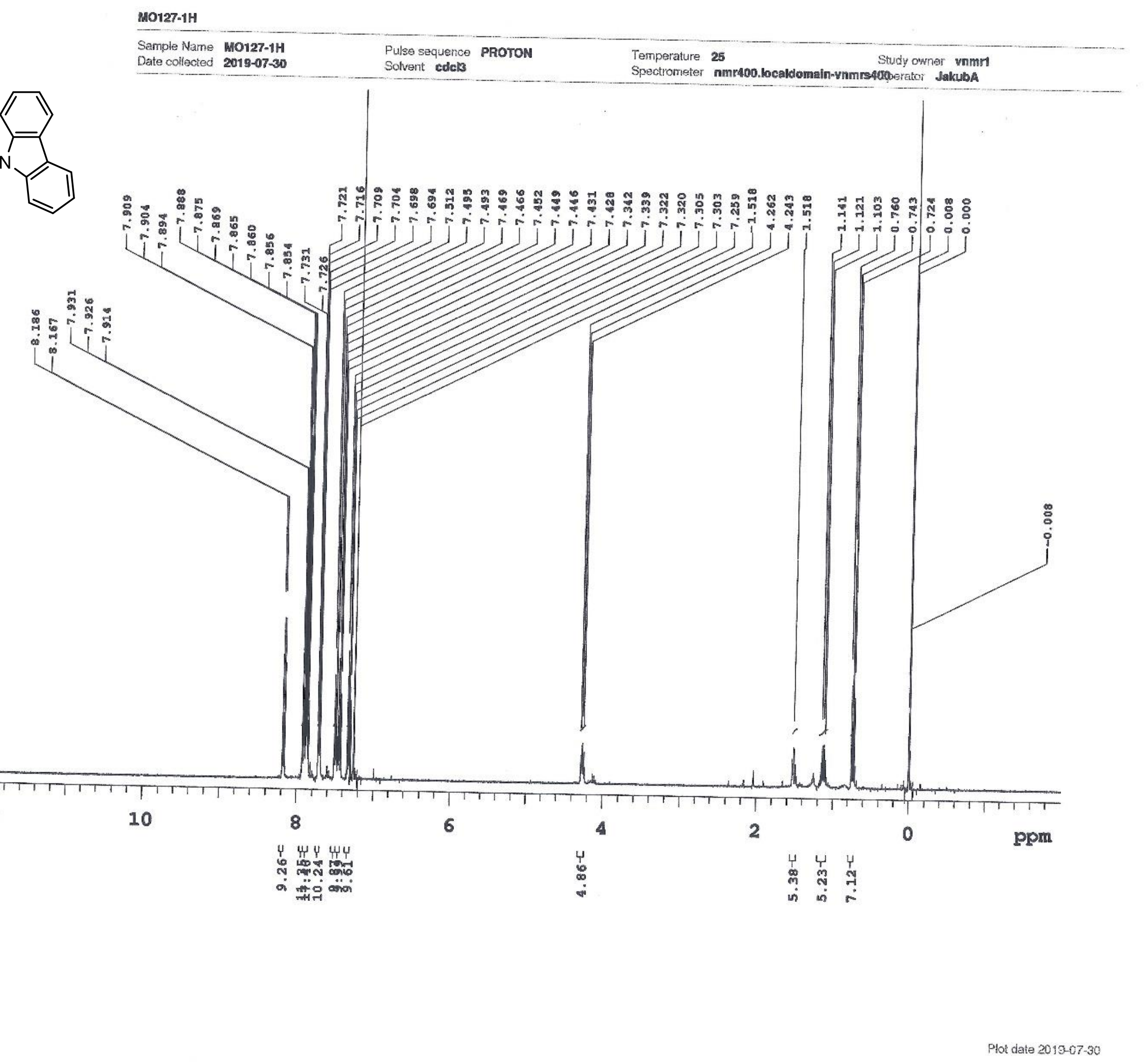

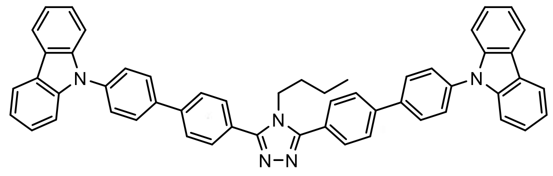
N
N
N–N
N

9j

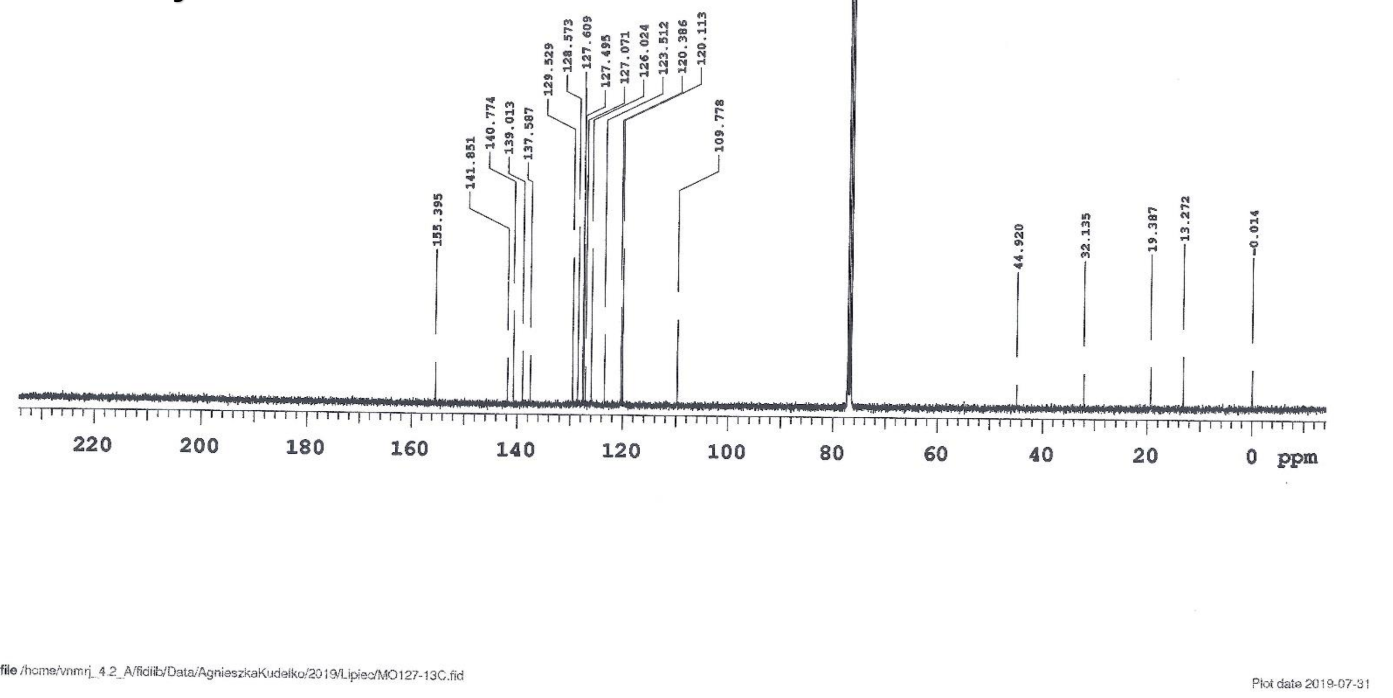
MO127-13C
Sample Name MO127-13C
Date collected 2019-07-30
Pulse sequence CARBON
Solvent cdcl3
Temperature 25
Spectrometer nmr400.localdomain-vnmrs400
Study owner JakubA
Operator JakubA
77.319
77.205
77.000
76.681
155.395
141.851
140.774
139.013
137.587
129.529
128.573
127.609
127.495
127.071
126.024
123.512
120.386
120.113
109.778
44.920
32.135
19.387
13.272
-0.014
220
200
180
160
140
120
100
80
60
40
20
0 ppm
Data file /home/vnmrj_4.2_A/fidlib/Data/AgnieszkaKudelko/2019/Lipiec/MO127-13C.fid
Plot date 2019-07-31

4-Butyl-3,5-bis[4-(thiantren-1-yl)phenyl]-4H-1,2,4-triazole (**9k**).

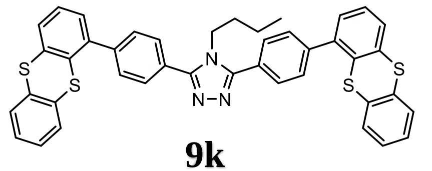

9k

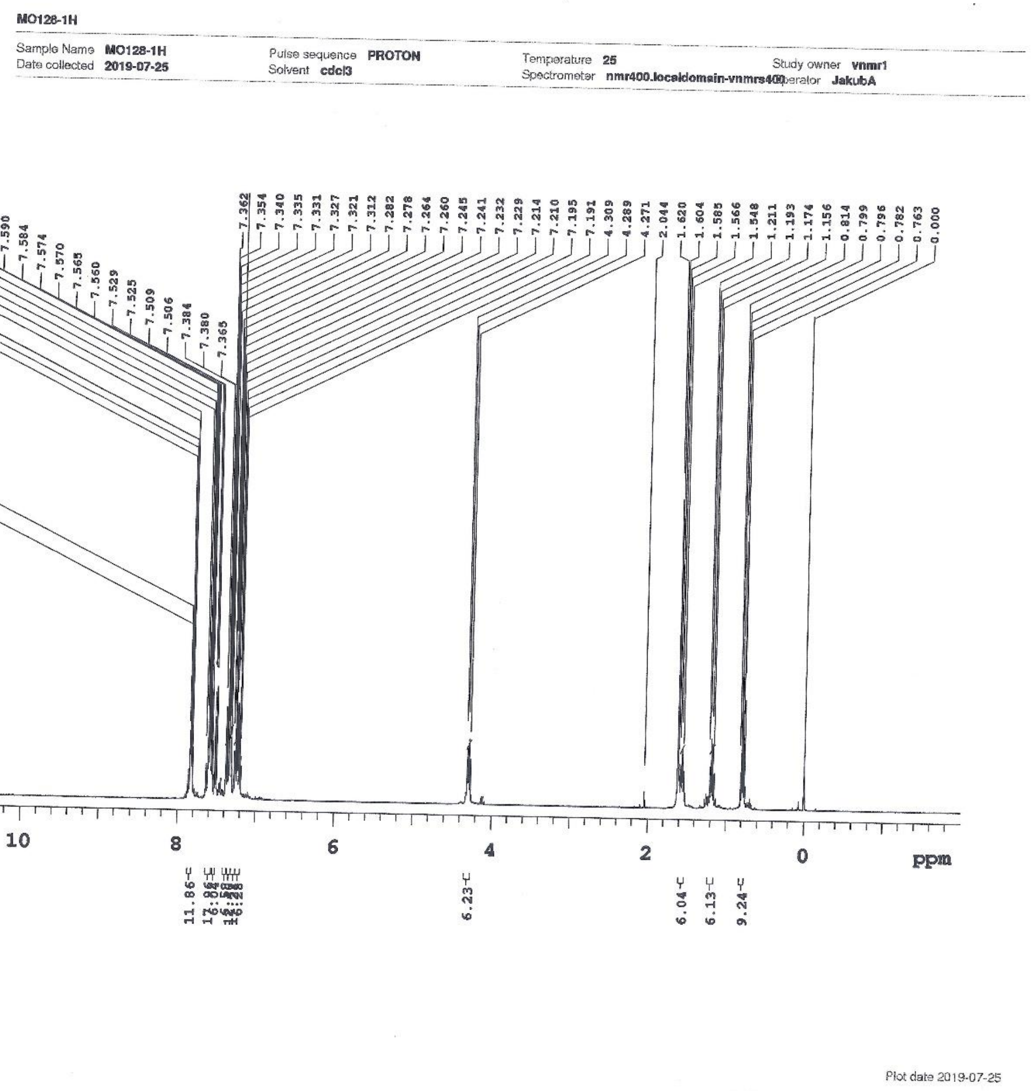

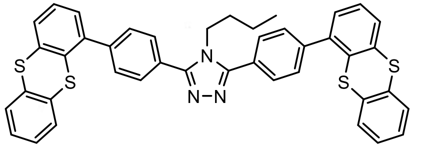

9k

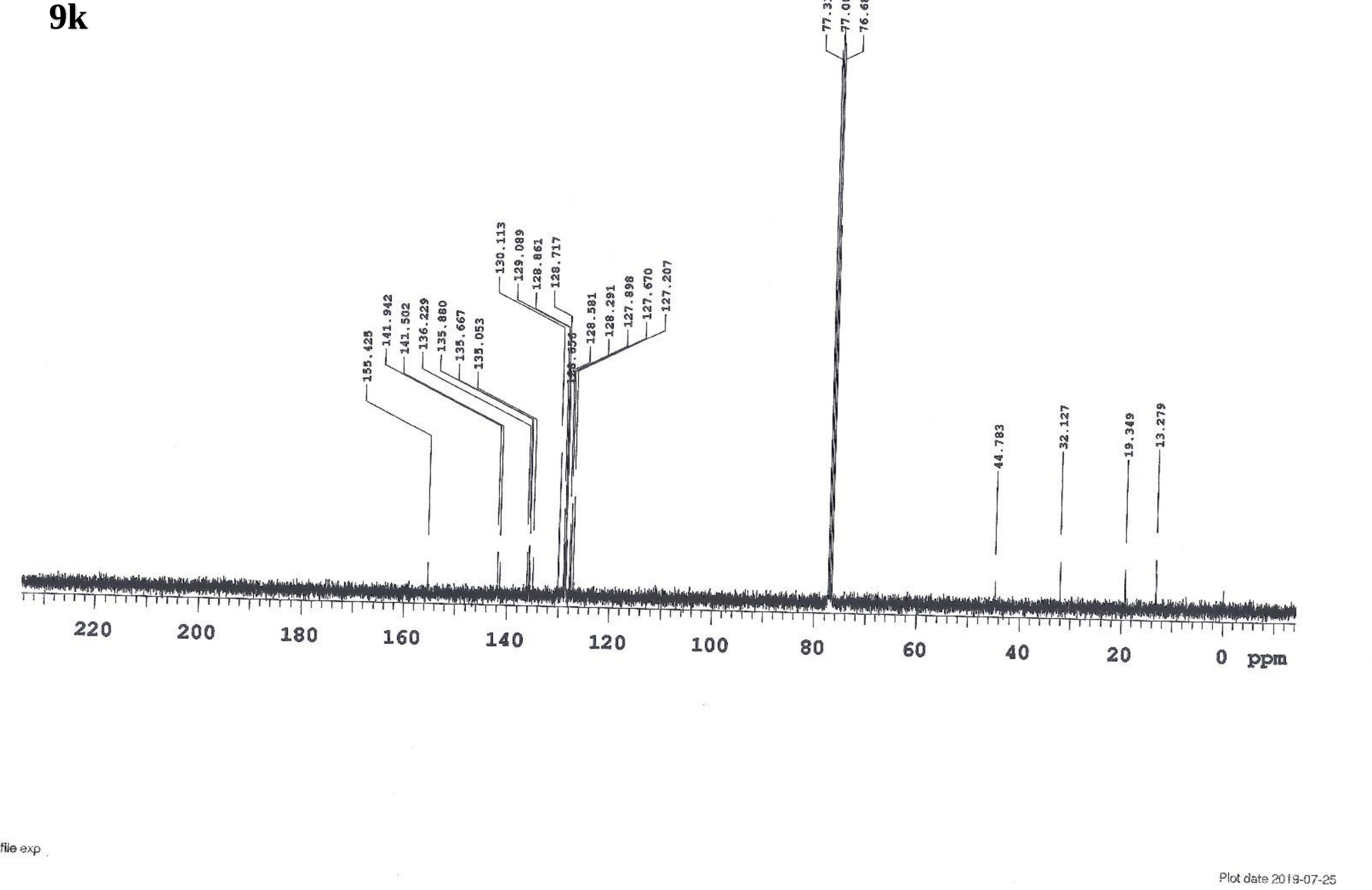
MO128-13C
Sample Name MO128-13C
Date collected 2019-07-25
Pulse sequence CARBON
Solvent cdcl3
Temperature 25
Spectrometer nmr400.localdomain-vnmrs400
Study owner JakubA
Operator JakubA
155.425
141.942
141.502
136.229
135.880
135.667
135.053
130.113
129.089
128.861
128.717
128.581
128.291
127.898
127.670
127.207
77.319
77.000
76.681
44.783
32.127
19.349
13.279
220
200
180
160
140
120
100
80
60
40
20
0 ppm
Data file exp
Plot date 2019-07-25

4-Hexyl-3,5-bis[4′-(N,N-diphenylamino)biphenyl-4-yl]-4H-1,2,4-triazole (**10a**).

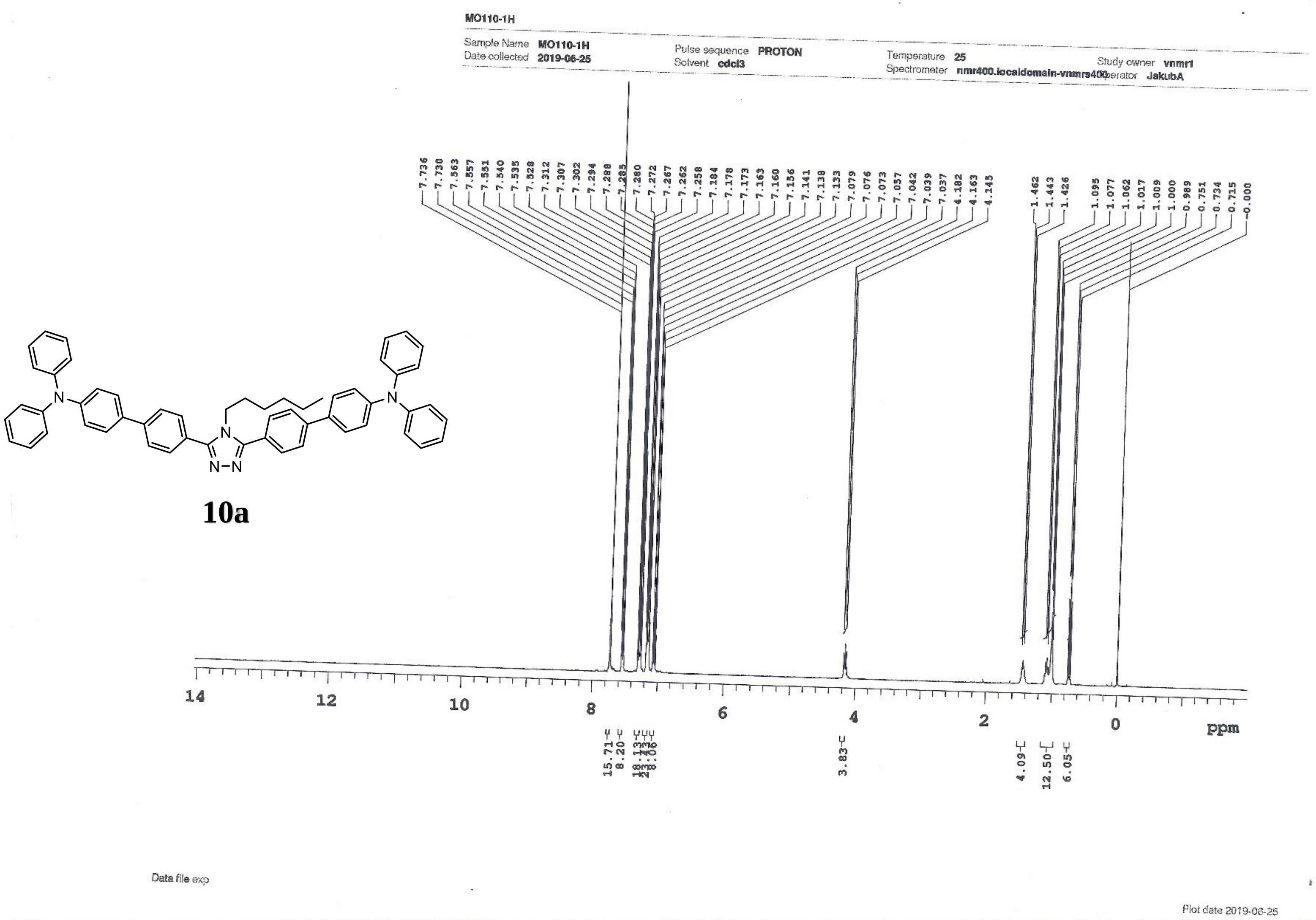

10a

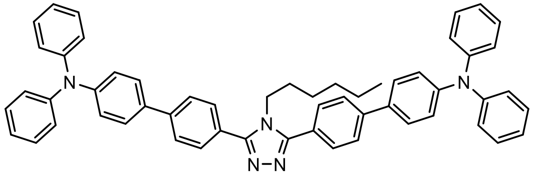

10a

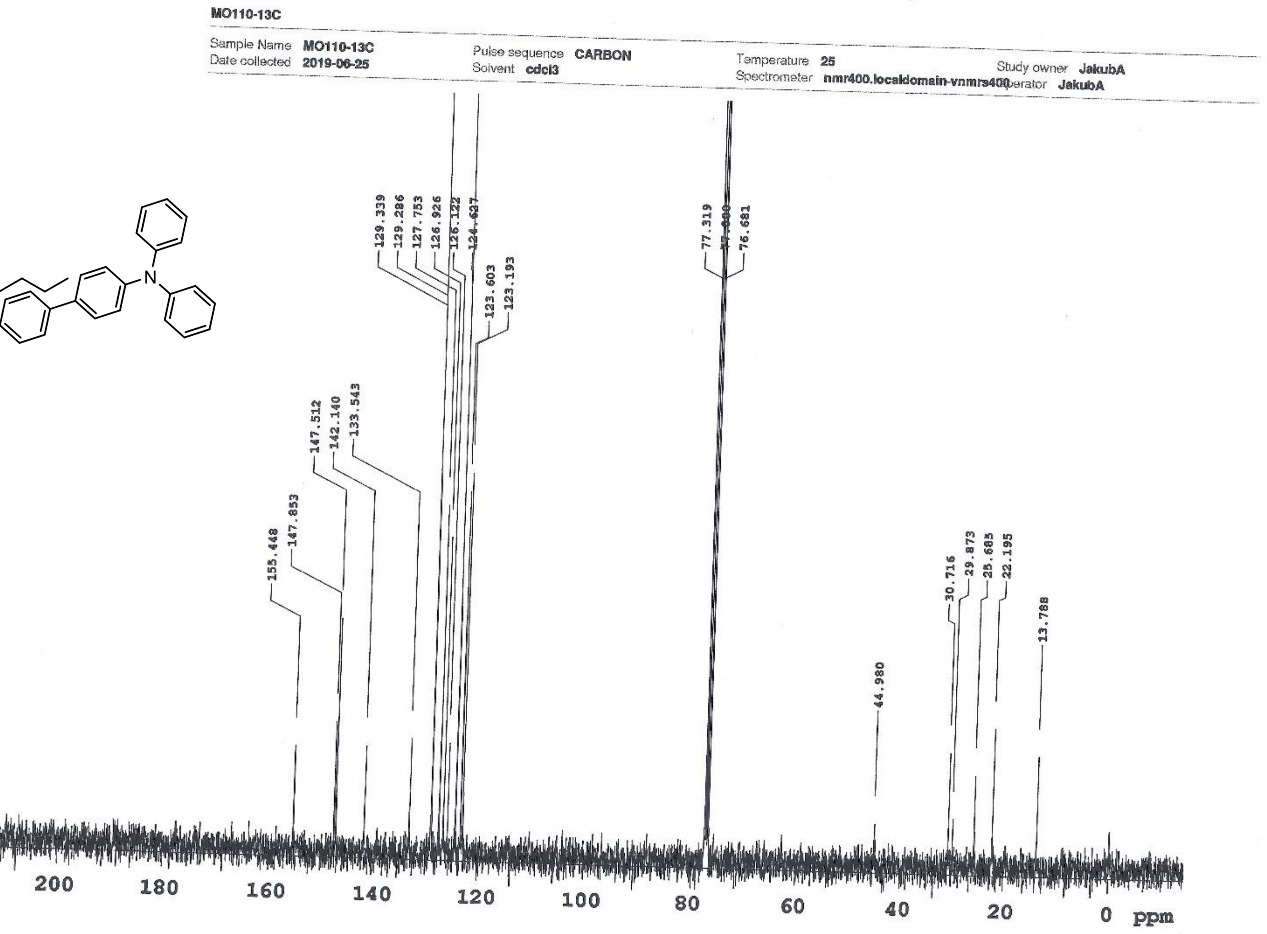

Data file exp

Plot date 2019-06-25

2. HRMS spectra

4-Ethyl-3,5-bis[4′-(N,N-diphenylamino)biphenyl-4-yl]-4H-1,2,4-triazole (**7a**).

Multiple Mass Analysis: 2 mass(es) processed
Tolerance = 100.0 mDa / DBE: min = -10.0, max = 50.0
Element prediction: Off
Number of isotope peaks used for i-FIT = 2
Monoisotopic Mass, Even Electron Ions
186 formula(e) evaluated with 38 results within limits (up to 5 closest results for each mass)
Elements Used:

Mass	RA	Calc. Mass	mDa	PPM	DBE	Formula	i-FIT	i-FIT Norm	Fit Conf %	C	H	N	O
736.3441	100.00	736.3440	0.1	0.1	34.5	C52 H42 N5	89.8	1.252	28.60	52	42	5	
		736.3328	11.3	15.3	34.5	C53 H42 N3 O	89.7	1.162	31.27	53	42	3	1
		736.3579	-13.8	-18.7	33.5	C55 H46 N O	90.6	2.004	13.48	55	46	1	1
		736.3652	-21.1	-28.7	29.5	C49 H46 N5 O2	93.5	4.924	0.73	49	46	5	2
		736.3216	22.5	30.6	34.5	C54 H42 N O2	89.9	1.350	25.92	54	42	1	2
737.3462	59.22	737.3420	4.2	5.7	33.5	C55 H45 O2	102.7	1.453	23.40	55	45		2
		737.3532	-7.0	-9.5	33.5	C54 H45 N2 O	102.8	1.525	21.76	54	45	2	1
		737.3280	18.2	24.7	34.5	C52 H41 N4 O	102.9	1.634	19.52	52	41	4	1

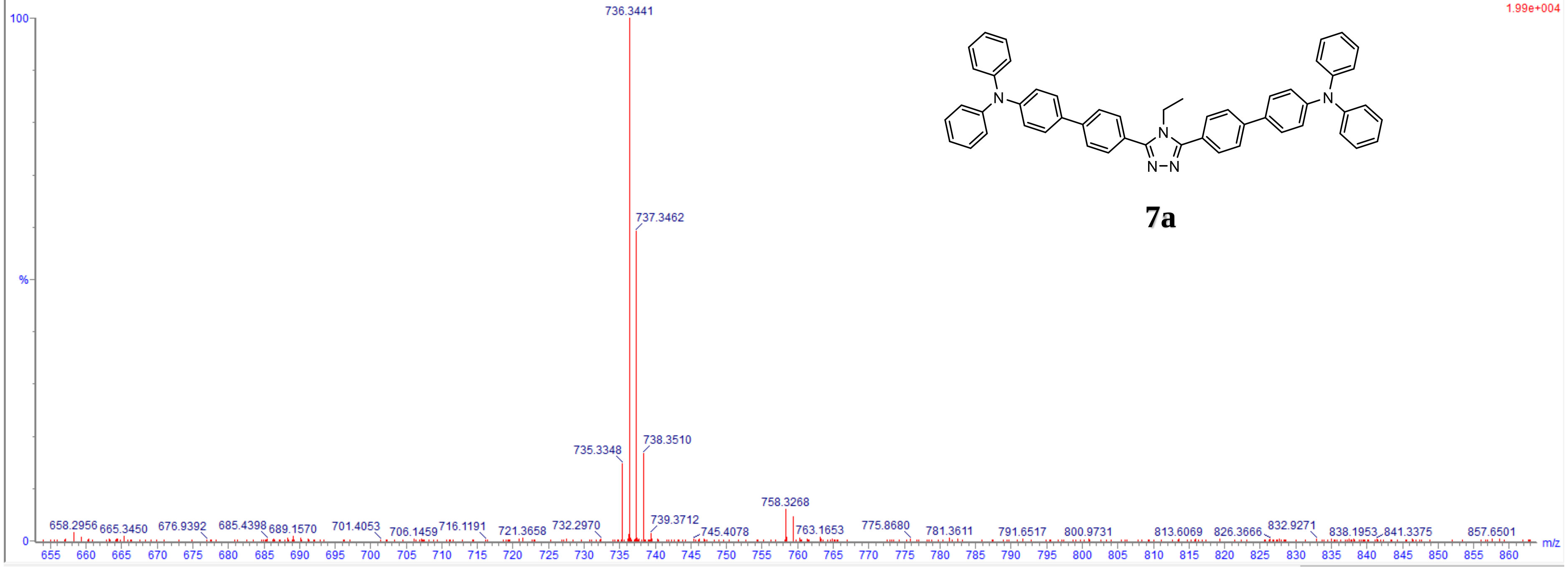

7a

4-Ethyl-3,5-bis[4-(naphthalen-1-yl)phenyl]-4H-1,2,4-triazole (**7b**).

Tolerance = 100.0 mDa / DBE: min = -10.0, max = 50.0
Element prediction: Off
Number of isotope peaks used for i-FIT = 2
Monoisotopic Mass, Even Electron Ions
31 formula(e) evaluated with 7 results within limits (up to 5 closest results for each mass)
Elements Used:

Mass	RA	Calc. Mass	mDa	PPM	DBE	Formula	i-FIT	i-FIT Norm	Fit Conf %	C	H	N
502.2281	100.00	502.2283	-0.2	-0.4	24.5	C36 H28 N3	409.4	8.228	0.03	36	28	3
		502.2032	24.9	49.6	25.5	C34 H24 N5	406.8	5.678	0.34	34	24	5
		502.2535	-25.4	-50.6	23.5	C38 H32 N	411.1	9.967	0.00	38	32	1
		502.1596	68.5	136.4	30.5	C39 H20 N	413.3	12.112	0.00	39	20	1
		502.2971	-69.0	-137.4	18.5	C33 H36 N5	401.1	0.004	99.63	33	36	5

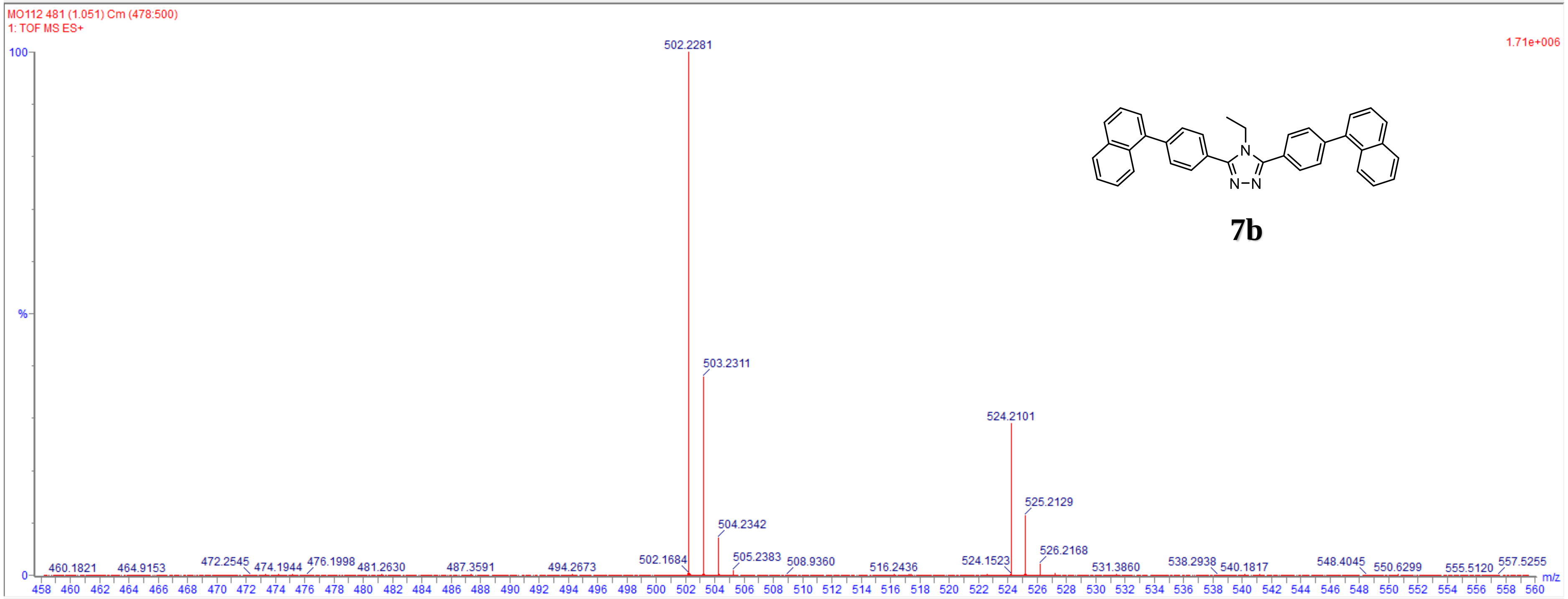

4-Ethyl-3,5-bis[4-(naphthalen-2-yl)phenyl]-4H-1,2,4-triazole (**7c**).

Tolerance = 100.0 mDa / DBE: min = -10.0, max = 50.0
Element prediction: Off
Number of isotope peaks used for i-FIT = 2
Monoisotopic Mass, Even Electron Ions
31 formula(e) evaluated with 7 results within limits (up to 5 closest results for each mass)
Elements Used:

Mass	RA	Calc. Mass	mDa	PPM	DBE	Formula	i-FIT	i-FIT Norm	Fit Conf %	C	H	N
502.2287	100.00	502.2283	0.4	0.8	24.5	C36 H28 N3	313.4	5.549	0.39	36	28	3
		502.2535	-24.8	-49.4	23.5	C38 H32 N	315.6	7.754	0.04	38	32	1
		502.2032	25.5	50.8	25.5	C34 H24 N5	307.9	0.010	98.97	34	24	5
		502.2971	-68.4	-136.2	18.5	C33 H36 N5	313.0	5.123	0.60	33	36	5
		502.1596	69.1	137.6	30.5	C39 H20 N	317.9	10.073	0.00	39	20	1

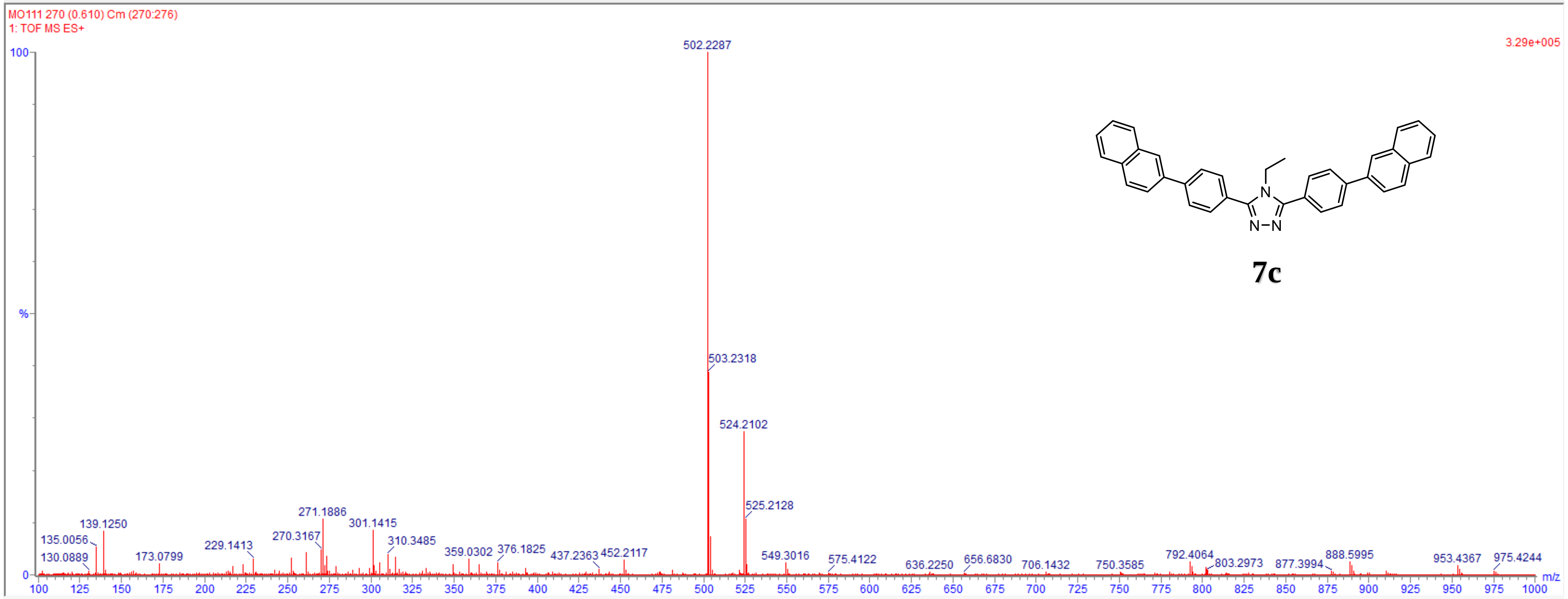

4-Ethyl-3,5-bis[4-(quinolin-3-yl)phenyl]-4H-1,2,4-triazole (**7d**).

Tolerance = 100.0 mDa / DBE: min = -10.0, max = 50.0
Element prediction: Off
Number of isotope peaks used for i-FIT = 2
Monoisotopic Mass, Even Electron Ions
31 formula(e) evaluated with 7 results within limits (up to 5 closest results for each mass)
Elements Used:

Mass	RA	Calc. Mass	mDa	PPM	DBE	Formula	i-FIT	i-FIT Norm	Fit Conf %	C	H	N
504.2188	100.00	504.2188	0.0	0.0	24.5	C34 H26 N5	293.5	0.302	73.92	34	26	5
		504.2440	-25.2	-50.0	23.5	C36 H30 N3	295.0	1.783	16.82	36	30	3
		504.1752	43.6	86.5	29.5	C39 H22 N	296.6	3.457	3.15	39	22	1
		504.2691	-50.3	-99.8	22.5	C38 H34 N	296.5	3.332	3.57	38	34	1
		504.1501	68.7	136.3	30.5	C37 H18 N3	296.9	3.674	2.54	37	18	3

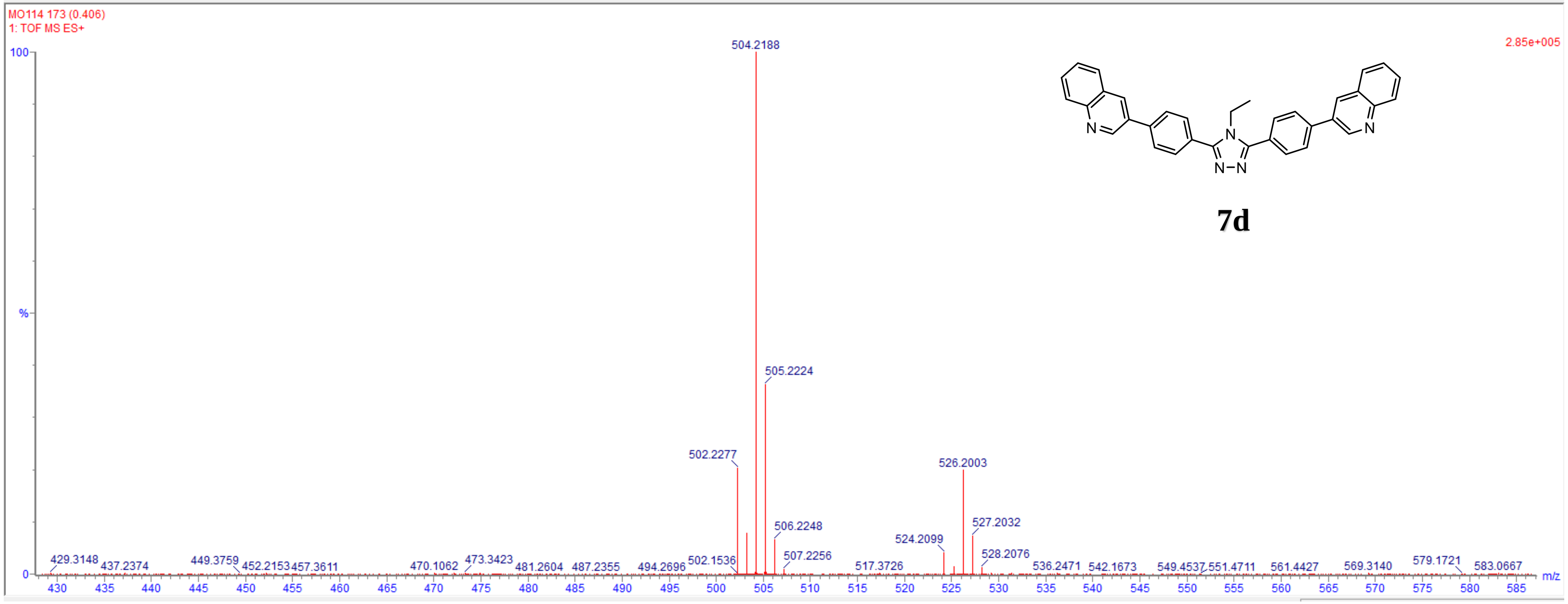

4-Ethyl-3,5-bis[4-(quinolin-6-yl)phenyl]-4H-1,2,4-triazole (**7e**).

Tolerance = 100.0 mDa / DBE: min = -10.0, max = 50.0
Element prediction: Off
Number of isotope peaks used for i-FIT = 2
Monoisotopic Mass, Even Electron Ions
31 formula(e) evaluated with 7 results within limits (up to 5 closest results for each mass)
Elements Used:

Mass	RA	Calc. Mass	mDa	PPM	DBE	Formula	i-FIT	i-FIT Norm	Fit Conf %	C	H	N
504.2189	100.00	504.2188	0.1	0.2	24.5	C34 H26 N5	479.8	0.373	68.87	34	26	5
		504.2440	-25.1	-49.8	23.5	C36 H30 N3	481.1	1.616	19.86	36	30	3
		504.1752	43.7	86.7	29.5	C39 H22 N	482.7	3.246	3.89	39	22	1
		504.2691	-50.2	-99.6	22.5	C38 H34 N	482.5	3.096	4.52	38	34	1
		504.1501	68.8	136.4	30.5	C37 H18 N3	483.0	3.555	2.86	37	18	3

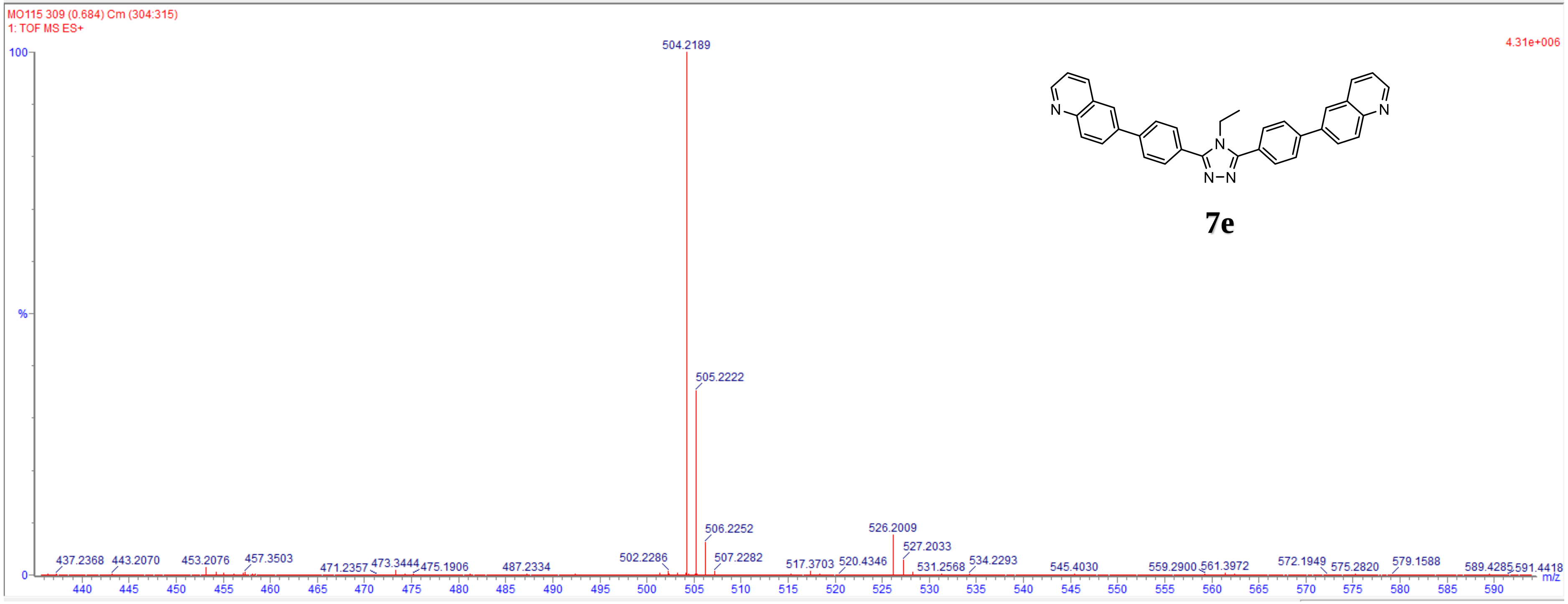

3,5-Bis[4-(dibenzothiophen-4-yl)phenyl]-4-ethyl-4H-1,2,4-triazole (**7f**).

Tolerance = 100.0 mDa / DBE: min = -10.0, max = 50.0
Element prediction: Off
Number of isotope peaks used for i-FIT = 2
Monoisotopic Mass, Even Electron Ions
104 formula(e) evaluated with 23 results within limits (up to 5 closest results for each mass)
Elements Used:

Mass	RA	Calc. Mass	mDa	PPM	DBE	Formula	i-FIT	i-FIT Norm	Fit Conf %	C	H	N	S
614.1727	100.00	614.1725	0.2	0.3	28.5	C40 H28 N3 S2	396.5	2.108	12.15	40	28	3	2
		614.1758	-3.1	-5.0	23.5	C37 H32 N3 S3	394.6	0.156	85.57	37	32	3	3
		614.1691	3.6	5.9	33.5	C43 H24 N3 S	399.2	4.762	0.85	43	24	3	1
		614.1792	-6.5	-10.6	18.5	C34 H36 N3 S4	398.8	4.411	1.21	34	36	3	4
		614.1657	7.0	11.4	38.5	C46 H20 N3	400.5	6.122	0.22	46	20	3	

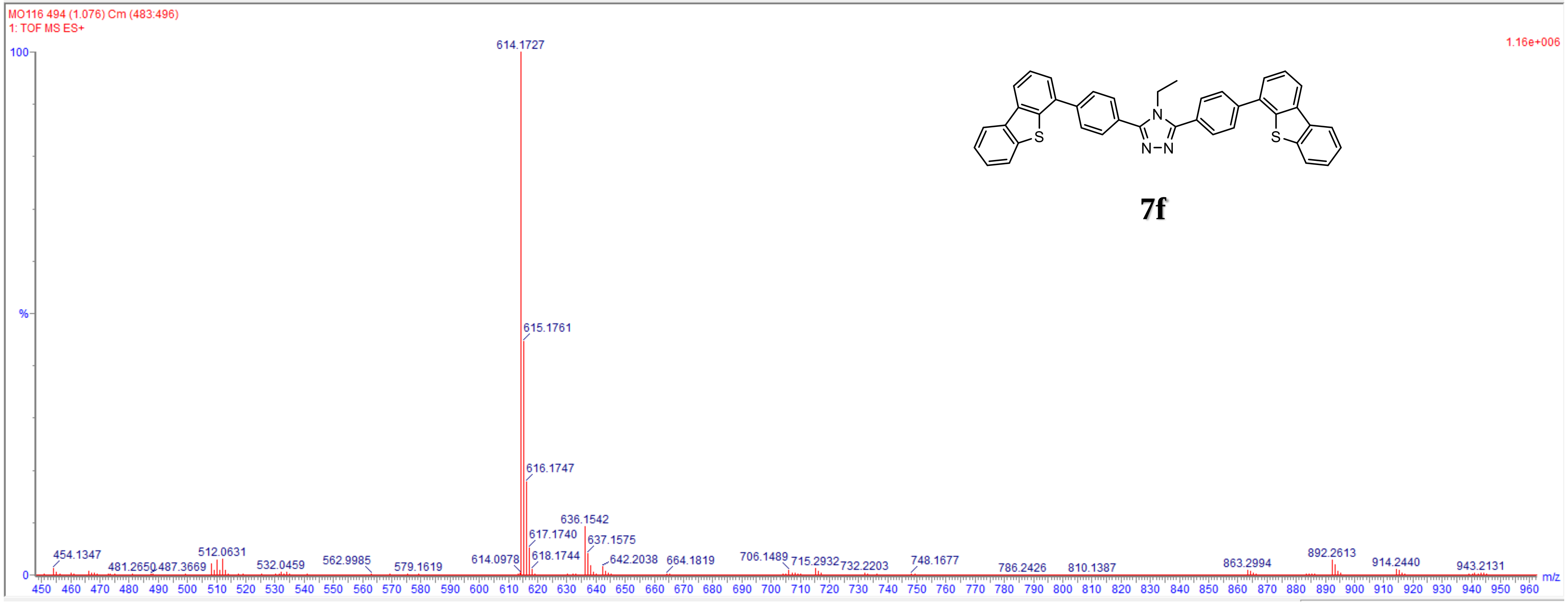

3,5-Bis[4-(dibenzofuran-4-yl)phenyl]-4-ethyl-4H-1,2,4-triazole (**7g**).

Tolerance = 100.0 mDa / DBE: min = -10.0, max = 50.0
Element prediction: Off
Number of isotope peaks used for i-FIT = 2
Monoisotopic Mass, Even Electron Ions
93 formula(e) evaluated with 20 results within limits (up to 5 closest results for each mass)
Elements Used:

Mass	RA	Calc. Mass	mDa	PPM	DBE	Formula	i-FIT	i-FIT Norm	Fit Conf %	C	H	N	O
582.2183	100.00	582.2182	0.1	0.2	28.5	C40 H28 N3 O2	366.1	1.155	31.51	40	28	3	2
		582.2222	-3.9	-6.7	32.5	C45 H28 N	369.7	4.760	0.86	45	28	1	
		582.2294	-11.1	-19.1	28.5	C39 H28 N5 O	365.4	0.482	61.73	39	28	5	1
		582.1970	21.3	36.6	33.5	C43 H24 N3	369.1	4.154	1.57	43	24	3	
		582.2433	-25.0	-42.9	27.5	C42 H32 N O2	368.1	3.140	4.33	42	32	1	2

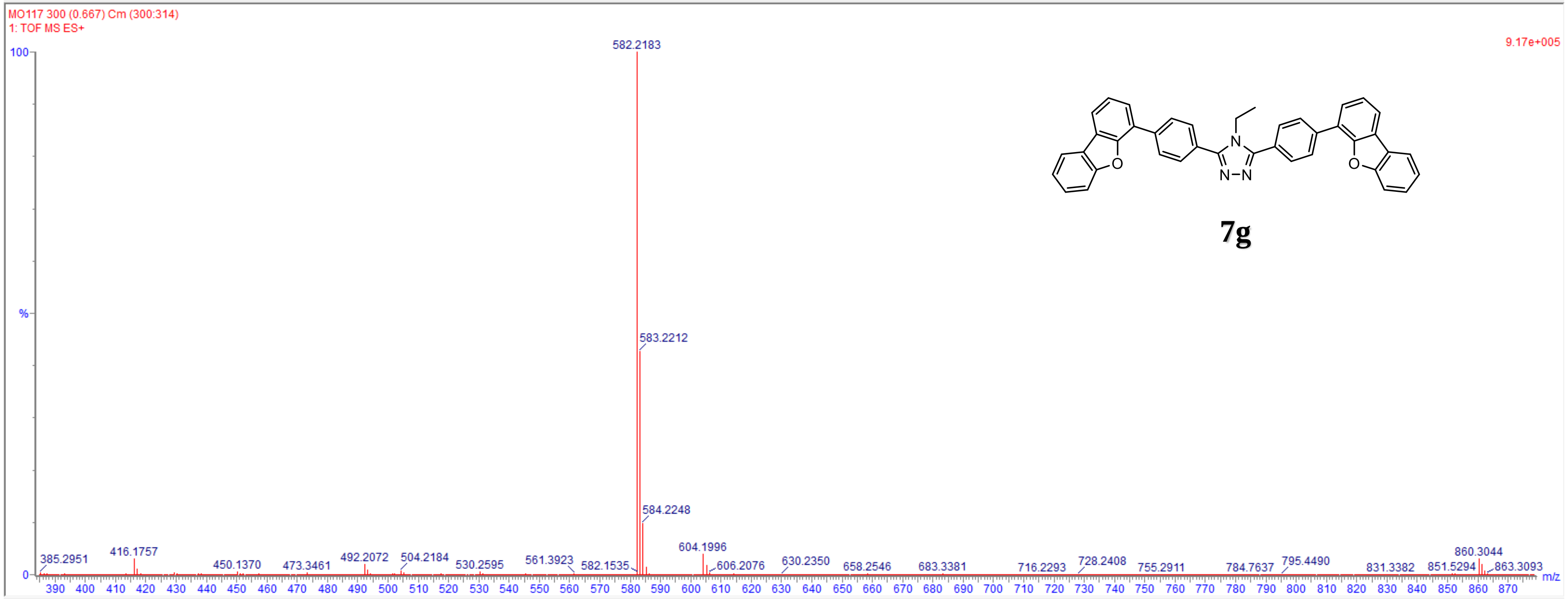

4-Ethyl-3,5-bis[4-(9-methyl-9H-carbazol-3-yl)phenyl]-4H-1,2,4-triazole (**7h**).

Tolerance = 100.0 mDa / DBE: min = -10.0, max = 50.0
Element prediction: Off
Number of isotope peaks used for i-FIT = 2
Monoisotopic Mass, Even Electron Ions
93 formula(e) evaluated with 19 results within limits (up to 5 closest results for each mass)
Elements Used:

Mass	RA	Calc. Mass	mDa	PPM	DBE	Formula	i-FIT	i-FIT Norm	Fit Conf %	C	H	N	O
608.2817	100.00	608.2814	0.3	0.5	28.5	C42 H34 N5	236.4	0.344	70.91	42	34	5	
		608.2702	11.5	18.9	28.5	C43 H34 N3 O	237.7	1.633	19.54	43	34	3	1
		608.2953	-13.6	-22.4	27.5	C45 H38 N O	240.3	4.200	1.50	45	38	1	1
		608.3026	-20.9	-34.4	23.5	C39 H38 N5 O2	240.5	4.382	1.25	39	38	5	2
		608.2590	22.7	37.3	28.5	C44 H34 N O2	238.8	2.687	6.81	44	34	1	2

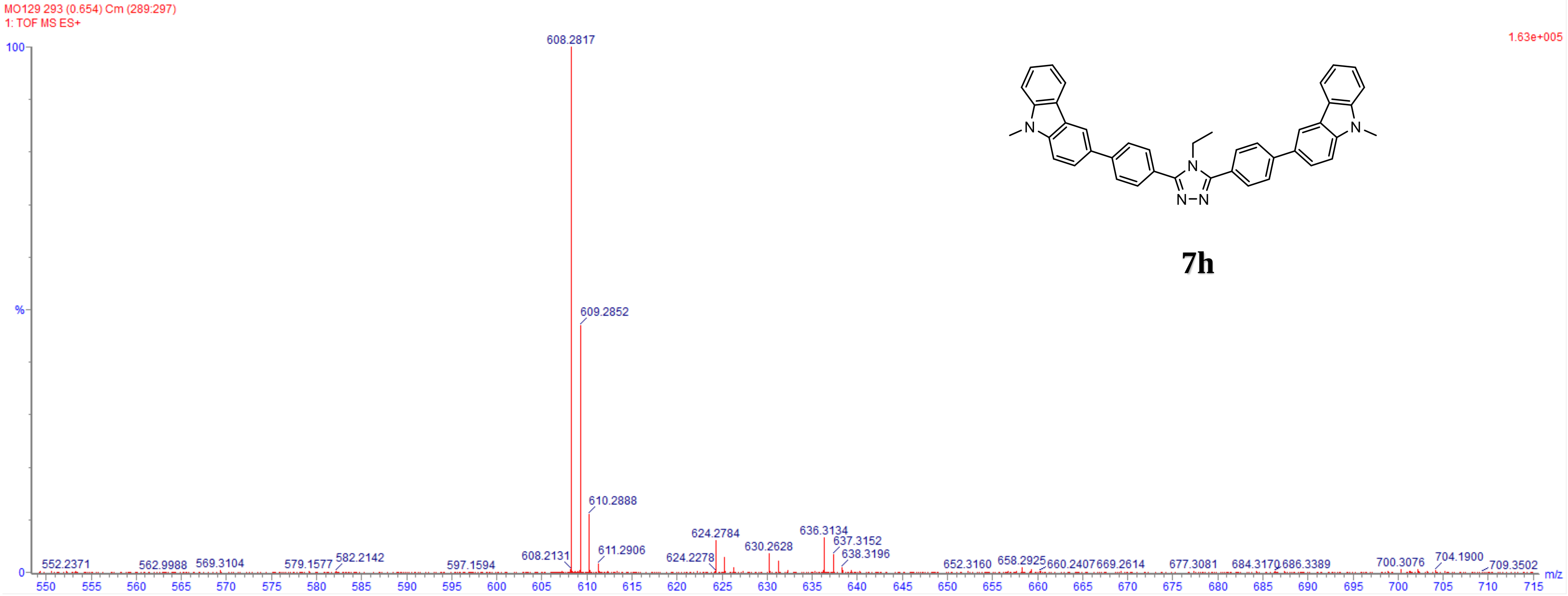

4-Ethyl-3,5-bis[4-(9-ethyl-9H-carbazol-3-yl)phenyl]-4H-1,2,4-triazole (**7i**).

Tolerance = 100.0 mDa / DBE: min = -10.0, max = 50.0
Element prediction: Off
Number of isotope peaks used for i-FIT = 2
Monoisotopic Mass, Even Electron Ions
93 formula(e) evaluated with 19 results within limits (up to 5 closest results for each mass)
Elements Used:

Mass	RA	Calc. Mass	mDa	PPM	DBE	Formula	i-FIT	i-FIT Norm	Fit Conf %	C	H	N	O
636.3124	100.00	636.3127	-0.3	-0.5	28.5	C44 H38 N5	216.1	5.592	0.37	44	38	5	
		636.3015	10.9	17.1	28.5	C45 H38 N3 O	216.5	6.050	0.24	45	38	3	1
		636.3266	-14.2	-22.3	27.5	C47 H42 N O	217.5	7.043	0.09	47	42	1	1
		636.3339	-21.5	-33.8	23.5	C41 H42 N5 O2	210.5	0.008	99.16	41	42	5	2
		636.2903	22.1	34.7	28.5	C46 H38 N O2	217.0	6.555	0.14	46	38	1	2

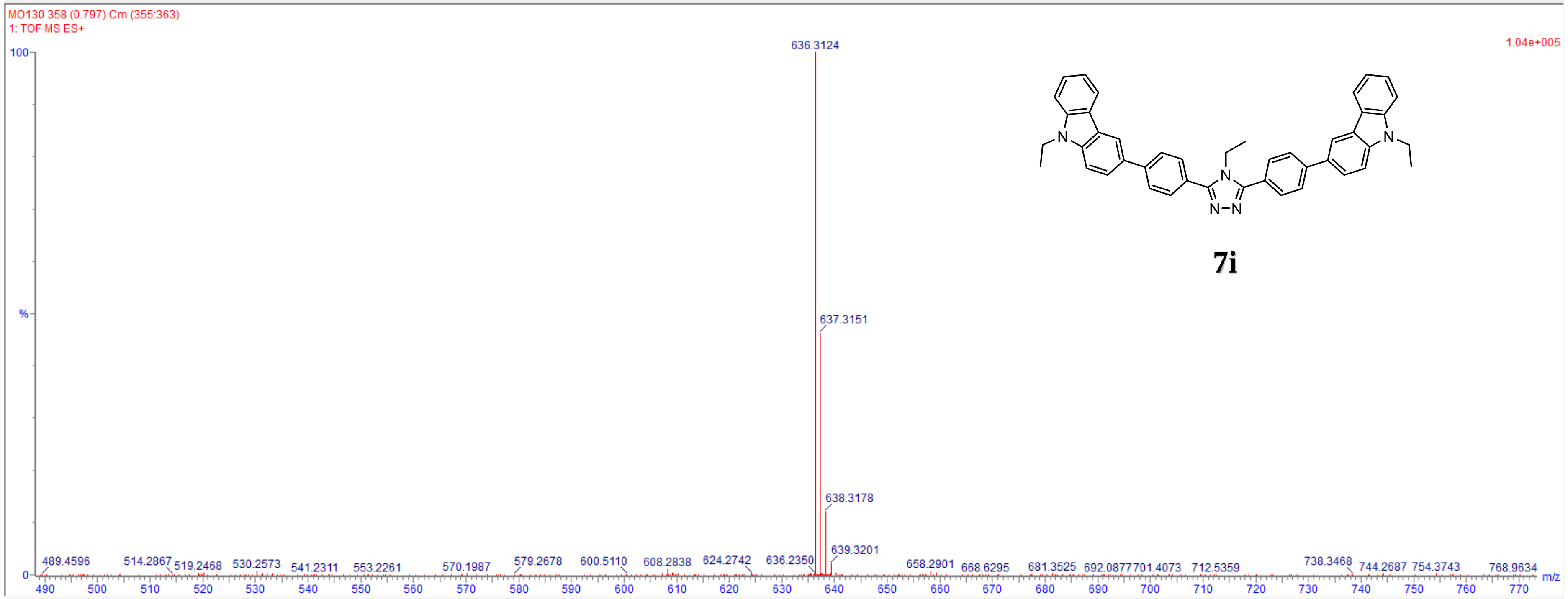

3,5-Bis[4′-(9H-carbazol-9-yl)biphenyl-4-yl]-4-ethyl-4H-1,2,4-triazole (**7j**).

Multiple Mass Analysis: 2 mass(es) processed
Tolerance = 100.0 mDa / DBE: min = -10.0, max = 50.0
Element prediction: Off
Number of isotope peaks used for i-FIT = 2
Monoisotopic Mass, Even Electron Ions
186 formula(e) evaluated with 38 results within limits (up to 5 closest results for each mass)
Elements Used:

Mass	RA	Calc. Mass	mDa	PPM	DBE	Formula	i-FIT	i-FIT Norm	Fit Conf %	C	H	N	O
732.3126	100.00	732.3127	-0.1	-0.1	36.5	C52 H38 N5	386.0	2.607	7.38	52	38	5	
		732.3015	11.1	15.2	36.5	C53 H38 N3 O	386.6	3.198	4.09	53	38	3	1
		732.3266	-14.0	-19.1	35.5	C55 H42 N O	388.0	4.579	1.03	55	42	1	1
		732.3339	-21.3	-29.1	31.5	C49 H42 N5 O2	383.6	0.158	85.34	49	42	5	2
		732.2903	22.3	30.5	36.5	C54 H38 N O2	387.3	3.832	2.17	54	38	1	2
733.3152	56.29	733.3107	4.5	6.1	35.5	C55 H41 O2	379.5	1.541	21.41	55	41		2
		733.3219	-6.7	-9.1	35.5	C54 H41 N2 O	379.5	1.495	22.43	54	41	2	1
		733.3331	-17.9	-24.4	35.5	C53 H41 N4	379.6	1.572	20.76	53	41	4	

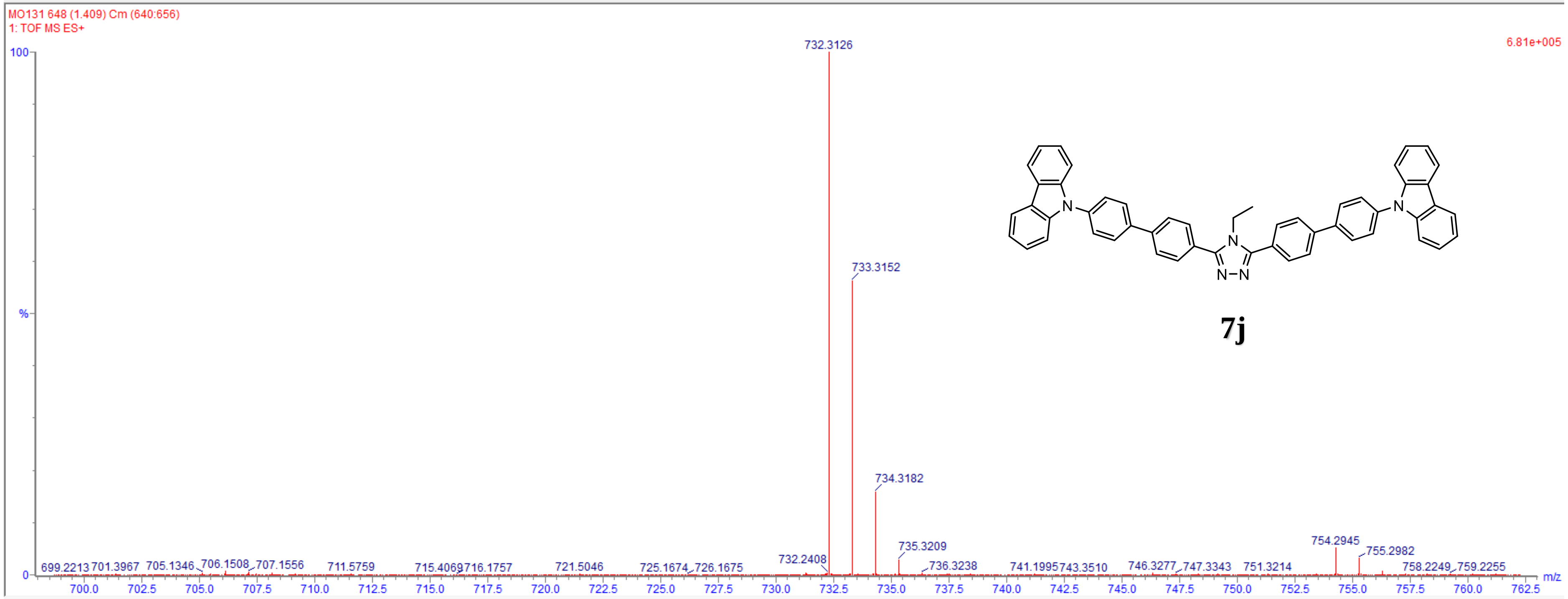

7j

4-Ethyl-3,5-bis[4-(thiantren-1-yl)phenyl]-4H-1,2,4-triazole (**7k**).

Tolerance = 100.0 mDa / DBE: min = -10.0, max = 50.0
Element prediction: Off
Number of isotope peaks used for i-FIT = 2
Monoisotopic Mass, Even Electron Ions
103 formula(e) evaluated with 21 results within limits (up to 5 closest results for each mass)
Elements Used:

Mass	RA	Calc. Mass	mDa	PPM	DBE	Formula	i-FIT	i-FIT Norm	Fit Conf %	C	H	N	S
678.1166	100.00	678.1166	0.0	0.0	28.5	C40 H28 N3 S4	537.1	0.300	74.08	40	28	3	4
		678.1132	3.4	5.0	33.5	C43 H24 N3 S3	538.7	1.829	16.05	43	24	3	3
		678.1099	6.7	9.9	38.5	C46 H20 N3 S2	539.7	2.827	5.92	46	20	3	2
		678.1065	10.1	14.9	43.5	C49 H16 N3 S	540.4	3.577	2.80	49	16	3	1
		678.1283	-11.7	-17.3	47.5	C54 H16 N	541.3	4.467	1.15	54	16	1	

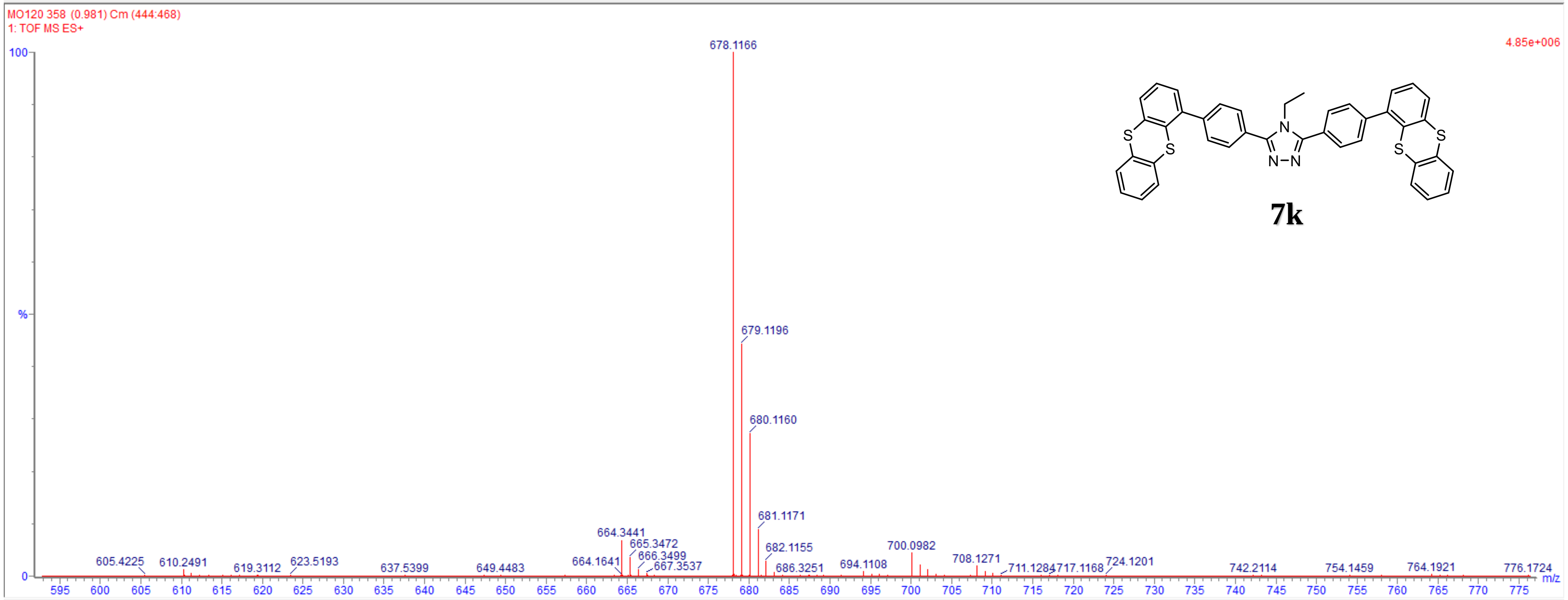

3,5-Bis[4′-(N,N-diphenylamino)biphenyl-4-yl]-4-propyl-4H-1,2,4-triazole (**8a**).

Multiple Mass Analysis: 2 mass(es) processed
Tolerance = 100.0 mDa / DBE: min = -10.0, max = 50.0
Element prediction: Off
Number of isotope peaks used for i-FIT = 2
Monoisotopic Mass, Even Electron Ions
62 formula(e) evaluated with 13 results within limits (up to 5 closest results for each mass)
Elements Used:

Mass	RA	Calc. Mass	mDa	PPM	DBE	Formula	i-FIT	i-FIT Norm	Fit Conf %	C	H	N
750.3588	100.00	750.3597	-0.9	-1.2	34.5	C53 H44 N5	207.1	0.635	53.02	53	44	5
		750.3848	-26.0	-34.7	33.5	C55 H48 N3	207.8	1.393	24.84	55	48	3
		750.3161	42.7	56.9	39.5	C58 H40 N	208.9	2.432	8.78	58	40	1
		750.4100	-51.2	-68.2	32.5	C57 H52 N	209.0	2.504	8.18	57	52	1
		750.2909	67.9	90.5	40.5	C56 H36 N3	209.4	2.960	5.18	56	36	3
751.3626	51.72	751.3801	-17.5	-23.3	33.5	C54 H47 N4	103.6	0.940	39.06	54	47	4
		751.3365	26.1	34.7	38.5	C59 H43	104.2	1.517	21.94	59	43	
		751.4052	-42.6	-56.7	32.5	C56 H51 N2	104.4	1.659	19.03	56	51	2

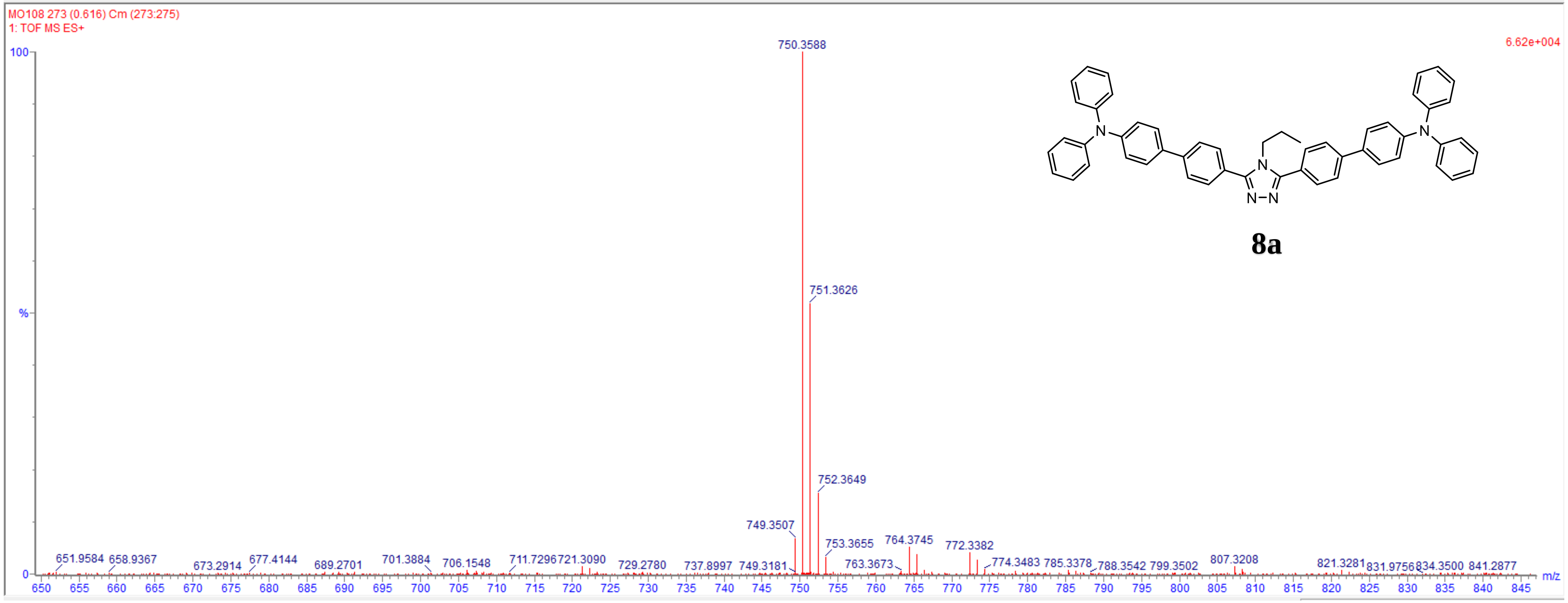

4-Butyl-3,5-bis[4′-(N,N-diphenylamino)biphenyl-4-yl]-4H-1,2,4-triazole (**9a**).

Multiple Mass Analysis: 2 mass(es) processed
Tolerance = 100.0 mDa / DBE: min = -10.0, max = 50.0
Element prediction: Off
Number of isotope peaks used for i-FIT = 2
Monoisotopic Mass, Even Electron Ions
62 formula(e) evaluated with 13 results within limits (up to 5 closest results for each mass)
Elements Used:

Mass	RA	Calc. Mass	mDa	PPM	DBE	Formula	i-FIT	i-FIT Norm	Fit Conf %	C	H	N
764.3749	100.00	764.3753	-0.4	-0.5	34.5	C54 H46 N5	185.1	0.325	72.23	54	46	5
		764.4005	-25.6	-33.5	33.5	C56 H50 N3	186.6	1.739	17.57	56	50	3
		764.3317	43.2	56.5	39.5	C59 H42 N	188.2	3.361	3.47	59	42	1
		764.4256	-50.7	-66.3	32.5	C58 H54 N	188.1	3.240	3.92	58	54	1
		764.3066	68.3	89.4	40.5	C57 H38 N3	188.4	3.570	2.81	57	38	3
765.3790	57.76	765.3957	-16.7	-21.8	33.5	C55 H49 N4	120.4	1.236	29.04	55	49	4
		765.3521	26.9	35.1	38.5	C60 H45	120.9	1.742	17.52	60	45	
		765.4209	-41.9	-54.7	32.5	C57 H53 N2	120.8	1.554	21.15	57	53	2

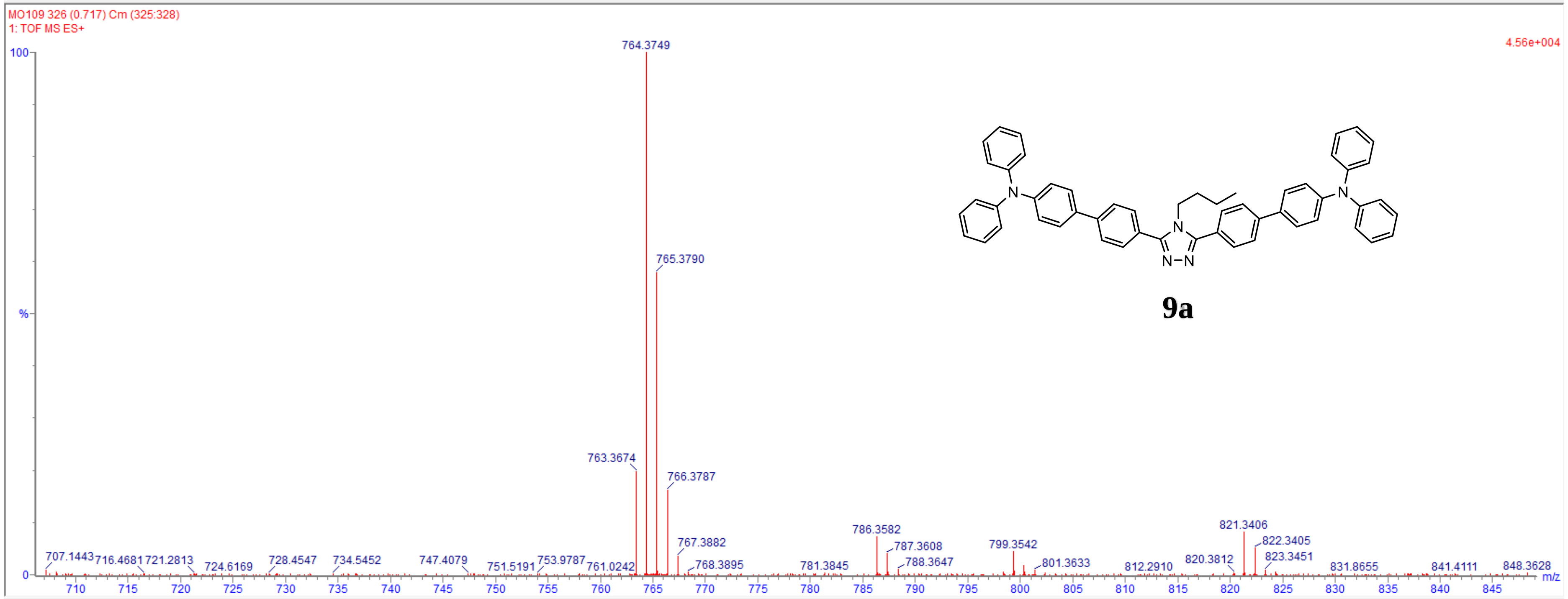

9a

4-Butyl-3,5-bis[4-(naphthalen-1-yl)phenyl]-4H-1,2,4-triazole (**9b**).

Tolerance = 100.0 mDa / DBE: min = -10.0, max = 50.0
Element prediction: Off
Number of isotope peaks used for i-FIT = 2
Monoisotopic Mass, Even Electron Ions
93 formula(e) evaluated with 20 results within limits (up to 5 closest results for each mass)
Elements Used:

Mass	RA	Calc. Mass	mDa	PPM	DBE	Formula	i-FIT	i-FIT Norm	Fit Conf %	C	H	N	O
530.2596	100.00	530.2596	0.0	0.0	24.5	C38 H32 N3	257.7	0.187	82.98	38	32	3	
		530.2556	4.0	7.5	20.5	C33 H32 N5 O2	264.8	7.214	0.07	33	32	5	2
		530.2484	11.2	21.1	24.5	C39 H32 N O	259.6	2.058	12.77	39	32	1	1
		530.2808	-21.2	-40.0	19.5	C35 H36 N3 O2	263.5	5.983	0.25	35	36	3	2
		530.2345	25.1	47.3	25.5	C36 H28 N5	260.8	3.240	3.92	36	28	5	

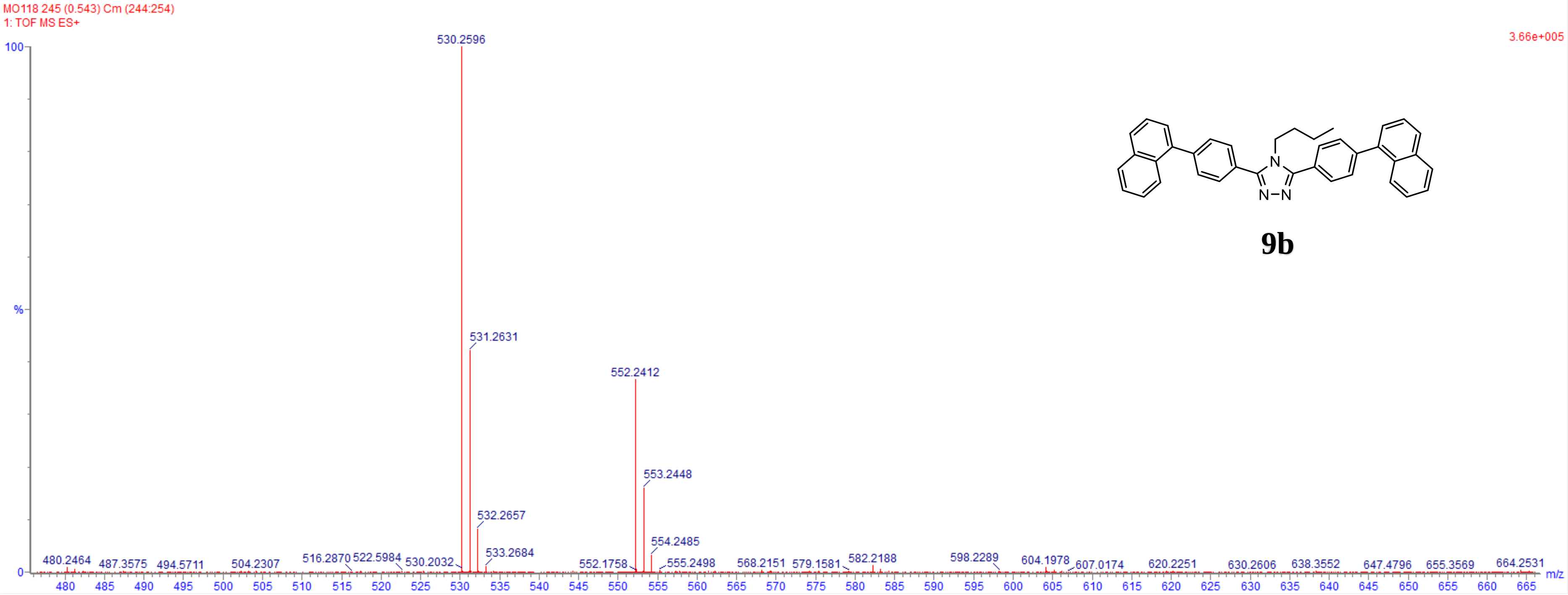

9b

4-Butyl-3,5-bis[4-(naphthalen-2-yl)phenyl]-4H-1,2,4-triazole (**9c**).

Tolerance = 100.0 mDa / DBE: min = -10.0, max = 50.0
Element prediction: Off
Number of isotope peaks used for i-FIT = 2
Monoisotopic Mass, Even Electron Ions
93 formula(e) evaluated with 20 results within limits (up to 5 closest results for each mass)
Elements Used:

Mass	RA	Calc. Mass	mDa	PPM	DBE	Formula	i-FIT	i-FIT Norm	Fit Conf %	C	H	N	O
530.2598	100.00	530.2596	0.2	0.4	24.5	C38 H32 N3	387.0	5.431	0.44	38	32	3	
		530.2556	4.2	7.9	20.5	C33 H32 N5 O2	382.6	1.017	36.15	33	32	5	2
		530.2484	11.4	21.5	24.5	C39 H32 N O	387.4	5.761	0.31	39	32	1	1
		530.2808	-21.0	-39.6	19.5	C35 H36 N3 O2	382.1	0.463	62.97	35	36	3	2
		530.2848	-25.0	-47.1	23.5	C40 H36 N	388.3	6.663	0.13	40	36	1	

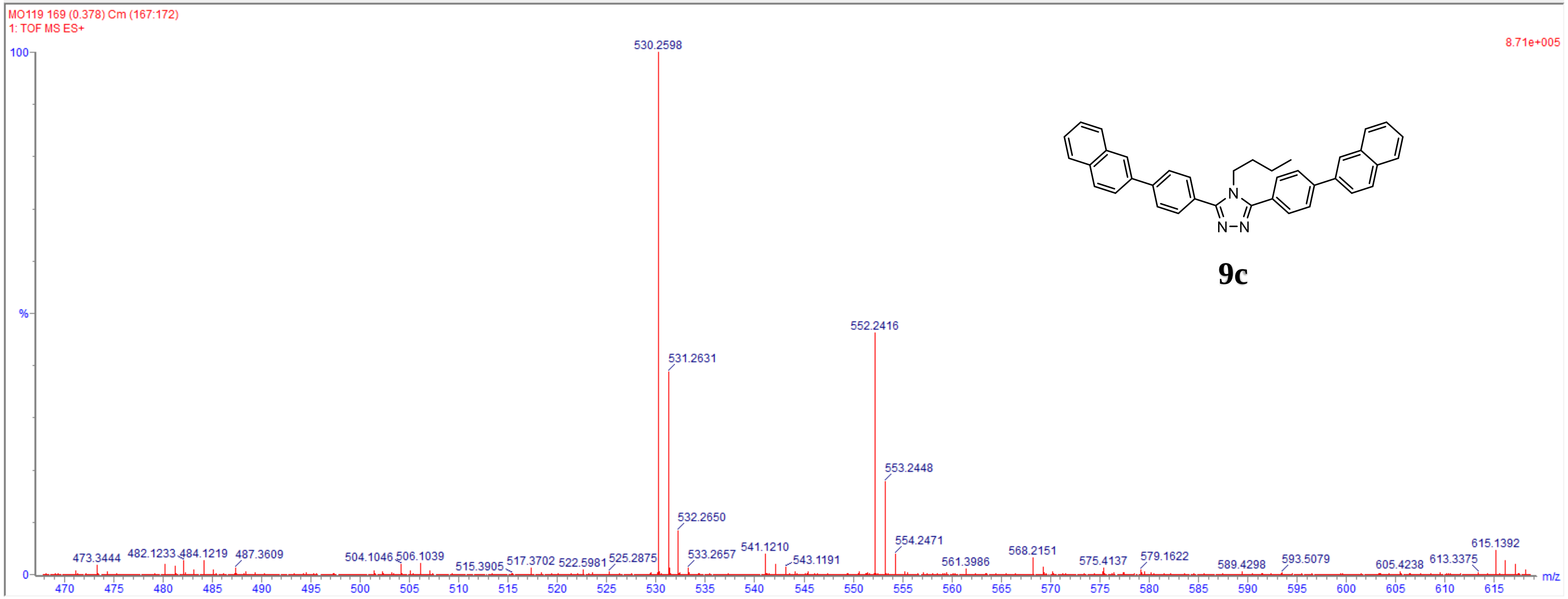

4-Butyl-3,5-bis[4-(quinolin-3-yl)phenyl]-4H-1,2,4-triazole (**9d**).

Tolerance = 100.0 mDa / DBE: min = -10.0, max = 50.0
Element prediction: Off
Number of isotope peaks used for i-FIT = 2
Monoisotopic Mass, Even Electron Ions
93 formula(e) evaluated with 19 results within limits (up to 5 closest results for each mass)
Elements Used:

Mass	RA	Calc. Mass	mDa	PPM	DBE	Formula	i-FIT	i-FIT Norm	Fit Conf %	C	H	N	O
532.2507	100.00	532.2501	0.6	1.1	24.5	C36 H30 N5	400.3	6.853	0.11	36	30	5	
		532.2389	11.8	22.2	24.5	C37 H30 N3 O	400.8	7.418	0.06	37	30	3	1
		532.2640	-13.3	-25.0	23.5	C39 H34 N O	402.0	8.620	0.02	39	34	1	1
		532.2713	-20.6	-38.7	19.5	C33 H34 N5 O2	393.4	0.002	99.78	33	34	5	2
		532.2277	23.0	43.2	24.5	C38 H30 N O2	401.4	8.008	0.03	38	30	1	2

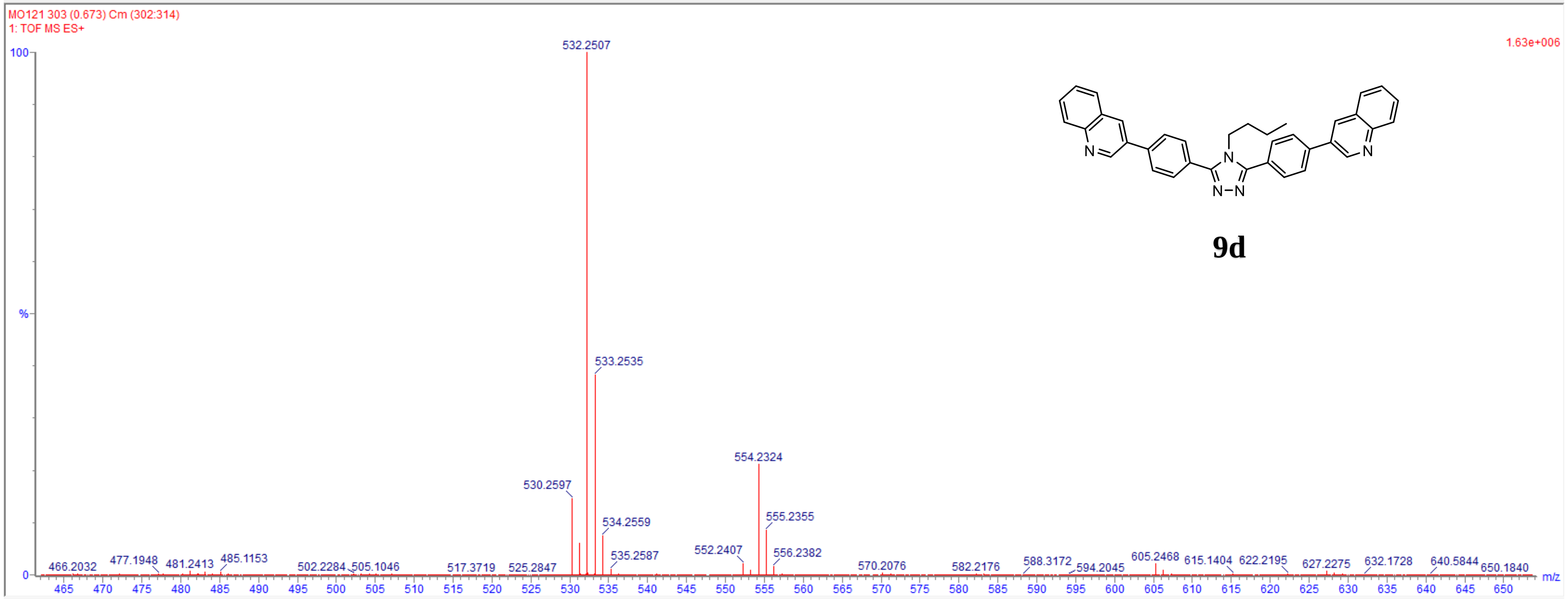

4-Butyl-3,5-bis[4-(quinolin-6-yl)phenyl]-4H-1,2,4-triazole (**9e**).

Tolerance = 100.0 mDa / DBE: min = -10.0, max = 50.0
Element prediction: Off
Number of isotope peaks used for i-FIT = 2
Monoisotopic Mass, Even Electron Ions
93 formula(e) evaluated with 19 results within limits (up to 5 closest results for each mass)
Elements Used:

Mass	RA	Calc. Mass	mDa	PPM	DBE	Formula	i-FIT	i-FIT Norm	Fit Conf %	C	H	N	O
532.2504	100.00	532.2501	0.3	0.6	24.5	C36 H30 N5	201.3	2.477	8.40	36	30	5	
		532.2389	11.5	21.6	24.5	C37 H30 N3 O	201.6	2.781	6.20	37	30	3	1
		532.2640	-13.6	-25.6	23.5	C39 H34 N O	202.3	3.488	3.06	39	34	1	1
		532.2713	-20.9	-39.3	19.5	C33 H34 N5 O2	199.1	0.246	78.21	33	34	5	2
		532.2277	22.7	42.6	24.5	C38 H30 N O2	202.0	3.186	4.13	38	30	1	2

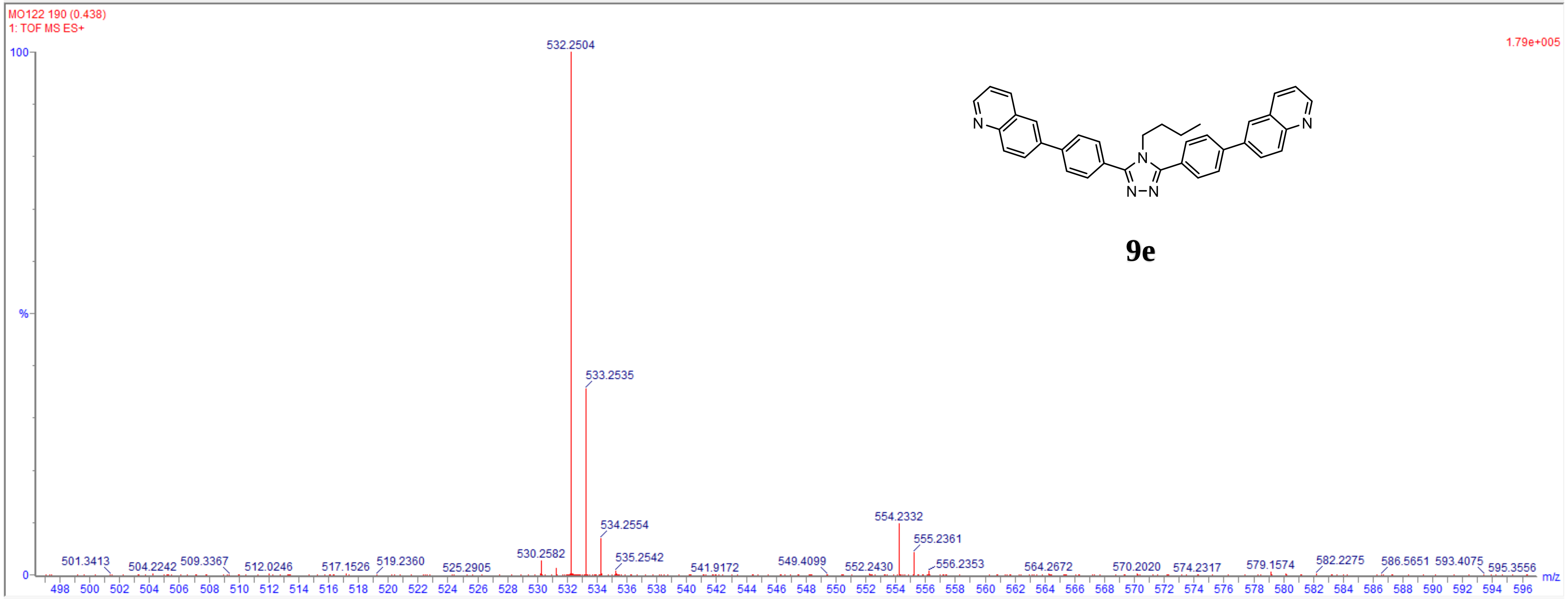

3,5-Bis[4-(dibenzothiophen-4-yl)phenyl]-4-butyl-4H-1,2,4-triazole (**9f**).

Tolerance = 100.0 mDa / DBE: min = -10.0, max = 50.0
Element prediction: Off
Number of isotope peaks used for i-FIT = 2
Monoisotopic Mass, Even Electron Ions
103 formula(e) evaluated with 23 results within limits (up to 5 closest results for each mass)
Elements Used:

Mass	RA	Calc. Mass	mDa	PPM	DBE	Formula	i-FIT	i-FIT Norm	Fit Conf %	C	H	N	S
642.2034	100.00	642.2038	-0.4	-0.6	28.5	C42 H32 N3 S2	153.6	0.040	96.10	42	32	3	2
		642.2004	3.0	4.7	33.5	C45 H28 N3 S	158.1	4.623	0.98	45	28	3	1
		642.2071	-3.7	-5.8	23.5	C39 H36 N3 S3	157.2	3.678	2.53	39	36	3	3
		642.1970	6.4	10.0	38.5	C48 H24 N3	160.1	6.571	0.14	48	24	3	
		642.2105	-7.1	-11.1	18.5	C36 H40 N3 S4	159.5	5.977	0.25	36	40	3	4

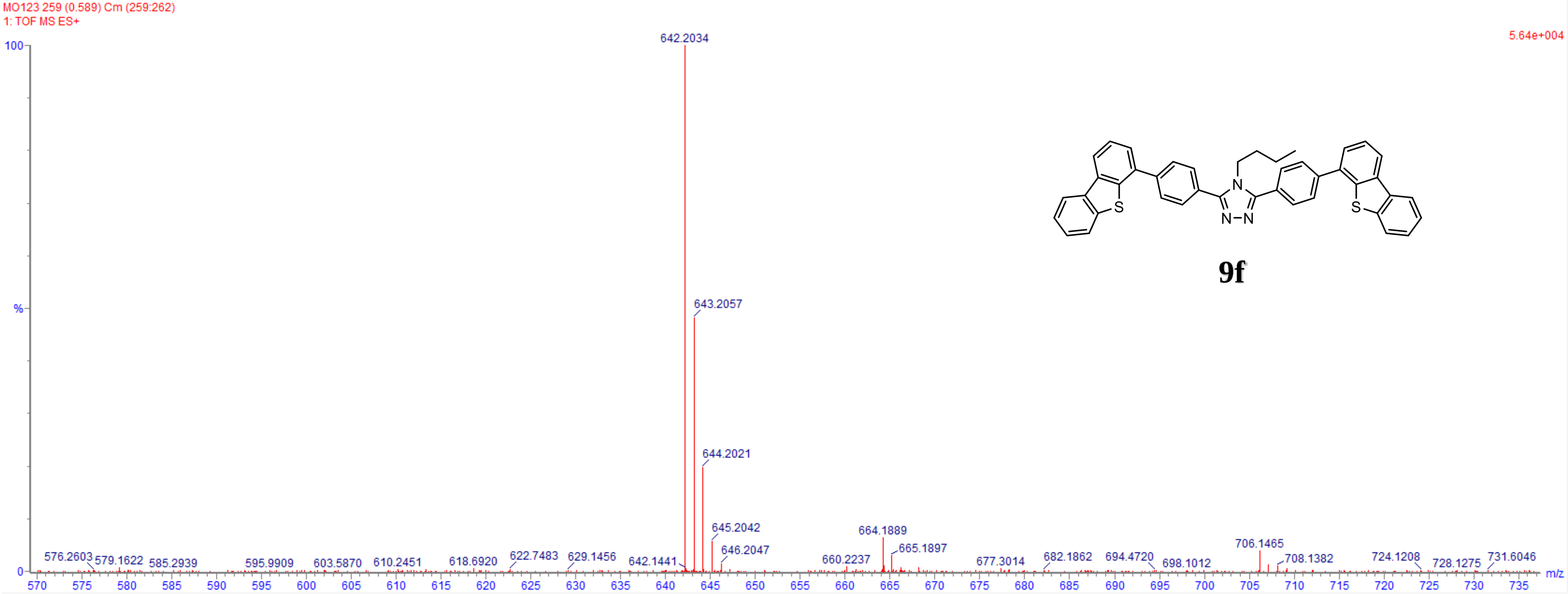

9f

3,5-Bis[4-(dibenzofuran-4-yl)phenyl]-4-butyl-4H-1,2,4-triazole (**9g**).

Tolerance = 100.0 mDa / DBE: min = -10.0, max = 50.0
Element prediction: Off
Number of isotope peaks used for i-FIT = 2
Monoisotopic Mass, Even Electron Ions
62 formula(e) evaluated with 14 results within limits (up to 5 closest results for each mass)
Elements Used:

Mass	RA	Calc. Mass	mDa	PPM	DBE	Formula	i-FIT	i-FIT Norm	Fit Conf %	C	H	N	O
610.2491	100.00	610.2495	-0.4	-0.7	28.5	C42 H32 N3 O2	309.4	0.171	84.29	42	32	3	2
		610.2535	-4.4	-7.2	32.5	C47 H32 N	313.4	4.119	1.63	47	32	1	
		610.2283	20.8	34.1	33.5	C45 H28 N3	312.8	3.482	3.07	45	28	3	
		610.2746	-25.5	-41.8	27.5	C44 H36 N O2	311.7	2.386	9.20	44	36	1	2
		610.2171	32.0	52.4	33.5	C46 H28 N O	313.3	4.010	1.81	46	28	1	1

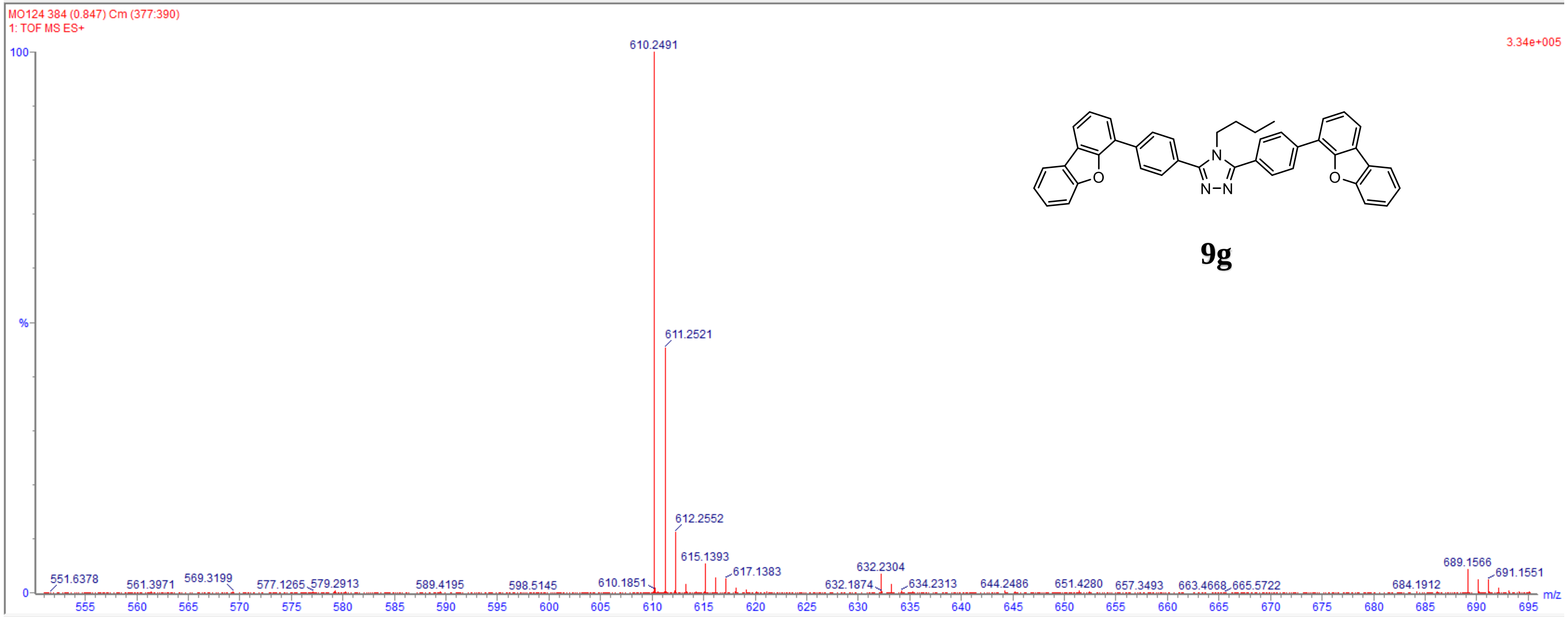

9g

4-Butyl-3,5-bis[4-(9-methyl-9H-carbazol-3-yl)phenyl]-4H-1,2,4-triazole (**9h**).

Tolerance = 100.0 mDa / DBE: min = -10.0, max = 50.0
Element prediction: Off
Number of isotope peaks used for i-FIT = 2
Monoisotopic Mass, Even Electron Ions
93 formula(e) evaluated with 19 results within limits (up to 5 closest results for each mass)
Elements Used:

Mass	RA	Calc. Mass	mDa	PPM	DBE	Formula	i-FIT	i-FIT Norm	Fit Conf %	C	H	N	O
636.3123	100.00	636.3127	-0.4	-0.6	28.5	C44 H38 N5	111.3	2.953	5.22	44	38	5	
		636.3015	10.8	17.0	28.5	C45 H38 N3 O	111.9	3.566	2.83	45	38	3	1
		636.3266	-14.3	-22.5	27.5	C47 H42 N O	113.2	4.837	0.79	47	42	1	1
		636.3339	-21.6	-33.9	23.5	C41 H42 N5 O2	108.4	0.110	89.62	41	42	5	2
		636.2903	22.0	34.6	28.5	C46 H38 N O2	112.5	4.174	1.54	46	38	1	2

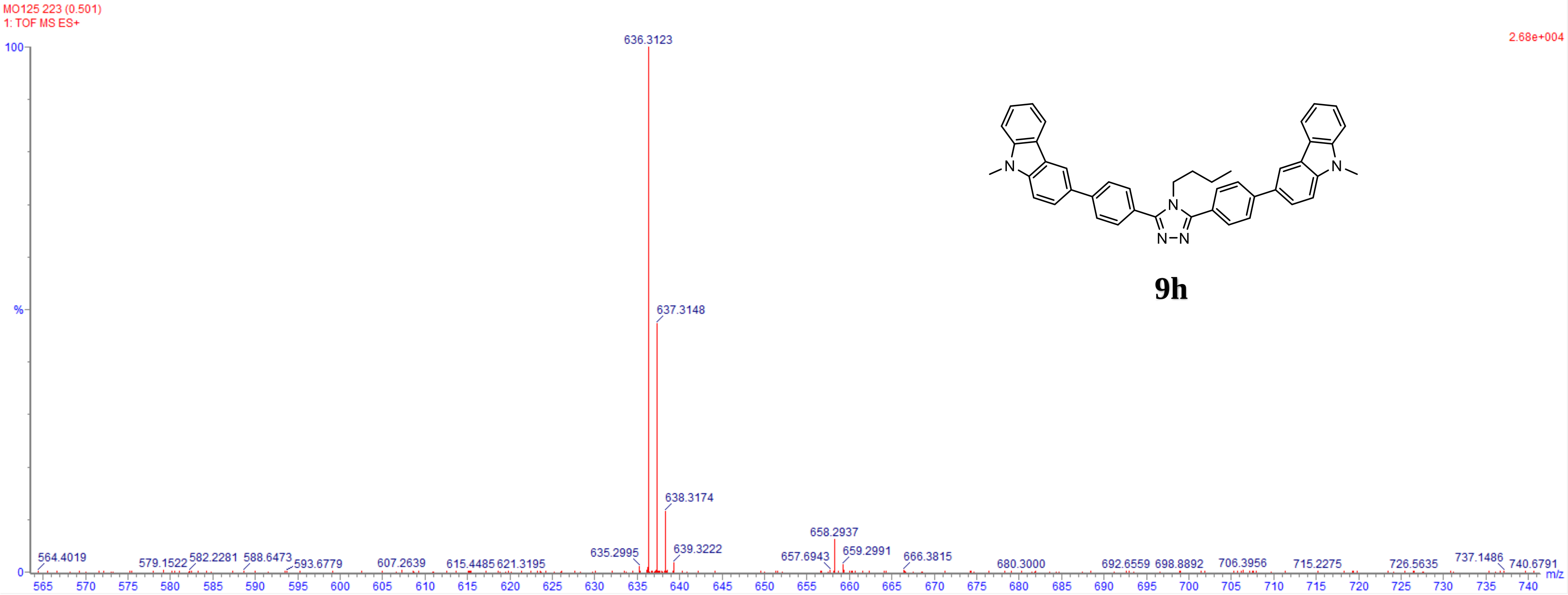

9h

4-Butyl-3,5-bis[4-(9-ethyl-9H-carbazol-3-yl)phenyl]-4H-1,2,4-triazole (**9i**).

Tolerance = 100.0 mDa / DBE: min = -10.0, max = 50.0
Element prediction: Off
Number of isotope peaks used for i-FIT = 2
Monoisotopic Mass, Even Electron Ions
31 formula(e) evaluated with 7 results within limits (up to 5 closest results for each mass)
Elements Used:

Mass	RA	Calc. Mass	mDa	PPM	DBE	Formula	i-FIT	i-FIT Norm	Fit Conf %	C	H	N
664.3439	100.00	664.3440	-0.1	-0.2	28.5	C46 H42 N5	169.4	0.404	66.77	46	42	5
		664.3692	-25.3	-38.1	27.5	C48 H46 N3	170.5	1.601	20.18	48	46	3
		664.3004	43.5	65.5	33.5	C51 H38 N	172.2	3.297	3.70	51	38	1
		664.3943	-50.4	-75.9	26.5	C50 H50 N	171.9	2.907	5.46	50	50	1
		664.2753	68.6	103.3	34.5	C49 H34 N3	172.2	3.246	3.89	49	34	3

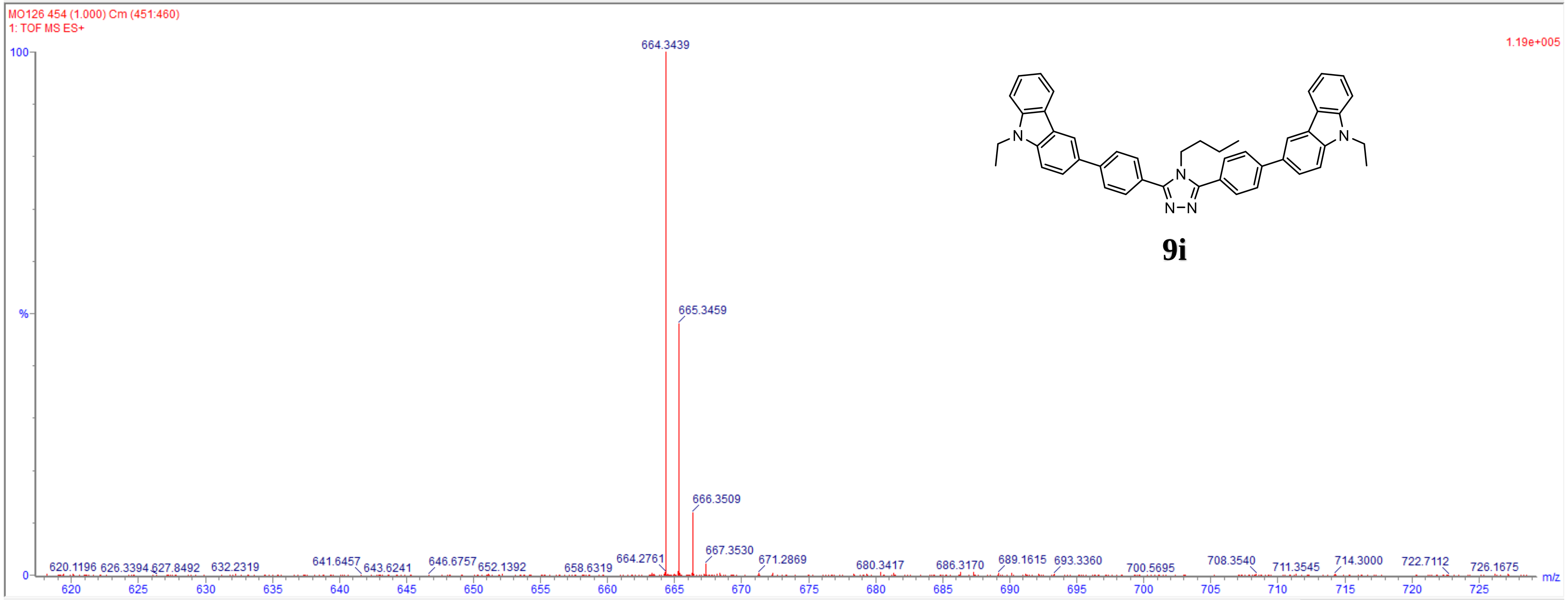

9i

4-Butyl-3,5-bis[4′-(9H-carbazol-9-yl)biphenyl-4-yl]-4H-1,2,4-triazole (**9j**).

Multiple Mass Analysis: 2 mass(es) processed
Tolerance = 100.0 mDa / DBE: min = -10.0, max = 50.0
Element prediction: Off
Number of isotope peaks used for i-FIT = 2
Monoisotopic Mass, Even Electron Ions
186 formula(e) evaluated with 38 results within limits (up to 5 closest results for each mass)
Elements Used:

Mass	RA	Calc. Mass	mDa	PPM	DBE	Formula	i-FIT	i-FIT Norm	Fit Conf %	C	H	N	O
760.3438	100.00	760.3440	-0.2	-0.3	36.5	C54 H42 N5	143.2	0.878	41.58	54	42	5	
		760.3328	11.0	14.5	36.5	C55 H42 N3 O	143.6	1.234	29.11	55	42	3	1
		760.3579	-14.1	-18.5	35.5	C57 H46 N O	145.2	2.853	5.77	57	46	1	1
		760.3652	-21.4	-28.1	31.5	C51 H46 N5 O2	145.2	2.863	5.71	51	46	5	2
		760.3216	22.2	29.2	36.5	C56 H42 N O2	144.0	1.724	17.83	56	42	1	2
761.3444	60.12	761.3420	2.4	3.2	35.5	C57 H45 O2	136.0	1.565	20.90	57	45		2
		761.3532	-8.8	-11.6	35.5	C56 H45 N2 O	135.9	1.519	21.88	56	45	2	1
		761.3280	16.4	21.5	36.5	C54 H41 N4 O	135.9	1.449	23.49	54	41	4	1

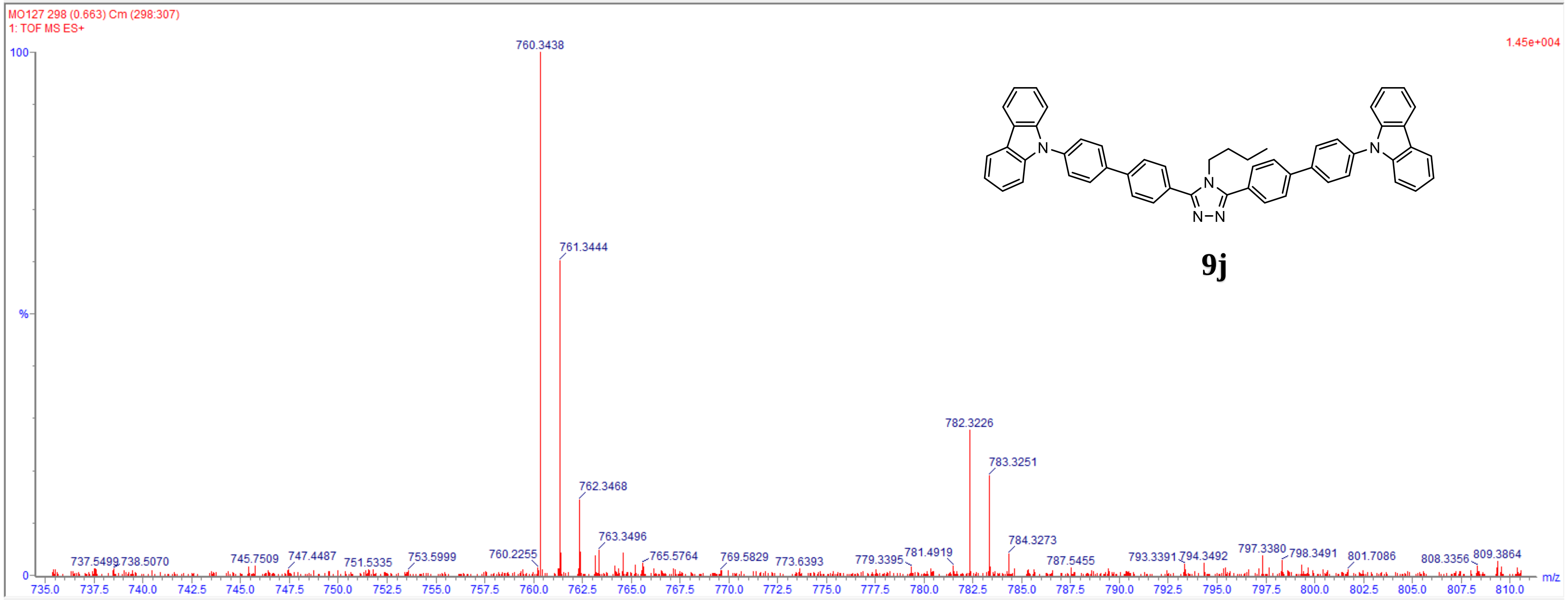

4-Butyl-3,5-bis[4-(thiantren-1-yl)phenyl]-4H-1,2,4-triazole (**9k**).

Tolerance = 100.0 mDa / DBE: min = -10.0, max = 50.0
Element prediction: Off
Number of isotope peaks used for i-FIT = 2
Monoisotopic Mass, Even Electron Ions
103 formula(e) evaluated with 21 results within limits (up to 5 closest results for each mass)
Elements Used:

Mass	RA	Calc. Mass	mDa	PPM	DBE	Formula	i-FIT	i-FIT Norm	Fit Conf %	C	H	N	S
706.1474	100.00	706.1479	-0.5	-0.7	28.5	C42 H32 N3 S4	327.8	0.249	77.99	42	32	3	4
		706.1445	2.9	4.1	33.5	C45 H28 N3 S3	329.5	1.954	14.17	45	28	3	3
		706.1412	6.2	8.8	38.5	C48 H24 N3 S2	330.6	3.028	4.84	48	24	3	2
		706.1378	9.6	13.6	43.5	C51 H20 N3 S	331.4	3.824	2.18	51	20	3	1
		706.1596	-12.2	-17.3	47.5	C56 H20 N	332.4	4.805	0.82	56	20	1	

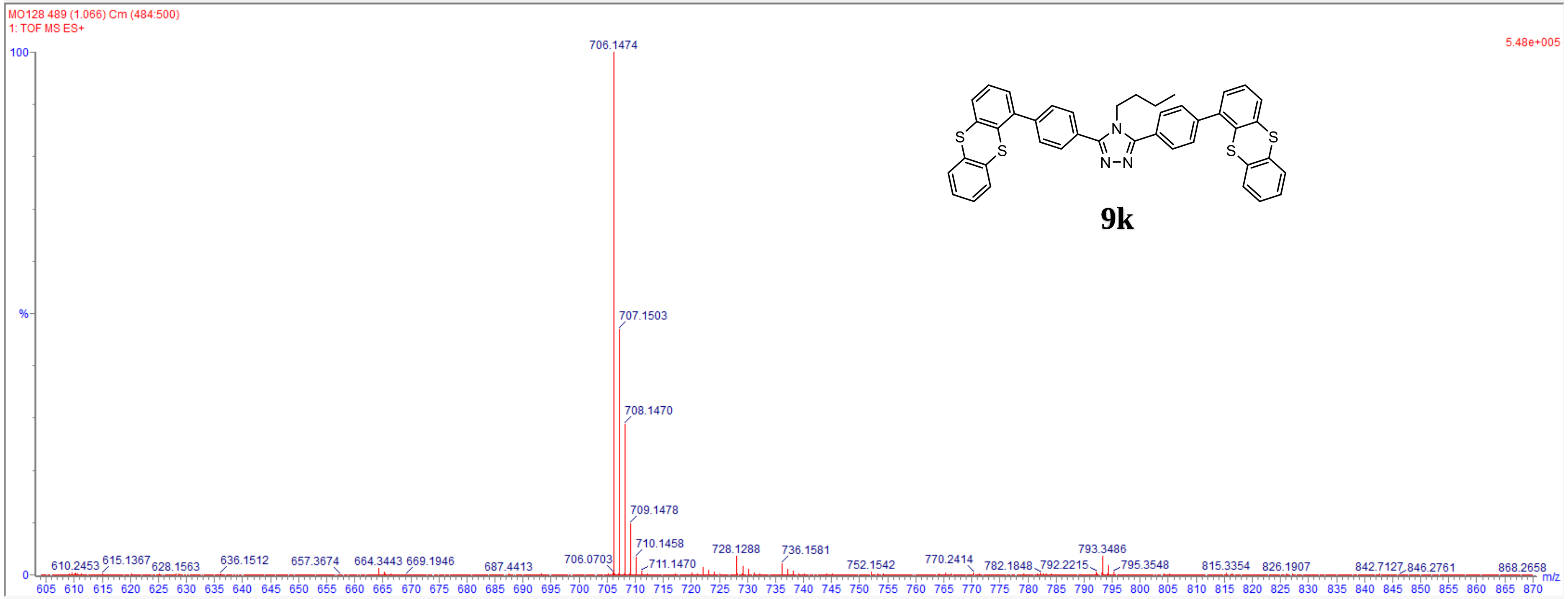

4-Hexyl-3,5-bis[4′-(N,N-diphenylamino)biphenyl-4-yl]-4H-1,2,4-triazole (**10a**).

Multiple Mass Analysis: 2 mass(es) processed
Tolerance = 100.0 mDa / DBE: min = -10.0, max = 50.0
Element prediction: Off
Number of isotope peaks used for i-FIT = 2
Monoisotopic Mass, Even Electron Ions
62 formula(e) evaluated with 11 results within limits (up to 5 closest results for each mass)
Elements Used:

Mass	RA	Calc. Mass	mDa	PPM	DBE	Formula	i-FIT	i-FIT Norm	Fit Conf %	C	H	N
792.4061	100.00	792.4066	-0.5	-0.6	34.5	C56 H50 N5	65.8	0.301	74.04	56	50	5
		792.4318	-25.7	-32.4	33.5	C58 H54 N3	67.3	1.835	15.96	58	54	3
		792.3630	43.1	54.4	39.5	C61 H46 N	69.1	3.570	2.82	61	46	1
		792.4569	-50.8	-64.1	32.5	C60 H58 N	68.7	3.252	3.87	60	58	1
		792.3379	68.2	86.1	40.5	C59 H42 N3	68.9	3.408	3.31	59	42	3
793.4078	60.02	793.4270	-19.2	-24.2	33.5	C57 H53 N4	53.2	0.996	36.92	57	53	4
		793.3834	24.4	30.8	38.5	C62 H49	53.5	1.320	26.71	62	49	
		793.4522	-44.4	-56.0	32.5	C59 H57 N2	54.0	1.768	17.07	59	57	2

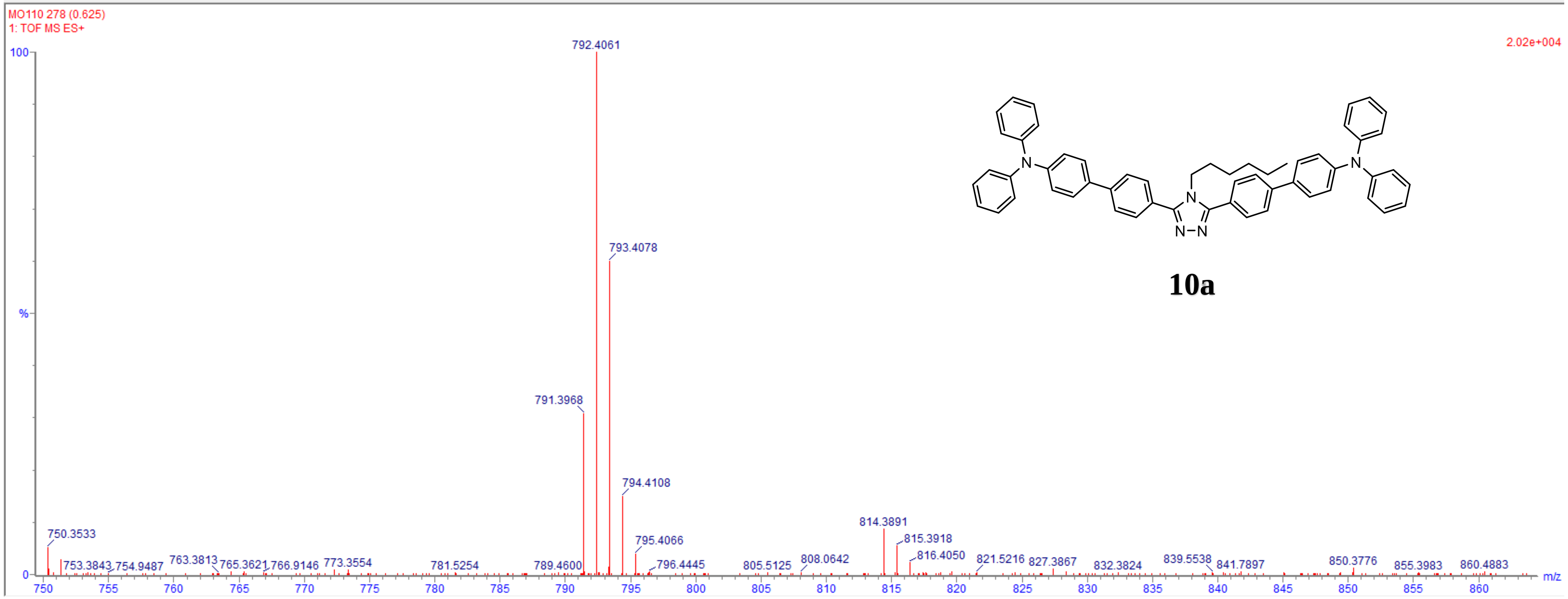

3. X-ray crystallography data

Crystal structure determination

X-ray intensity data of compounds **8a, 9a,** and **10a** were collected at 100.0(1) K, on a Rigaku Synergy Dualflex automatic diffractometer equipped with Pilatus 300K detector and micro-focus sealed PhotonJet X-ray tube. The mirror monochromated Cu*Kα* (λ = 1.54184 Å) radiation and shutterless ω scan mode was used. Lorentz, polarization and numerical absorption (based on gaussian integration over a multifaceted crystal model) corrections were applied during the data reduction. The structure was solved by dual-space algorithm. All non-hydrogen atoms were refined anisotropically using full-matrix, least-squares technique on F^2. All hydrogen atoms were found from difference Fourier synthesis after ten cycles of anisotropic refinement. Hydrogen atoms were refined as “riding” on the adjacent atom with geometric idealization after each cycle of refinement. Individual isotropic displacement factors of non-methyl and methyl H atoms were set to be equal 1.2 and 1.5 times the value of equivalent displacement factors of the parent atoms, respectively. The SHELXT,[1] SHELXL[2] and SHELXTL[3] programs were used for all calculations. Atomic scattering factors were taken from International Tables for Crystallography.[4] Details concerning crystal data and refinement are given in Table S1.

The solvent molecules of **8a** and **9a** are disordered over three and two positions of asymmetric unit, respectively. The presence of one solvent atom in special position *d* of $P2_1/c$ space group (with site symmetry -1 and multiplicity 2) causes that one void containing a disordered solvent occupies two asymmetric units. Some atoms of three disordered ethanol molecules of **8a** occupy the same position of crystal net, causing formal presence of six atom sites in the refinement model (three sites contain atoms belonging to two molecules; Figure S5). The occupancy of individual molecules is 0.40, 0.25, 0.10, what totally gives 0.75 solvent molecule per **8a** molecule. The ethanol molecule in **9a** is simply disordered over two positions (with 0.5 participation of each domain) and form 1:1 solvate. Due to disorder and partial occupancy the positions of hydrogen atoms of solvent molecules could not be reliably determined thus these hydrogen atoms were not included into refinements. To asses influence of solvents electron density on results of refinement, structures of **8a** and **9a** were refined against squeezed data.[5] The convergence and fit parameters of squeezed and non- squeezed data (taken as measured) are similar (Table S1) what proves that solvent refinement models (including sites occupancy) were reasonable. The slightly larger values of difference Fourier synthesis global local peaks (smaller than 0.4 e•$Å^{-3}$) originate mainly from absence of solvents hydrogen atoms in refinement. Beside the solvent molecules, the part of alkyl substituents of compounds **9a** and **10a** are also disordered over two positions. In case of compound **9a** two terminal atoms are disordered with 0.6:0.4 participation of domains, while in compound **10a** five terminal atoms are disordered with 0.5:0.5 participation over domains.

CCDC 1999468-1999472 contain the supplementary crystallographic data for compounds **8a**, **9a**, and **10a**. These data can be obtained free of charge *via* http://www.ccdc.cam.ac.uk/conts/retrieving.html, or from the Cambridge Crystallographic Data Centre, 12 Union Road, Cambridge CB2 1EZ, UK; fax: (+44) 1223-336-033; or e-mail: deposit@ccdc.cam.ac.uk

(1) G.M. Sheldrick, *Acta Crystallogr., Sect. A: Found. Adv.* 2015, **71**, 3-8
(2) G.M. Sheldrick, *Acta Crystallogr., Sect. C: Struct. Chem.* 2015, **71**, 3-8
(3) G.M. Sheldrick, *Acta Crystallogr., Sect. A: Found. Adv.* 2008, **64**, 112-122
(4) *International Tables for Crystallography, Volume C: Mathematical, Physical and Chemical Tables,* ed. E. Prince, Kluwer Academic Publishers, Dordrecht, 3rd edition, 2004
(5) A. L. Spek, *Acta Crystallogr., Sect. E: Crystallogr. Commun.*, 2020, **76**, 1-11

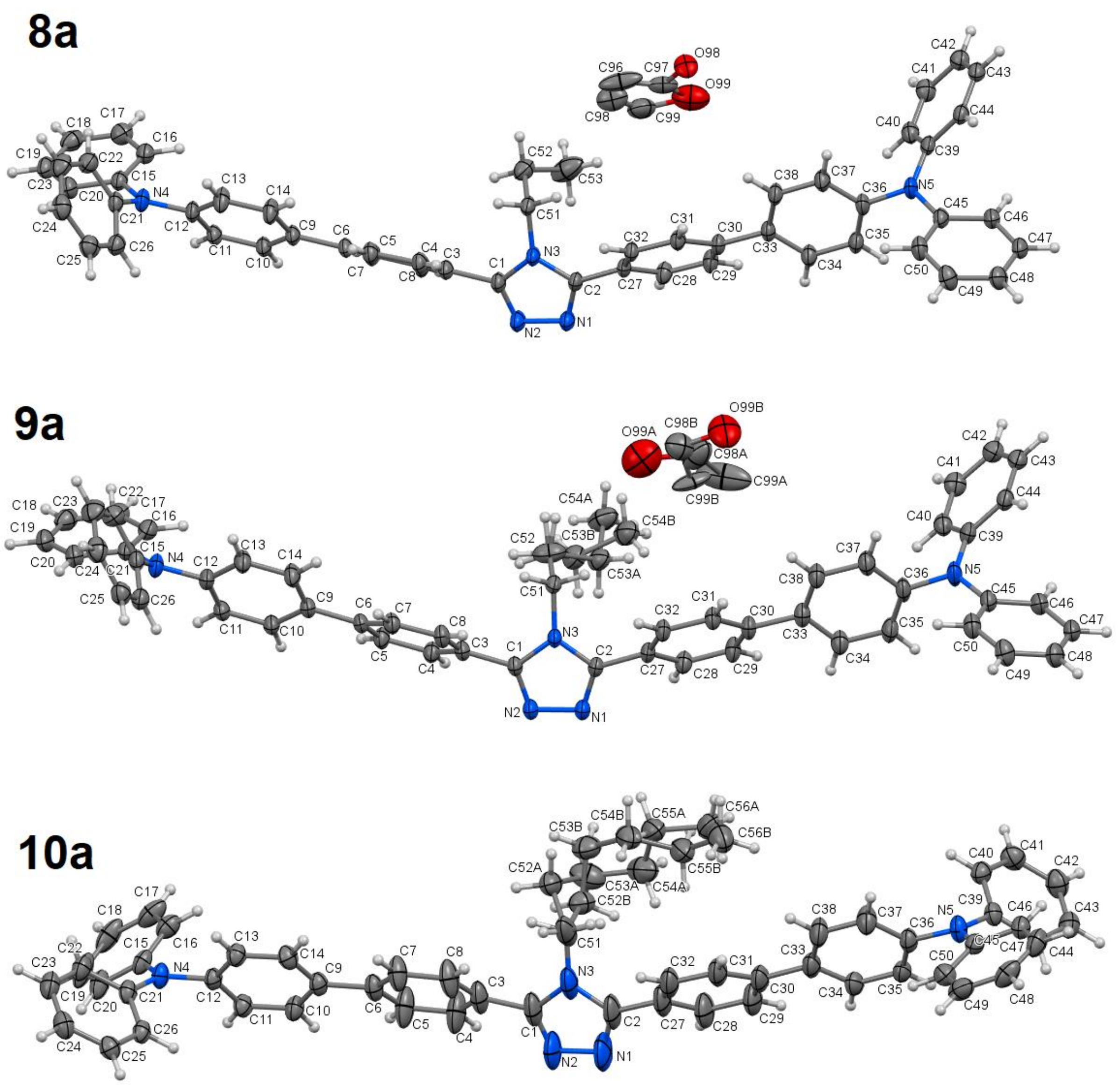

Figure S1. Complete asymmetric units of the structures of compounds **8a**, **9a**, and **10a**, with atom numbering scheme, plotted with 50% probability of displacement ellipsoids of non-hydrogen atoms. Hydrogen atoms are plotted as spheres of arbitrary radii.

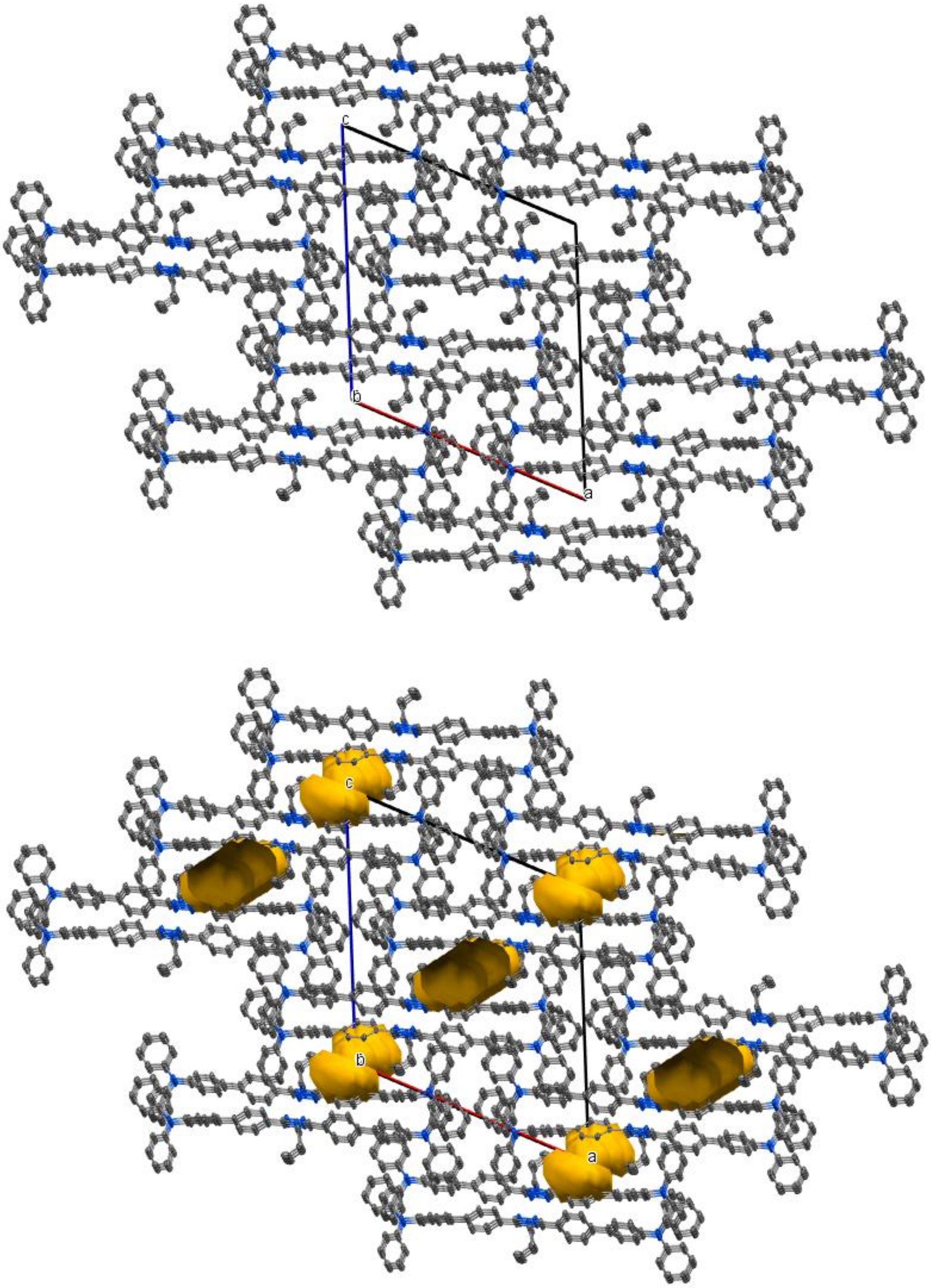

Figure S2. Solvent accessible voids within crystal structure of **8a**

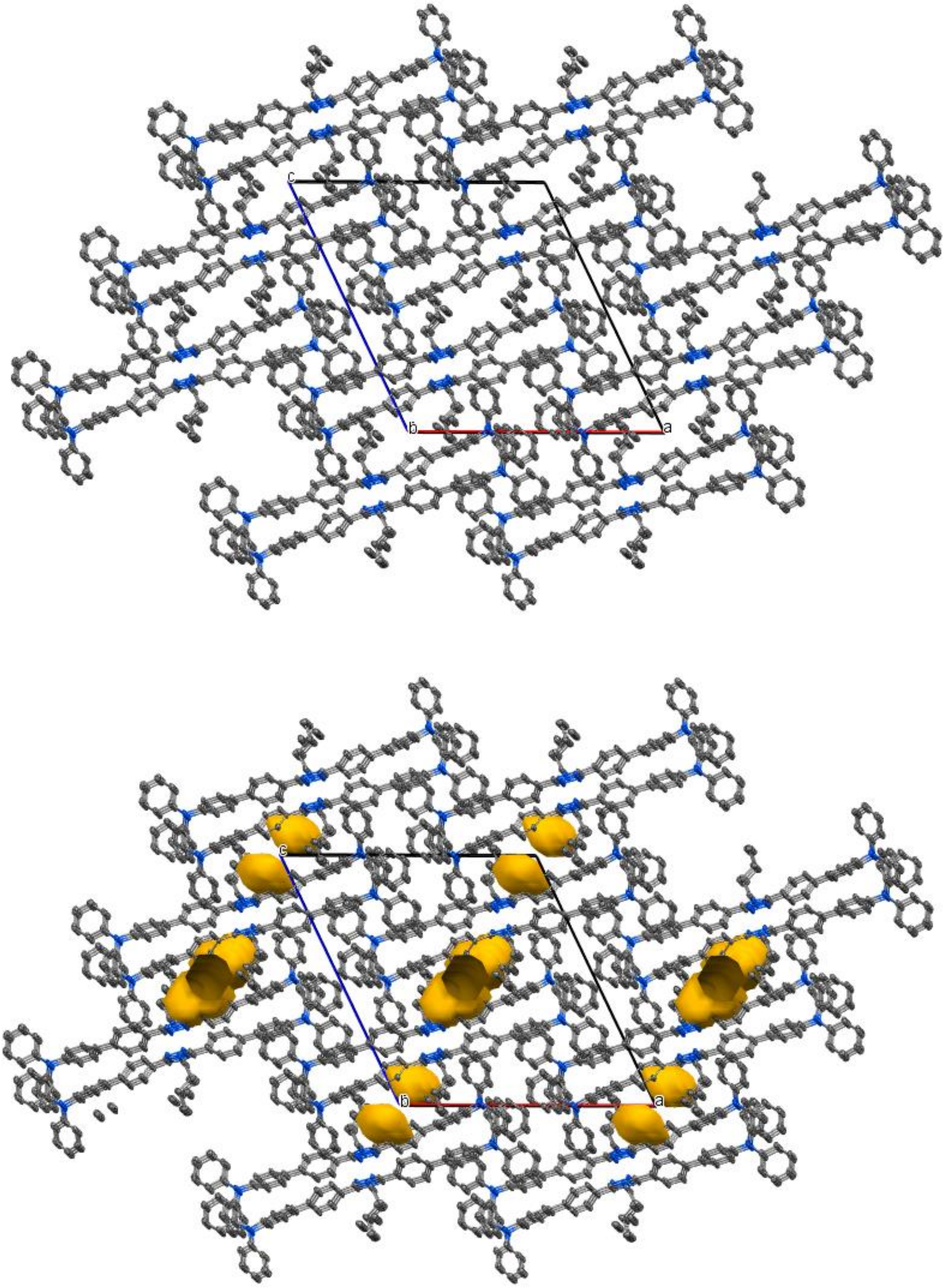

Figure S3. Solvent accessible voids within crystal structure of **9a**

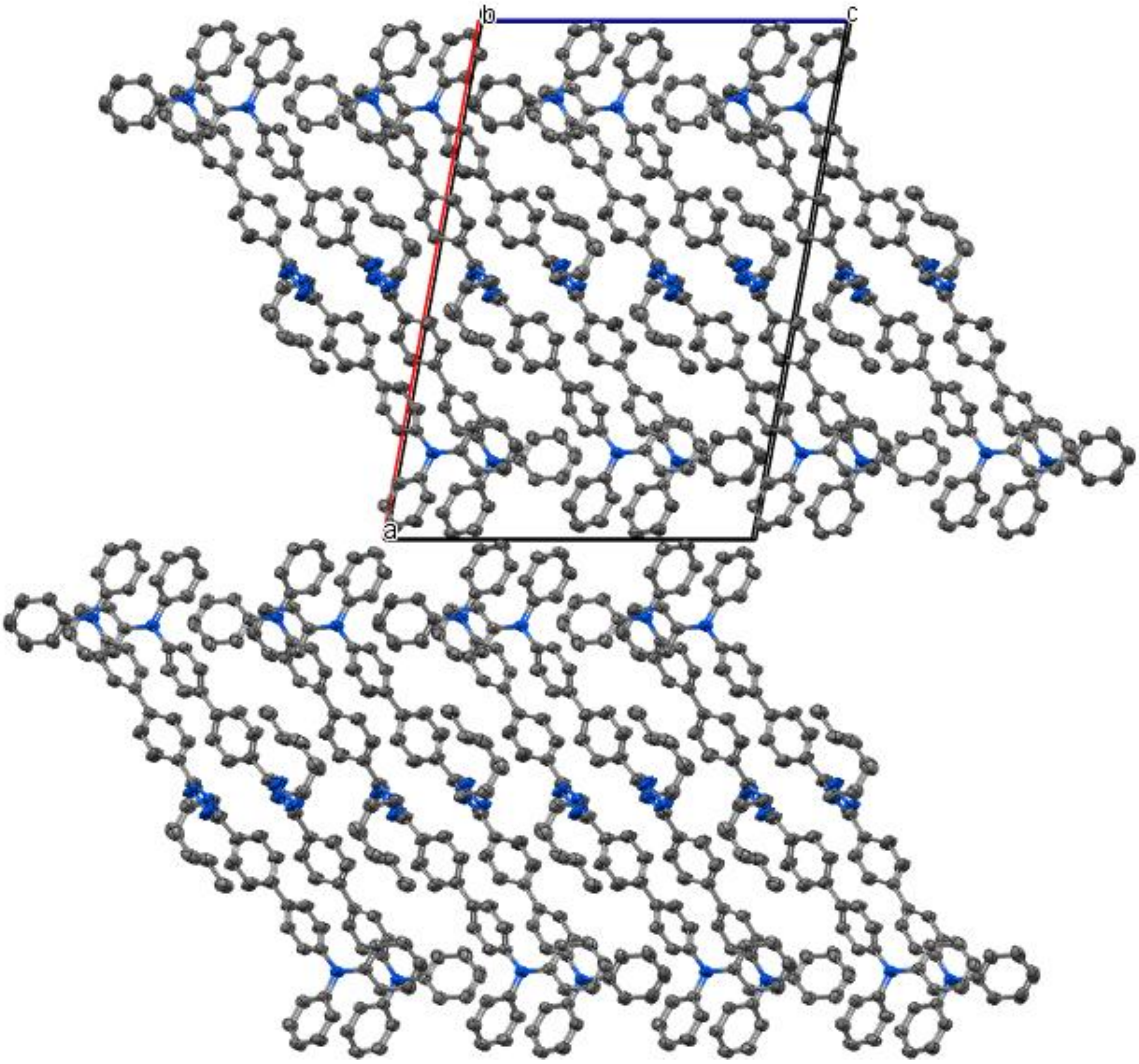

Figure S4. The part of crystal packing in **10a**

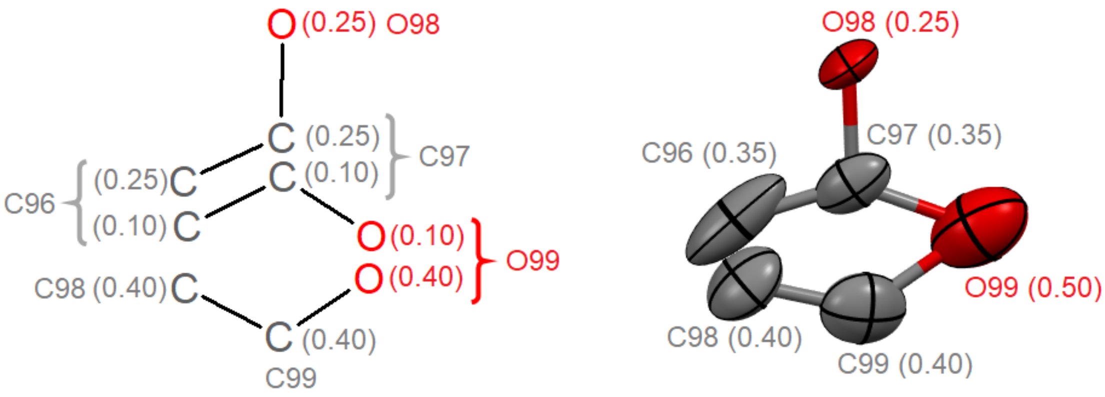

Figure S5. Disorder model of solvent in **8a.**

Table S1. Crystal data and structure refinement details for **8a**, **9a**, and **10a**. The structures of **8a** and **9a** were refined twice: against measured and squeezed data.

Compound	**8a**		**9a**		**10a**
Refinement data	as measured	squeezed	as measured	squeezed	as measured
CCDC number	1999469	1999471	1999470	1999472	1999468
Empirical formula	$C_{54.5}H_{43}N_5O_{0.75}$	$C_{53}H_{43}N_5$	$C_{56}H_{45}N_5O$	$C_{54}H_{45}N_5$	$C_{56}H_{49}N_5$
Formula weight	779.94	749.92	803.97	763.95	792.00
Crystal system	Monoclinic		Monoclinic		Monoclinic
Space group	$P2_1/n$ (No. 14)		$P2_1/n$ (No. 14)		$P2_1/c$ (No. 14)
Temperature (K)	100.0(1)		100.0(1)		100.0(1)
Wavelength (Å)	λ(Cu$K\alpha$) 1.54184		λ(Cu$K\alpha$) 1.54184		λ(Cu$K\alpha$) 1.54184
Unit cell dimensions					
a (Å)	21.1286(2)		21.1879(2)		26.7716(3)
b (Å)	9.6119(1)		9.7481(1)		8.6434(1)
c (Å)	23.0090(2)		22.9643(2)		18.7186(2)
α (°)	90		90		90
β (°)	115.057(1)		115.7160(1)		100.406(1)
γ (°)	90		90		90
Volume ($Å^3$)	4233.03(8)		4273.31(8)		4260.20(8)
Z	4		4		4
Calculated density (Mg/m^3)	1.224	1.177	1.250	1.187	1.235
Absorption coefficient (mm^{-1})	0.569	0.535	0.583	0.538	0.557
F(000)	1644	1584	1696	1616	1680
Crystal size (mm)	0.226 x 0.173 x 0.082		0.102 x 0.072 x 0.043		0.152 x 0.131 x 0.020
θ Range for data collection (°)	3.739 to 78.853		3.771 to 79.032		4.794 to 79.073
Index ranges	$-26 \leq h \leq 26$, $-12 \leq k \leq 10$, $-29 \leq l \leq 27$		$-13 \leq h \leq 26$, $-12 \leq k \leq 11$, $-28 \leq l \leq 23$		$-34 \leq h \leq 32$, $-10 \leq k \leq 8$, $-23 \leq l \leq 21$
Reflections collected / unique	70333 / 8633		44845 / 8827		44854 / 8789
R_{int}	0.0329	0.0330	0.0294	0.0297	0.0484
Completeness (%) $\theta = 67°$	100.0		100.0		100.0
Min. and max. transmission	0.634 and 1.000		0.915 and 1.000		0.852 and 1.000
Data / restraints / parameters	8633 / 0 / 576	8633 / 0 / 525	8827 / 0 / 604	8827 / 0 / 553	8789 / 0 / 598
Goodness-of-fit on F^2	1.073	1.075	1.057	1.111	1.016
Final R indices [I>2σ(I)]	$R1$ = 0.0483, $wR2$ = 0.1504	$R1$ = 0.0423, $wR2$ = 0.1174	$R1$ = 0.0477, $wR2$ = 0.1303	$R1$ = 0.0434, $wR2$ = 0.1175	$R1$ = 0.0560, $wR2$ = 0.1372
R indices (all data)	$R1$ = 0.0525, $wR2$ = 0.1553	$R1$ = 0.0462, $wR2$ = 0.1196	$R1$ = 0.0574, $wR2$ = 0.1481	$R1$ = 0.0517, $wR2$ = 0.1339	$R1$ = 0.0716, $wR2$ = 0.1479
Largest diff. peak and hole (e•$Å^{-3}$)	0.685 and -0.230	0.315 and -0.223	0.449 and -0.268	0.261 and -0.255	0.359 and -0.252

Table S2. Selected structural data of **8a**, **9a**, and **10a**.

i—j	d_{ij} (Å)		
	**8a**	**9a**	**10a**
N1—N2	1.3893(16)	1.3932(17)	1.385(2)
N1—C2	1.3132(19)	1.314(2)	1.311(4)
N2—C1	1.311(2)	1.316(2)	1.317(4)
N3—C1	1.3701(18)	1.3731(19)	1.376(3)
N3—C2	1.3699(17)	1.3725(19)	1.382(3)
N3—C51	1.4740(18)	1.470(2)	1.455(4)
C1—C3	1.4801(18)	1.474(2)	1.473(3)
C2—C27	1.4752(18)	1.472(2)	1.477(3)
N4—C12	1.4156(17)	1.4169(19)	1.4222(19)
N4—C15	1.416(2)	1.412(2)	1.421(2)
N4—C21	1.420(2)	1.425(2)	1.412(2)
N5—C36	1.4360(17)	1.433(2)	1.432(2)
N5—C39	1.416(2)	1.412(2)	1.430(2)
N5—C45	1.4102(19)	1.408(2)	1.416(2)
**i—j—k**	**α_{ijk} (°)**		
	**8a**	**9a**	**10a**
N1—C2—N3	110.63(12)	110.56(13)	110.96(18)
C2—N1—N2	107.22(11)	107.34(13)	107.2(2)
N1—N2—C1	107.07(12)	106.99(13)	107.5(2)
N2—C1—N3	110.84(12)	110.69(13)	110.63(19)
C1—N3—C2	104.24(12)	104.41(13)	103.7(2)
C1—N3—C51	125.98(11)	126.08(12)	127.44(19)
C2—N3—C51	127.65(12)	127.52(12)	127.04(19)
N2—C1—C3	124.63(13)	124.76(14)	121.6(2)
N3—C1—C3	124.49(13)	124.51(14)	127.8(3)
N1—C2—C27	123.91(12)	124.04(14)	122.8(2)
N3—C2—C27	125.46(13)	125.39(14)	126.3(3)
C12—N4—C15	121.25(13)	121.35(14)	118.13(14)
C12—N4—C21	119.19(12)	118.77(13)	119.29(13)
C15—N4—C21	119.02(11)	119.28(13)	122.12(13)
C36—N5—C39	116.77(12)	116.59(14)	115.53(14)
C36—N5—C45	119.22(12)	119.36(14)	120.45(14)
C39—N5—C45	123.44(12)	123.87(13)	122.10(13)

Table S3. Dihedral angles (°) between ring least squares planes in **8a**, **9a**, and **10a**. Each ring is indicated by one atom, which belongs solely to this ring.

8a

	N1	C3	C9	C15	C21	C27	C33	C39
N1								
C3	54.83(5)							
C9	31.83(5)	23.01(8)						
C15	88.73(6)	40.45(6)	61.11(6)					
C21	84.44(6)	59.81(6)	74.27(6)	65.52(5)				
C27	42.78(5)	25.79(7)	20.77(8)	49.57(6)	85.55(5)			
C33	11.73(5)	47.94(5)	25.62(5)	81.28(6)	83.12(5)	31.95(6)		
C39	77.76(6)	71.15(5)	83.41(5)	38.51(5)	78.79(4)	63.67(5)	89.43(5)	
C45	81.09(6)	44.14(5)	67.09(6)	18.06(3)	48.78(6)	61.46(5)	89.02(6)	52.80(5)

9a

	N1	C3	C9	C15	C21	C27	C33	C39
N1								
C3	54.70(6)							
C9	29.44(5)	25.34(8)						
C15	88.84(7)	40.28(6)	62.83(7)					
C21	85.80(6)	60.01(6)	75.98(6)	66.52(6)				
C27	45.01(6)	26.30(8)	23.19(8)	47.79(7)	86.30(5)			
C33	17.11(5)	43.74(6)	19.91(6)	75.89(7)	85.08(6)	28.51(7)		
C39	74.57(6)	73.95(5)	87.22(6)	40.60(6)	76.67(5)	64.41(6)	88.68(6)	
C45	81.02(7)	44.36(6)	69.54(7)	17.31(3)	50.87(7)	60.15(6)	86.18(7)	53.10(5)

10a

	N1	C3	C9	C15	C21	C27	C33	C39
N1								
C3	36.3(1)							
C9	24.8(1)	12.3(1)						
C15	85.21(8)	63.32(7)	71.51(5)					
C21	84.22(7)	65.48(7)	77.22(5)	61.31(5)				
C27	40.8(1)	12.0(1)	16.7(1)	54.83(6)	70.26(6)			
C33	23.4(1)	17.7(1)	6.7(1)	71.47(6)	83.14(5)	17.8(1)		
C39	85.51(7)	67.75(7)	79.18(5)	65.62(5)	4.4(1)	73.23(6)	85.31(5)	
C45	88.76(8)	57.73(7)	67.03(5)	8.38(8)	54.06(5)	50.50(6)	67.90(6)	58.43(6)

Table S4. Non-classic hydrogen bonds and the first level graph motifs in the studied compounds (Å, °).

D-H•••A	d(D-H)	d(H•••A)	d(D•••A)	<(DHA)	$G_d^a(n)$
			**8a**		
C8—H8•••N1^{i}	0.95	2.59	3.492(2)	158	C(6)
C32—H32•••N2^{i}	0.95	2.76	3.702(2)	174	C(6)
C41—H41•••N2^{ii}	0.95	2.79	3.481(2)	130	C(16)
C51—H51B•••N1^{i}	0.99	2.80	3.575(2)	136	C(5)
			**9a**		
C8—H8•••N1^{i}	0.95	2.68	3.573(2)	157	C(6)
C32—H32•••N2^{i}	0.95	2.69	3.640(2)	175	C(6)
C41—H41•••N2^{ii}	0.95	2.90	3.572(2)	129	C(16)
C51—H51B•••N1^{i}	0.99	2.81	3.529(2)	130	C(5)
			**10a**		
C32—H32•••N1^{iii}	0.95	2.55	3.470(2)	164	C(5)
C32—H32•••N2^{iii}	0.95	2.43	3.163(2)	134	C(6)

Symmetry transformations used to generate equivalent atoms: (i) -x+1.5, y-0.5, -z+1.5; (ii) -x+1, -y+1, -z+1; (iii) -x+1, y+0.5, -z+1.5.

Table S5. Stacking interactions in the studied compounds. Each ring is indicated by one atom, which belongs solely to this ring. The α is a dihedral angle between planes I and J, β is an angle between Cg(I)-Cg(J) vector and normal to plane I, d_p is a perpendicular distance of Cg(I) on ring J plane.

R(I)•••R(J)	d(Cg•••Cg) (Å)	α (°)	β (°)	d_p (Å)
		**8a**		
C15•••C45^{i}	4.8776(10)	5.60(8)	45.6	3.1070(7)
C45•••C15^{ii}	4.8776(10)	5.60(8)	50.4	3.4134(7)
C21•••C21^{iii}	5.7439(11)	0.02(9)	56.6	3.1589(7)
C45•••C45^{iv}	4.7511(10)	0.00(8)	45.9	3.3089(7)
		**9a**		
C15•••C45^{i}	4.9048(11)	6.31(9)	44.4	3.1458(8)
C45•••C15^{ii}	4.9050(11)	6.31(9)	50.1	3.5026(8)
C21•••C21^{iii}	5.7876(12)	0.00(10)	55.9	3.2444(8)
C45•••C45^{iv}	4.8010(11)	0.02(9)	45.0	3.3964(8)
		**10a**		
N1•••N1^{v}	5.4759(13)	0.00(13)	42.9	4.0097(9)
C21•••C39^{vi}	5.3990(10)	4.41(9)	47.0	3.9724(7)
C39•••C21^{vii}	5.3991(10)	4.41(9)	42.6	3.6798(8)

Symmetry transformations used to generate equivalent atoms: (i) -x+1.5, y+0.5, -z+1.5; (ii) -x+1.5, y-0.5, -z+1.5; (iii) -x+3, -y, -z+2; (iv) -x, -y, -z+1; (v) -x+1, -y+1, -z+1; (vi) x-1, y, z-1; (vii) x+1, y, z+1.

4. Absorption and emission spectrometry data

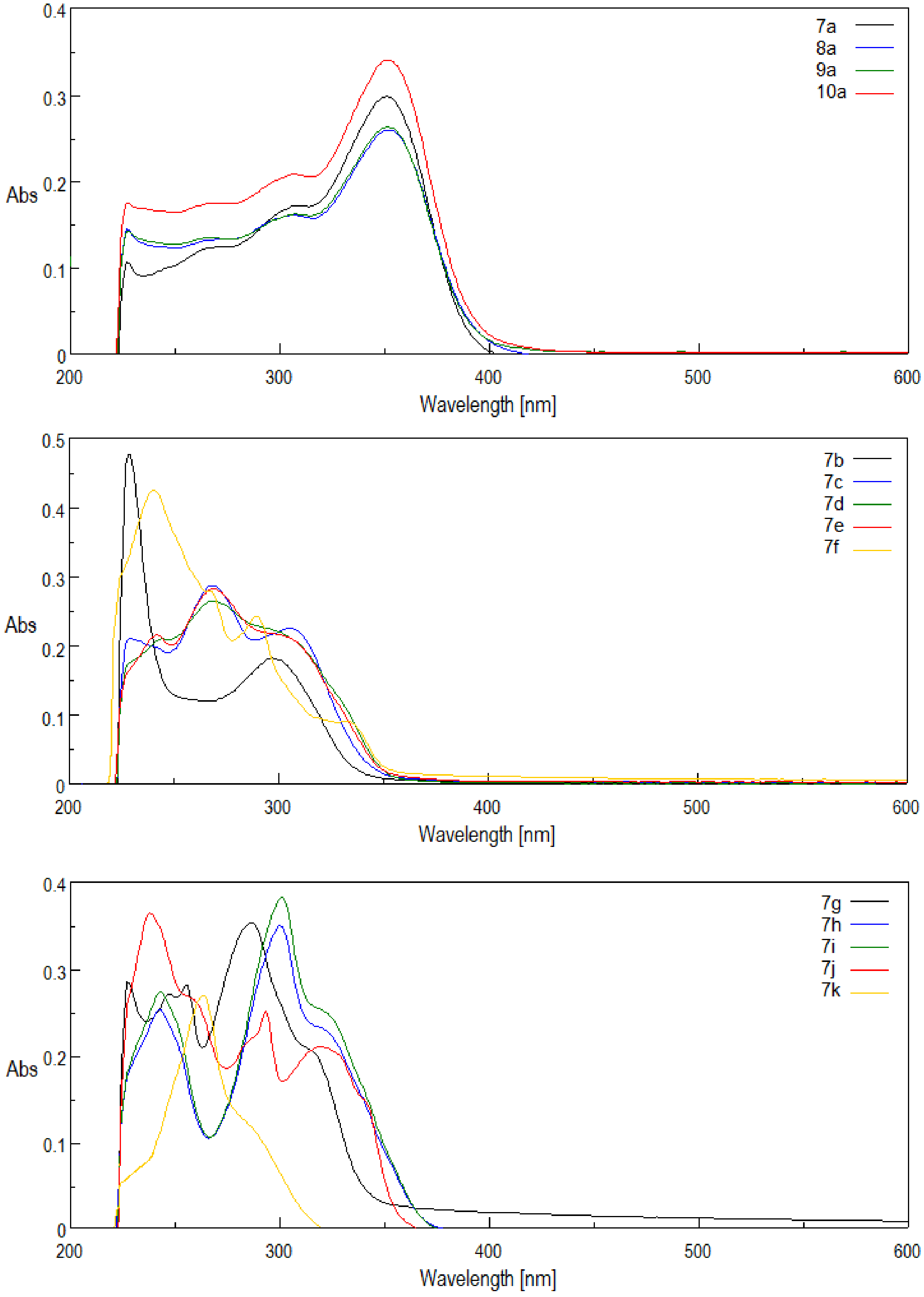

Figure S6. UV-Vis spectra of **7a-10a** and **7b-7k**.

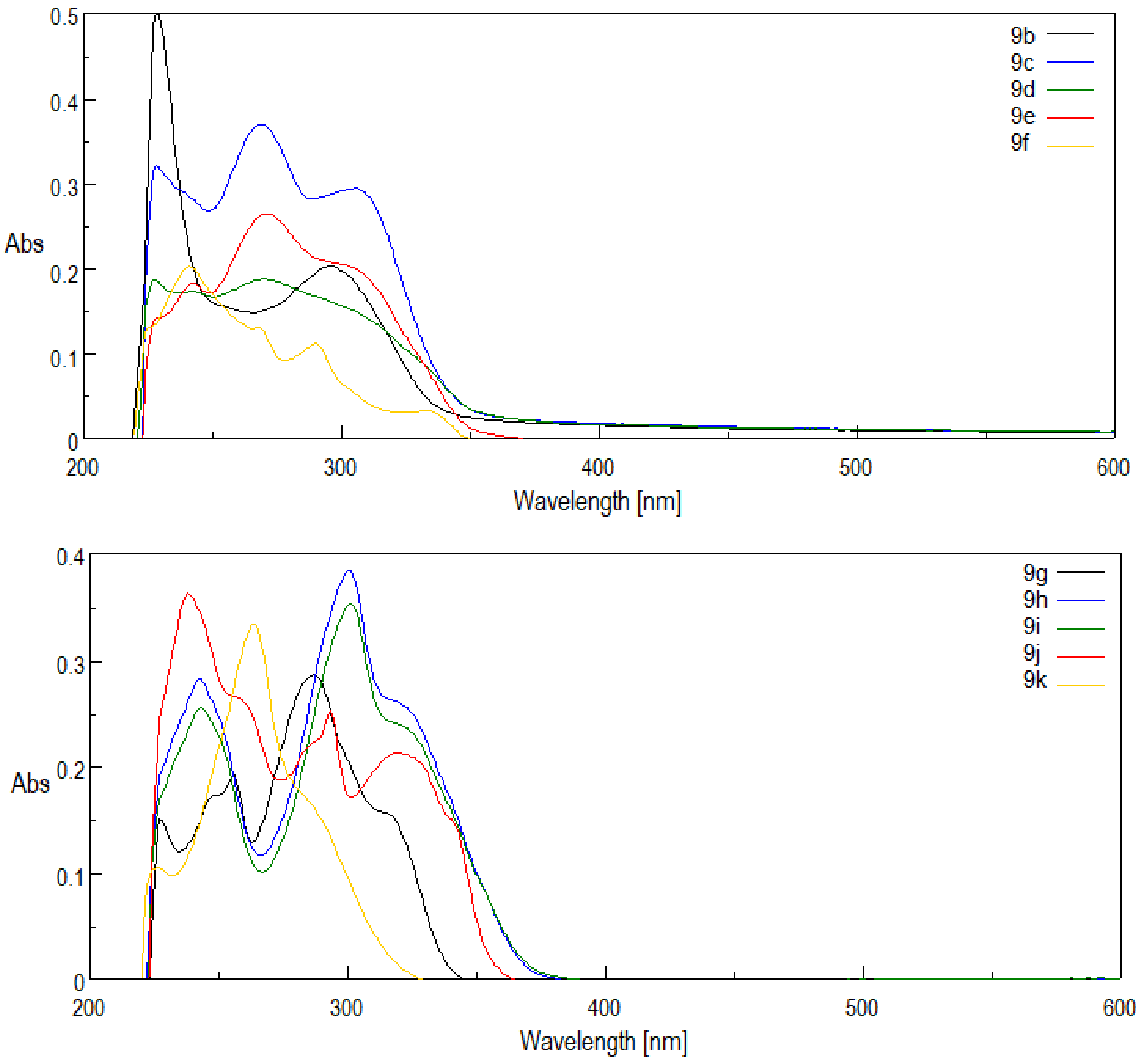

Figure S7. UV-Vis spectra of **9b**-**9k**.

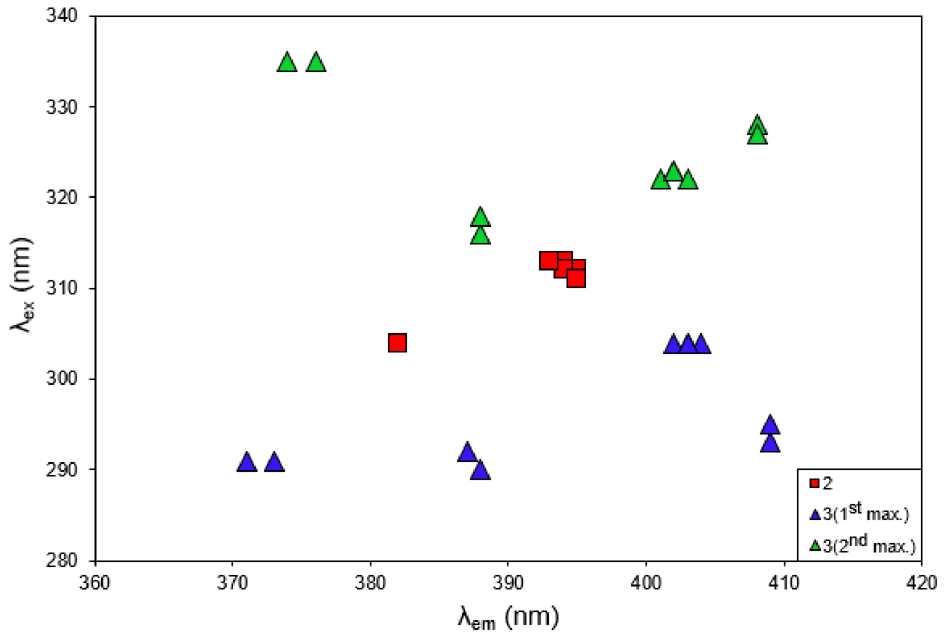

Figure S8. Positions of global maxima for studied compounds (divided into groups containing 2 or 3 rings at the ends of 4-alkyl-3,5-bis(phenyl)-4*H*-1,2,4-triazole core). "1st max." and "2nd max." indicate the first (for smaller excitation wavelengths) and the second (for larger excitation wavelengths) maxima on three-dimensional fluorescence spectra.

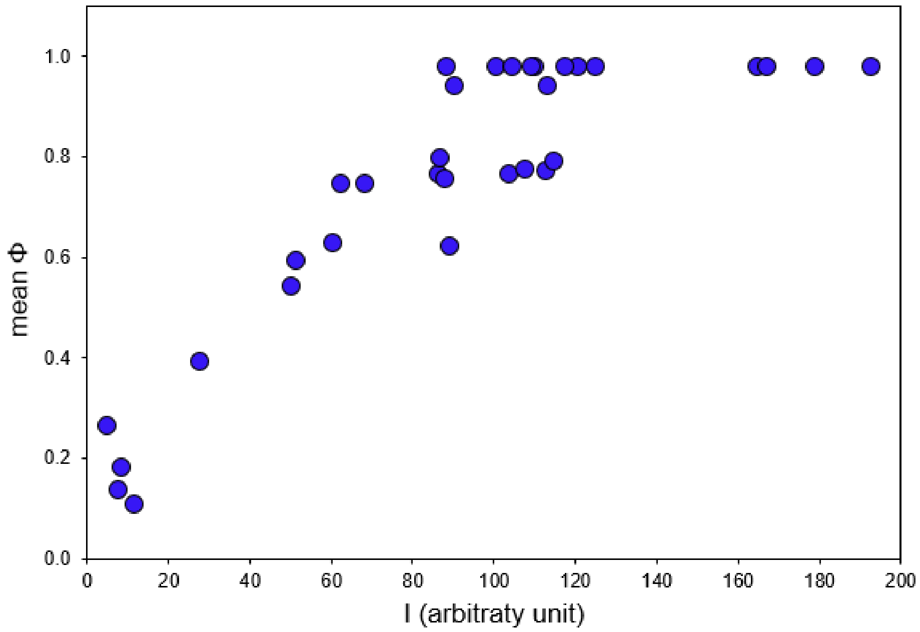

Figure S9. Quantum yield of studied compounds as a function of fluorescence intensity at global and local maximum.

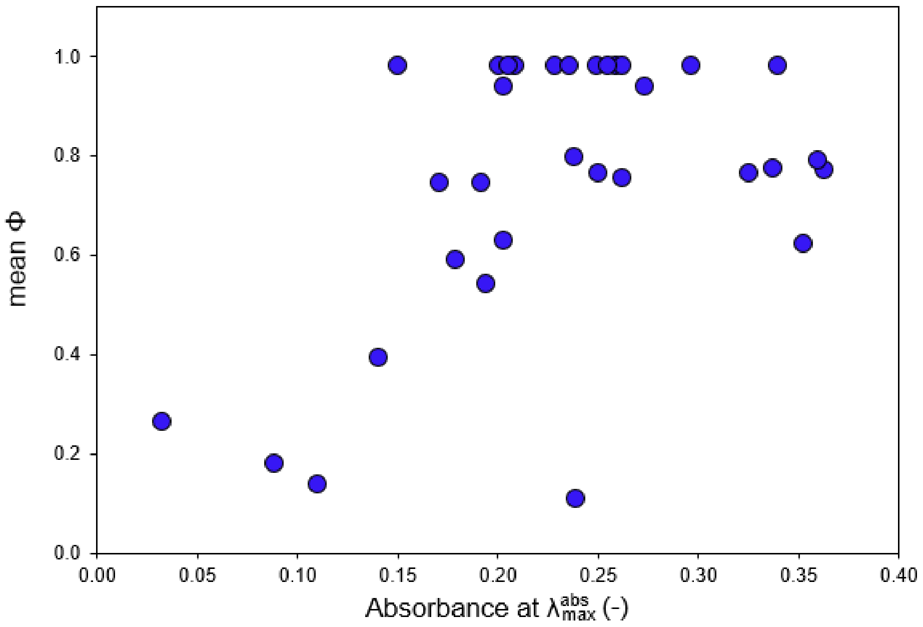

Figure S10. Quantum yield of studied compounds in relation to absorption at global and local maximum of fluorescence.
